# Temperature shapes coral-algal symbiosis in the South China Sea

**DOI:** 10.1038/srep40118

**Published:** 2017-01-13

**Authors:** Haoya Tong, Lin Cai, Guowei Zhou, Tao Yuan, Weipeng Zhang, Renmao Tian, Hui Huang, Pei-Yuan Qian

**Affiliations:** 1 HKUST Shenzhen Research Institute and Division of Life Science, Hong Kong University of Science and Technology, Clear Water Bay, Hong Kong SAR, China; 2 Key Laboratory of Tropical Marine Bio-resources and Ecology, South China Sea Institute of Oceanology, Chinese Academy of Sciences, China; 3 Tropical Marine Biological Research Station in Hainan, Chinese Academy of Sciences, China

## Abstract

With the increase in sea surface temperature (SST), scleractinian corals are exposed to bleaching threats but may possess certain flexibilities in terms of their associations with symbiotic algae. Previous studies have shown a close symbiosis between coral the and *Symbiodinium*; however, the spatial variation of the symbiosis and the attribution underlying are not well understood. In the present study, we examined coral-algal symbiosis in *Galaxea fascicularis* and *Montipora* spp. from three biogeographic regions across ~10° of latitude in the South China Sea. Analysis of similarities (ANOSIM) indicated a highly flexible coral-algal symbiosis in both *G. fascicularis* and *Montipora* spp. and canonical correspondence analysis (CCA) showed that temperature explained 83.2% and 60.1% of the explanatory subclade variations in *G. fascicularis* and *Montipora* spp., respectively, which suggested that temperature was the main environmental factor contributing to the diversity of *Symbiodinium* across the three regions. The geographic specificity of the *Symbiodinium* phylogeny was identified, revealing possible environmental selection across the three regions. These results suggest that scleractinian corals may have the ability to regulate *Symbiodinium* community structures under different temperatures and thus be able to adapt to gradual climate change.

Under global climate change, coral reefs are greatly threatened by reduced productivity and the precipitation of calcium carbonate^1^,^2^. The symbiotic dinoflagellates (*Symbiodinium*) in corals contribute approximately 95% of the coral energy requirements^3^ and play a central role in coral reef maintenance and productivity. In addition, coral reef resilience to future climate change strongly relies on the adaptation of coral-algal symbiosis. According to previous studies, a continuous loss of *Symbiodinium*, such as a bleaching event, dramatically increases coral mortality. Hence, an understanding of coral-algal symbiosis change in response to different environmental conditions is essential to develop coral reef conservation strategies under future climate change.

The genus *Symbiodinium* consists of nine clades (i.e., clades A–I) with varied photosynthetic efficiencies and heat tolerance abilities^4^. Although clades A–D are the main *Symbiodinium* harbored by scleractinian corals, some other clades, such as F/G, have also been found in scleractinian corals and have been proposed to exert certain functional roles in the coral reef ecosystem^5^. The flexibility of coral-algal symbiosis is widely accepted^6^,^7^,^8^. Because corals are able to change their *Symbiodinium* communities in response to diverse environmental stressors, for example, by gaining heat-tolerant *Symbiodinium* in an environment with an elevated temperature, the newly formed coral-algal symbiosis has been hypothesized to be more beneficial for coral survival^9^. In contrast, on a large latitude scale, some coral species still maintain a very stable coral-algal symbiosis^10^. Thus, the capacity for coral-algal symbiosis change in response to environmental change remains controversial.

Temperature has been shown to have a great impact on coral-algal symbiosis^11^,^12^. At extremely high temperatures, *Symbiodinium* might be expelled from the coral host. After recovering from bleaching or remaining a sufficient duration in a relatively warm temperature, the coral host can gain some heat-tolerant *Symbiodinium*^9^. In addition, nutrients, light and salinity all have been shown to affect *Symbiodinium* community structures^13^,^14^,^15^; however, among these environmental factors, the one(s) with the greatest effect remains unclear.

Hong Kong (Table 1) is located at 22°10′N to 22°30′N and has a subtropical climate with low levels of salinity in the summer^16^, in which the sea surface temperature (SST) ranges from 13 to 30 °C which is marginal for hard coral growth^17^. Because of its extreme seasonal temperature changes and relatively low salinity, there are mainly northern marginal corals distributed in Hong Kong. Sanya and Sansha, lying in the southern region of Hainan Province, have a tropical climate with SST ranges from 20 to 30 °C and from 24 to 30 °C, respectively. Coral reefs in Sanya are intermediate and are located near the northern border of the global distribution of tropical coral reefs^18^, whereas coral reefs in Sansha are typically tropical. In general, the average annual SST rises across the three regions with changes in latitude (Fig. 1). The impact of human activities on the five sampling sites is as follows: Lamma Island (LI) > Crescent Bay (CB), Luhuitou (LHT) > Sunny Bay (SB) > Drummond Island (DI). Crescent Bay is located in the northeast of Hong Kong, where there is marginal influence by the Pearl River and the greatest coral cover (30–50%) in Hong Kong has been observed. In contrast, Lamma Island in the southwest of Hong Kong, has a relatively low coral cover (10–30%) due to the influence of the Pearl River^16^. The coral cover in Luhuitou of Sanya decreased from 80–90% in the 1960 s to 11% in 2007 due to frequent human activities; the coral cover around Sunny Bay in Sanya was approximately 35% in the period from 2007–2009^19^. Drummond Island in the Sansha region is one of the Xisha Islands with a coral cover of approximately 50% based on 30-year data from the 1970 s^20^. We selected these five sampling sites with different environmental conditions to examine the change of coral-algal partnerships in response to environmental variations and to identify the environmental factor(s) with the greatest impact.

The diversity of *Symbiodnium* has mostly been examined by denaturing gradient gel electrophoresis (DGGE) based analysis, which is unable to detect *Symbiodinium* subclades with a relative abundance of less than 10% and thus potentially lacks the sufficient sensitivity to detect symbiosis change^7^. The development of highly sensitive molecular techniques, such as high-throughput DNA sequencing, would facilitate the identification of low abundance Symbiodinium that benefit coral hosts in terms of tolerant to climate change. Additionally, amplicon sequencing using ITS2 primers has been shown to be reliable for measurements of *Symbiodinium* communities^10^. In the present study, to explore spatial variations of coral-algal symbiosis, we used ITS2 amplicon sequencing to examine coral-algal symbiosis in *G. fascicularis* and *Montipora* spp. from five sampling sites across three biogeographic regions, including tropical and subtropical seas, and we attempted to elucidate the symbiosis change across these regions. Furthermore, we examined the effects of temperature, depth, salinity etc. on coral-algal symbiosis change.

## Results

### Sequence information

In total, 1,774,246 sequences (more than 80% of the input sequences) were retained for further analysis after quality control. After removing the fusion primers, the median length of the sequences was ~332 bp. The dataset included a total of 58 samples: six samples of *G. fascicularis* from CB(Hong Kong), LHT(Sanya), SB(Sanya) and DI(Sansha) each (CB_GA1-6, LHT_GA1-6, SB_GA1-6, DI_GA1-6), six samples of *Montipora* spp. from LI(Hong Kong) and SB(Sanya) each (LI_MO1-6, SB_MO1-6), four samples of *Montipora* sp. (LHT_MO1-4), two *Montipora* sp. technical replicates (LHT_MO5-6) from LHT(Sanya), five samples of *Montipora* sp. (CB_MO1-5), a *Montipora* sp. (CB_MO6) technical replicate from CB(Hong Kong) and two samples of seawater from each sampling site (CB_SW1-2, LI_SW1-2, LHT_SW1-2, SB_SW1-2, DI_SW1-2). Three *Montipora* spp. samples were failed during DNA extraction or PCR amplification. In total, 131 *Symbiodinium* subclades were assigned based on alignment to the ITS2 database according to 97% similarity (whole_community_data.xls, Supporting Information), and 13 dominant subclades (covering more than 85% of the sequence) were selected for further analyses according to the relative abundance of the *Symbiodinium* subclade, the rest were regarded as “Others”.

### Disparity in *Symbiodinium* community structures of *G. fascicularis* and *Montipora* spp. across the three geographic regions

**Figure 2:**
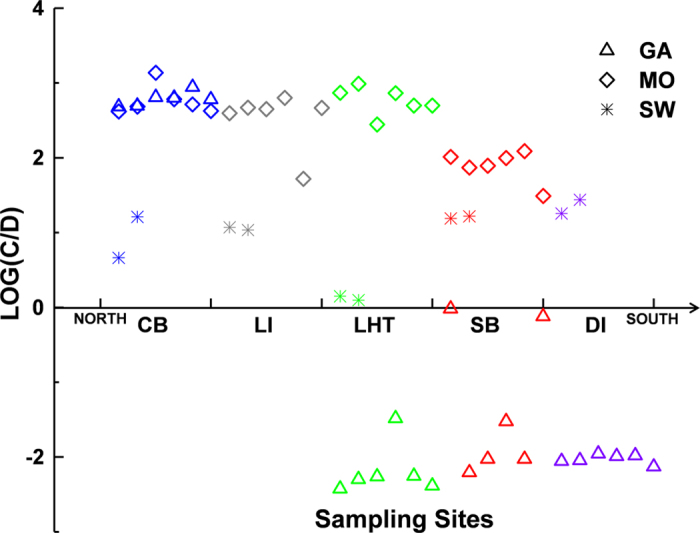
S*ymbiodinium* community compositions at the clade level. Compositions of dominant *Symbiodinium* clades from Crescent Bay (CB, Hong Kong), Lamma Island (LI, Hong Kong), Luhuitou (LHT, Sanya), Sunny Bay (SB, Sanya), Drummond Island (DI, Sansha) in the South China Sea. Triangles, diamonds, stars represent *G. fascicularis* (GA), *Montipora* spp. (MO), and seawater (SW) samples from each site, respectively. Each symbol represents a single sample and the value is calculated by logarithm of the ratio of C clade relative abundance to D clade relative abundance in each sample.

Clade A, clade B, clade C, clade D and clade F were detected in samples. Among all samples, only two seawater samples from Sansha contained 0.06% and 0.6% A clade, respectively. The total relative abundance of clade A, B and F in samples were all less than 0.01%, thus only *Symbiodinium* clade C and D were kept for further analysis. As shown in Fig. 2, *G. fascicularis* in Sanya and Sansha had a high symbiont specificity for clade D, whereas *G. fascicularis* in Hong Kong showed a high symbiont specificity for clade C. In contrast, in all sampling sites, *Montipora* spp. contained a very low abundance of clade D but a high abundance of clade C. All of the seawater samples were dominated by clade C, suggesting *Symbiodinium* selection and enrichment of *G. fascicularis* in Sanya and Sansha.

**Figure 3:**
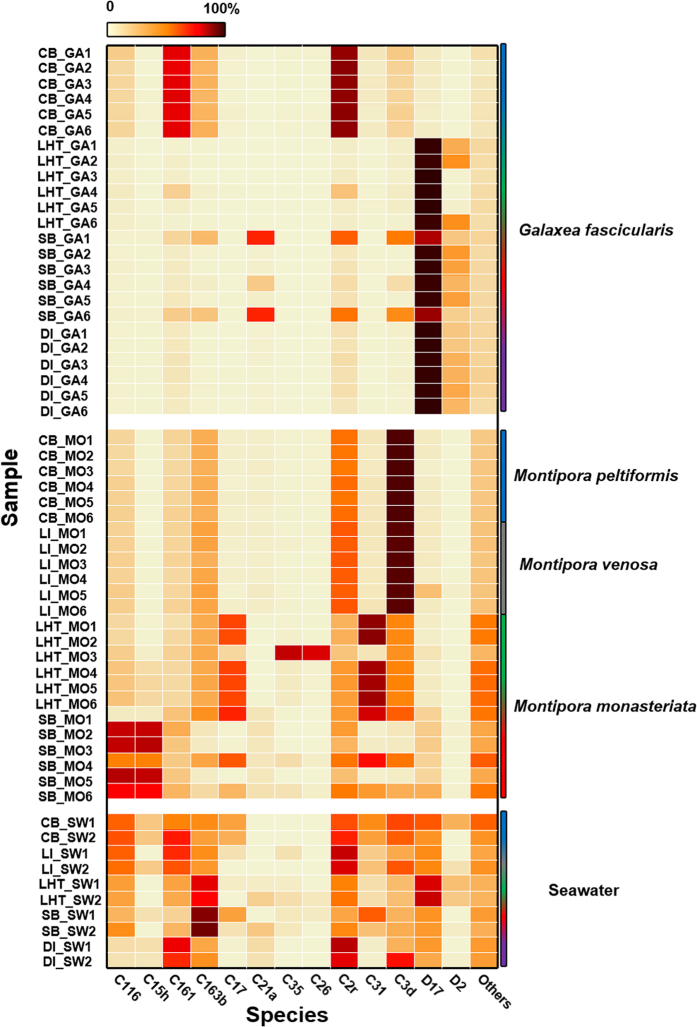
Sy*mbiodinium* community structures at the subclade level. Relative abundance of dominant *Symbiodinium* subclades from *G. fascicularis, Montipora* spp., and seawater samples in the South China Sea. The color scale on the top represents the percentage of a subclade from each sample. The color scale on the right represents samples from different sites, blue, grey, green, red and purple represent samples from Crescent Bay (CB, Hong Kong), Lamma Island (LI, Hong Kong), Luhuitou (LHT, Sanya), Sunny Bay (SB, Sanya), Drummond Island (DI, Sansha), respectively. Among the selected *Symbiodinium* subclades, C17.2, C26.b1, and C21 are equivalent to C17, C35, and C3d, respectively. C26a and C35a are both equivalent to C26.

At the subclade level, 13 dominant/subdominant subclades were found, accounting for more than 85% of the total sequences (Fig. 3). These subclades were picked as their relative abundances were >10% in at least one sample or their average relative abundances in all samples were at least around 1%. In *G. fascicularis*, C161, C21a, C2r, D17 were regarded as dominant subclades with abundances >10%, whereas C163b, C3d and D2 were subdominant with abundances ranging from 1–10%. In *Montipora* spp., a total of 8 subclades, C116, C15h, C17, C35, C26, C2r, C31 and C3d, could become dominant dependent on the location. Based on an overview of Fig. 3, although *Symbiodinium* in *Montipora* spp. did not differ much across regions at the clade level, *M. monasteriata* exhibited more different *Symbiodinium* subclade compositions in two sites of Sanya (LHT and SB), whereas *Montipora* spp. still displayed very similar *Symbiodinium* subclade compositions in two sites of Hong Kong (CB and LI), supporting the notion that the coral-algal symbiosis is very flexible across host species and geographic distance. The non-parametric multivariate analysis of similarity (ANOSIM) demonstrated that *Symbiodinium* community structures differed significantly between *G. fascicularis* and *Montipora* spp., as well as between coral and seawater samples (*R* > 0.8, *P* = 0.01). Moreover, the *Symbiodinium* community structures among the three regions (Hong Kong, Sanya, and Sansha) were significantly different (*R* > 0.69, *P* ≤ 0.03), whereas less significant variations were observed between sampling sites in the same region (CB and LI (Hong Kong), *R* = 0.257; LHT and SB (Sanya), *R* = 0.559). These findings indicated a possible environmental adaption across regions and strict host specificity of the *Symbiodinium* community. Furthermore, most of the subclades identified in corals were also found in the surrounding seawater, suggesting the free-living ability of *Symbiodinium* and a possible horizontal transmission of *Symbiodinium* between seawater and corals. Some dominant subclades demonstrated much higher relative abundances in corals compared with those in seawater, suggesting an enrichment of certain *Symbiodinium* subclades in corals, which is consistent with the findings at the clade level.

**Figure 4:**
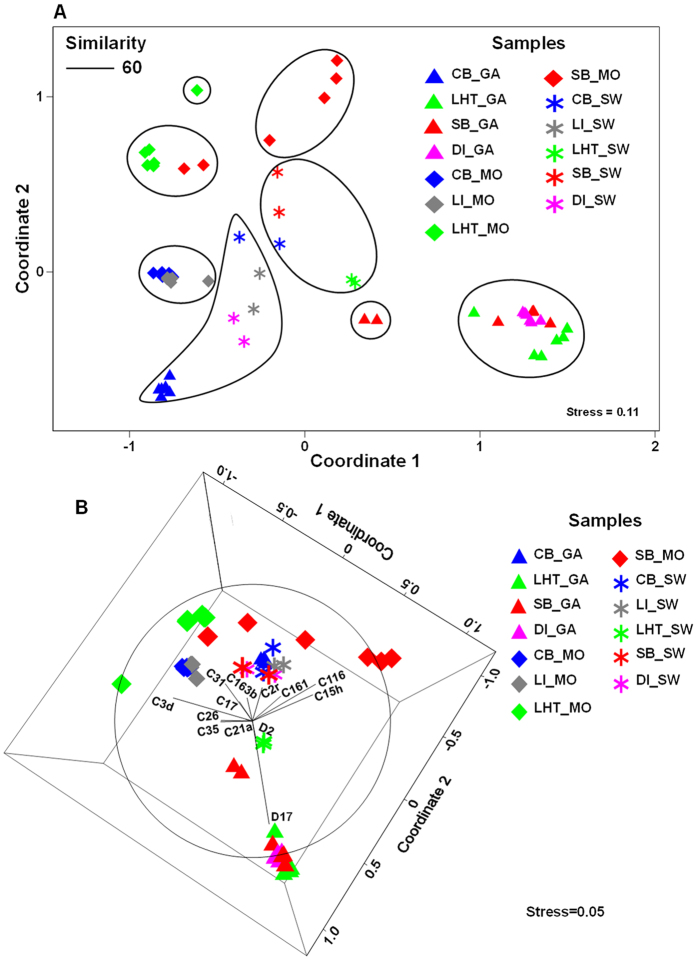
nMDS ordinations. nMDS plot of *G. fascicularis* and *Montipora* spp. dominant *Symbiodinium* subclade community compositions based on the square root-transformed relative abundance matrix by the Bray-Curtis measure of dissimilarity. Each symbol represents a single sample. Kruskal’s stress numbers are provided. (**A**): 2dnMDS, with grouping based on complete linkage cluster analysis of 60% similarity. (**B**): 3dnMDS, with a dominant *Symbiodinium* subclade overlay vector.

The results of non-metric multidimensional scaling (nMDS) revealed a clear clustering pattern, supporting the dissimilarities among all samples (Fig. 4). All of the seawater samples were located between the *G. fascicularis* and *Montipora* spp. samples. Based on a similarity of 60% (Fig. S1, Supporting Information), the *Symbiodinium* communities of *G. fascicularis* in Hong Kong were quite different from those in Sanya and Sansha. The *Symbiodinium* communities of *Montipora* spp. were less different within the same region (CB and LI (Hong Kong), LHT and SB (Sanya)), but exhibited more differences between different regions (Hong Kong and Sanya). *G. fascicularis* in Hong Kong and all *Montipora* spp. were dominated by diverse C subclades, whereas *G. fascicularis* in Sanya and Sansha mainly harbored two D subclades.

In summary, different coral species at the same sampling site appeared to prefer different *Symbiodinium* subclades. Congeneric corals from different regions also tended to have significantly distinct *Symbiodinium* community compositions despite fewer changes in *Symbiodinium* community compositions of the surrounding seawater. These results suggested that, for congeneric corals, coral-algal symbiosis change mainly resulted from environmental changes across the investigated regions.

### Correlations between environmental factors and *Symbiodinium* communities

**Figure 5:**
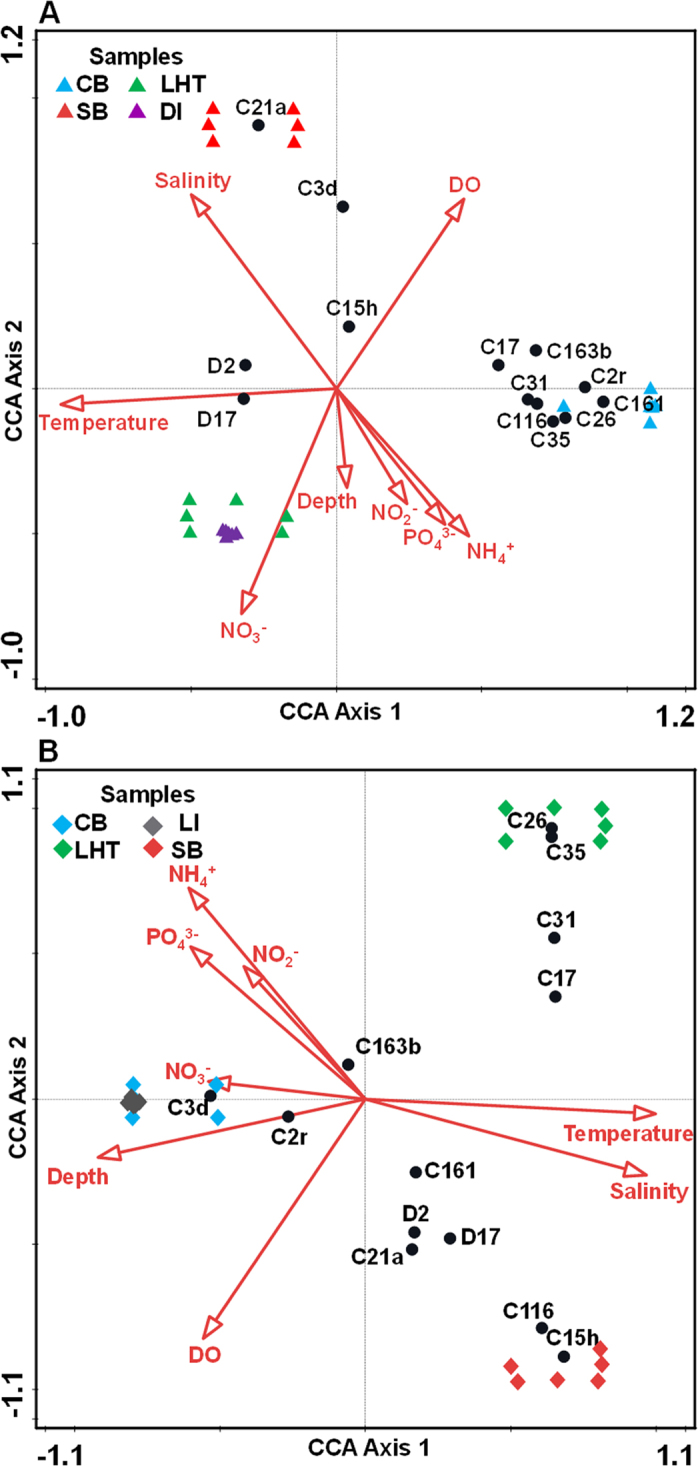
Relationships among environmental factors, *Symbiodinium* subclades, and sampling sites. CCA indicates the relationship among environmental factors, *Symbiodinium* subclades, and coral *Symbiodinium* community composition from different sampling sites. (**A**) CCA of *G. fascicularis* samples. The first axis (CCA Axis 1) explains 75.88% of the total variation and 92.71% of the fitted variation; the second axis (CCA Axis 2) explains 5.96% of the total variation and 7.28% of the fitted variation. (**B**) CCA of *Montipora* spp. samples. CCA Axis 1 explains 31.72% of the total variation and 60.18% of the fitted variation; CCA Axis 2 explains 20.89% of the total variation and 39.63% of the fitted variation.

According to the canonical correspondence analysis (CCA), temperature, salinity and NH_4_^+^ served as the main factors among all potential impact factors (Fig. S2, Supporting Information) affecting *Symbiodinium* subclades and community compositions, explaining 81.8% and 52.7% of the total subclade variations in *G. fascicularis* (Fig. 5A) and *Montipora* spp. (Fig. 5B), respectively. This finding indicated that these three factors provided the largest contribution to coral-algal symbiosis change in the present study. Remarkably, temperature was the most significant factor in shaping subclade compositions, explaining 83.2% and 60.1% of the explanatory variations in *G. fascicularis* and *Montipora* spp., respectively. This finding suggested that coral-algal symbiosis change in studied samples was largely driven by temperature. In *G. fascicularis*, two D subclades (D2, D17) were positively correlated to temperature, while most C subclades had negative correlation with temperature, indicating that *G. fascicularis* was more likely to select heat-tolerant *Symbiodinium* with an increasing temperature because the D clade is believed to be more tolerant to heat^21^,^22^. In *Montipora* spp., only three C subclades (C2r, C3d and C163b) were negatively correlated to temperature, whereas the other dominant subclades were all correlated positively to temperature. In both plots, samples from Hong Kong (CB, LI) were clearly separated from those from Sanya and Sansha along axis 1, which was highly correlated to temperature, indicating that temperature is a major factor in shaping *Symbiodinium* community structures between subtropical (Hong Kong) and tropical (Sanya and Sansha) regions. Thus, temperature provided the greatest environmental contribution to the coral-algal change between subtropical and tropical regions. However, the samples from the tropical region were divided along axis 2, which was mainly correlated to kinds of nutrients, suggesting that nutrients are minor environmental factors affecting *Symbiodinium* community structures under similar temperature conditions.

### Phylogenetic relationships of dominant *Symbiodinium* subclades from *G. fascicularis* and *Montipora* spp. in the three regions

**Figure 6:**
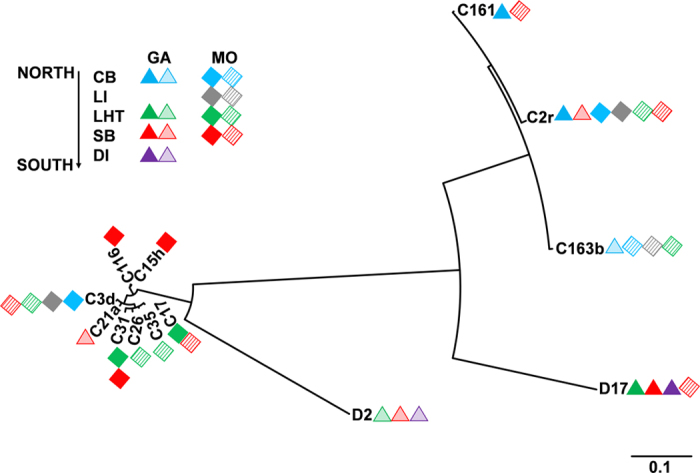
Phylogenetic tree of *Symbiodinium* subclades that are dominant in *G. fascicularis* and *Montipora* spp. in the South China Sea. Every symbol represents a group in which the average relative abundance of a certain subclade is >1%. Solid and non-solid symbols indicate average relative abundances of >10% and 1–10%, respectively. The phylograms were analyzed with ITS2 sequences using maximum likelihood and Bayesian inference analyses, and nearly identical profiles were obtained. The presented tree is derived from Bayesian inference.

*Symbiodinium* subclades dominating congeneric corals tended to be closely distributed in the phylogenetic tree (Fig. 6). For example, C161, C2r, and C163b from *G. fascicularis* in Hong Kong had closer phylogenetic relationships with D2/D17 from *G. fascicularis* in Sanya and Sansha compared with the other C subclades that were symbiotic with *Montipora* spp., suggesting a possible coral-algal coevolution. For the congeneric corals, *Symbiodinium* associated with corals with a closer geographic distance demonstrated closer phylogenetic relationships, revealing that the phylogenetic relationships of *Symbiodinium* were linked to geographical distances.

## Discussion

Here, we presented the first systematic investigation of *Symbiodinium* communities in different coral species across tropical and subtropical regions and indicated the highly spatial flexibility of coral-algal symbiosis in *G. fascicularis* and *Montipora* spp. in the South China Sea. Although several previous studies have investigated *Symbiodinium* community structures of certain scleractinian corals in the South China Sea^23^,^24^, this is the first study to demonstrate that, among all potential environmental factors, temperature plays the major role in shaping *Symbiodinium* community structures and coral-algal symbiosis. The results of the present study are consistent, in part, with some previous findings. Temperature^9^,^25^,^26^, nutrients^27^, and depth^28^ all have the potential to affect either the coral host or *Symbiodinium* community structures, but none of those studies compared the relative effects of these factors or their effects on different coral species. Cooper *et al*.^11^ showed, mud content, carbonate content, SST and depth together explained 51.3% of the total variations in dominant *Symbiodinium* communities in *A. millepora* and long-term SST explained 10.8% of the total variations. However, they only examined *Symbiodinium* C1, C2 and D1, whereas we detected 131 *Symbiodinium* subclades and 13 dominant *Symbiodinium* subclades in the present study, suggesting a more in-depth coverage of the *Symbiodinium* communities.

### Temperature shapes the structures of the *Symbiodinium* community

The concordance of the taxonomic data with phylogenetic and ecological evidence led us to propose that, among all potential environmental factors, temperature acted as the major environmental factor impacting coral-algal symbiosis spatial change in the South China Sea. The important role of temperature and the underlying mechanisms can be partly explained by previous empirical evidence. For example, extremely high temperatures can lead to coral bleaching^29^, but corals that contained more heat-tolerant *Symbiodinium* were less likely to bleach and bleached corals gained more heat-tolerant *Symbiodinium* after recovery^22^,^30^. Coral larvae gained different *Symbiodinium* when treated with different temperatures^31^, whereas adult corals acquired more heat-tolerant *Symbiodinium* in response to thermal stress^9^. Additionally, corals from some extremely hot regions have been found to carry some highly heat-tolerant *Symbiodinium*^15^. These findings support the conclusion that temperature is a significant factor affecting coral-algal symbiosis. Using a network analysis of stressor interactions, Ban *et al*.^12^ suggested that sedimentation, storms and temperature were the most influential stressors in the coral reef ecosystem, which also indicated that temperature could drive coral-algal symbiosis change and play an important role in the establishment of coral-algal symbiosis.

Hong Kong environmental data was collected from HKEPD which was monitored less than one week before the sampling time. As *Symbiodinium* community in a given coral colony can remain relatively stable in a period of time^32^, results shall not be affected much. Sansha sampling was finished two months later than the others, it would cause problems when comparing communities between different sites, while *Symbiodinium* communities in *G. fascicularis* from Sansha were still highly similar with those in *G. fascicularis* from Sanya, suggesting *Symbiodinium* communities in *G. fascicularis* from Sansha were relatively stable during the two months. The high dissimilarities between *Symbiodinium* communities from Hong Kong and Sansha/Sanya due to temperature could result from either spatial or temporal variations, as averaged SST in Hong Kong rose ~2 °C/~10 °C from March to April (Sanya sampling)/June (Sansha sampling), respectively. It’s possible that if Hong Kong and Sanya samplings are both finished in June, there would be less differences between *Symbiodinium* communities in *G. fascicularis* from Hong Kong and Sansha, and *Symbiodinium* communities in *G. fascicularis* from Sanya and Sansha would be more similar. However, spatial variation still played a more important role in the present study: *Symbiodinium* communities in *G. fascicularis* from Sanya and Sansha were highly similar even Sansha sampling was two months late; while *Symbiodinium* communities in *G. fascicularis* from Hong Kong and Sanya were quite different even sampled at the similar dates. The results of the relationships between *Symbiodinium* communities and environmental conditions would not be affected by sampling at different time points as long as the environmental data can well reflect the environmental conditions at the sampling points.

### Potential roles of *Symbiodinium* in the coral host in response to bleaching and future climate change

Our results support an argument of coevolution between the coral host and *Symbiodinium* because *Symbiodinium* from congeneric corals demonstrated closer phylogenetic relationships and a geographic specificity was observed in the *Symbiodinium* phylogeny. While we found three C subclades have closer phylogenetic relationships with D subclades than other C subclades, it might result from classification errors, as perfect matches of C2r and C163b were not found in NCBI database and the perfect match of C161 was not identified as *Symbiodinium* C clade in NCBI. *Symbiodinium* clade C is believed to possess a higher photosynthetic efficiency, whereas *Symbiodinium* clade D is more heat tolerant^33^. Therefore, it can be assumed that the *Symbiodinium* may contribute to the host adaptation and survival in response to fluctuating environmental conditions. Dixon *et al*.^34^ found that *Acropora millepora* larvae derived from warmer locations possessed greater thermal tolerance, indicating that thermal tolerance was heritable^34^. Cooper *et al*.^11^ observed that adult *A. millepora* acquired a greater number of heat-tolerant *Symbiodinium* in response to higher temperatures, suggesting that a potentially thermal-tolerant coral would prefer thermal-tolerant *Symbiodinium* subclades during growth to facilitate their future survival during exposure to relatively high temperatures. However, Palumbi *et al*.^35^ found that *A. hyacinthus* became more heat tolerant without changes in symbiont clade composition as well as gene expression^35^. Thus, different coral species may possess different heat tolerance mechanisms.

Tchernov *et al*.^36^ showed that a rise in temperature damaged the membranes of free-living *Symbiodinium* and thus disrupted their photosynthetic ability, ultimately leading to death, and that the compositions of membranes determined the thermal stress sensitivity of *Symbiodinium*^36^. In the present study, we found that as the temperature rose across the regions, the relative abundance of some heat-sensitive *Symbiodinium* C subclades, such as C116, C17, decreased in seawater. In contrast, some *Symbiodinium* that are supposed to be heat sensitive according to the clade characteristics, such as C31, were not greatly affected much across the regions and C31 was found to have a high relative abundance in *Montipora* spp. from Sanya. These findings indicated that some *Symbiodinium* might possess compensation mechanisms to survive under thermal stress that could be taken advantage by the coral host to adapt to future climate change. However, previous studies have shown that some coral species acquired their *Symbiodinium*, e.g., C31, through vertical transmission^37^,^38^. In the present study, we also detected C15h, C21a, C35 and C26 in coral samples but not in ambient seawater samples in certain locations, either due to a potential vertical transmission or to an insufficient sensitivity of the current techniques. We suspect that if a vertically transmitted *Symbiodinium* fails to develop compensation mechanisms, coral host would become more susceptible to future climate change.

*G. fascicularis* was found to have greater heat tolerance than *Montipora* sp.^39^, and similarly in our study, very few members of the *Symbiodinium* D clade were detected among the *Montipora* spp., even in Sanya, where temperature is relatively high. This observation raises the possibility that heat-tolerant *Symbiodinium* facilitates coral adaptation. In response to global warming with a predicted increase in bleaching events, coral species that can acquire more heat-tolerant *Symbiodinium* will survive better. Coral species that lack the symbiotic flexibility with *Symbiodinium* subclades have also been found to be more susceptible to bleaching^29^. However, some heat-tolerant *Symbiodinium* can reduce host calcification and photosynthetic efficiency, which may impair the competitive ability of the coral^26^,^33^. While Cunning *et al*.^40^ demonstrated that the growth disadvantage of heat-tolerant *Symbiodinium* could be reduced by elevated temperature, thus enhancing the survival of coral host. More investigations are needed to decipher the mechanisms that how coral species with different *Symbiodinium* survive during future climate change.

Pettay *et al*.^33^ found that *Symbiodinium trenchii* invaded the Caribbean and noted that this *Symbiodinium* might drive coral-algal symbiosis dynamics during climate change, which is inconsistent with our findings. According to the present study, all *Symbiodinium* (including *Symbiodinium trenchii*) could potentially be transmitted between corals and surrounding seawater, and certain subclades of *Symbiodinium* were enriched in the coral host. For certain *Symbiodinium* subclades such as C161, congeneric corals in different regions surrounded by a similar relative abundance of certain *Symbiodinium* are internally enriched with different relative abundances of that *Symbiodinium*. Therefore, the acquisition of *Symbiodinium* by corals shall not be a random but a selective event. Likely, corals are capable of selecting certain *Symbiodinium* that are potentially more beneficial to them, and the selected *Symbiodinium* facilitate the adaptation of the host. Thus, the coral host drives the dynamics of the symbiosis. However, we cannot exclude the possibility that, in certain coral species, invading *Symbiodinium* may drive the dynamics of the symbiosis.

### Ecological implications

The adaptation of the coral reef ecosystem to future climate change strongly relies on the adaptation of their internal *Symbiodinium* community. In the South China Sea, among all potential environmental factors, temperature can serve as a major factor for shaping coral-algal symbiosis. Therefore, a rise in SST is a prominent threat to the South China Sea corals, but still corals have the potential to adapt to future climate change with their flexibility of symbiosis with *Symbiodinium*. Although more caution should be adhered to when transplanting a new species into a region^33^, the introduction of more heat-tolerant *Symbiodinium* into the coral reef ecosystem may facilitate their adaptation to the rising SST^41^.

Previous studies have shown that heat-tolerant corals contain a greater number of clade D *Symbiodinium*^42^. Although both *G. fascicularis* and *Montipora* spp. have the potential to change their *Symbiodinium* community structures in response to environmental changes, *G. fascicularis* appears to prefer more heat-tolerant *Symbiodinium* subclades, which may lead to its dominance during future climate change. Indeed, in recent years some dominant coral species have been declining while some subdominant coral species are becoming more dominant^29^,^43^. During the development of temperature-driven symbiosis, the flexibility of coral-algal symbiosis and preference for certain *Symbiodinium* of coral species can directly determine coral species’ competition during future climate change, which will ultimately affect future coral population structures.

However, human activities such as seawater pollution, overfishing, and tourism etc. have also caused a rapid decline in the coral cover^44^,^45^. In the present study, nutrients had only a marginal impact on coral-algal symbiosis, considering nutrients contribute the most to coral cover and species richness loss compared with other water quality parameters^46^, suggesting a lack of symbiotic flexibility in response to seawater pollution such as eutrophication. Increases in coastal nutrients are frequently linked to human activities^47^,^48^, which suggests that human activities can also affect coral-algal symbiosis. This observation indicates that seawater pollution may be another important threat to the coral reef.

## Conclusions

The present study demonstrated that temperature drove coral-algal symbiosis spatial change in the South China Sea and corals had the potential to adapt to future climate change with selecting more heat-tolerant *Symbiodinium* under gradually rising SST. Thus, for the future conservation of corals in the South China Sea, the introduction to coral hosts of heat-tolerant *Symbiodinium* might facilitate their adaptation to future climate change. Despite the high level of spatial flexibility of coral-algal symbiosis in *G. fascicularis* and *Montipora* spp. in the South China Sea, future studies are needed on a larger geographic scale and shall be expanded to other dominant coral species like *Acropora* spp. to further address coral-algal symbiosis in response to future climate change.

## Methods

### Sample collection and preparation

Forty-eight coral samples of *G. fascicularis* (n = 24 colonies) and *Montipora* spp. (n = 24 colonies) and 10 seawater samples in total were collected from two Hong Kong sampling sites in March 2014, two Sanya sampling sites in April 2014 and one Sansha sampling site in June 2014 (Table 1). At each sampling site, one small piece (~1 cm × 1 cm) from each healthy coral colony was collected using a hammer/chisel set and wrapped in a tagged bag filled with seawater. On board, the coral pieces were immediately washed with filtered seawater, fixed in 70% ethanol, stored and transported to the laboratory using a cool box containing dry ice. The seawater surrounding the coral colonies was also collected as controls. Free-living *Symbiodinium* cells in seawater were collected by filtering seawater through 0.22-μm polycarbonate membranes and fixed with 50% ethanol. All of the fixed coral and seawater samples were stored at −30 °C until DNA extraction.

### Environmental data collection

Hong Kong environmental data were provided by the Hong Kong Environmental Protection Department (HKEPD). Data for the sampling month from five selected monitoring stations around each sampling site (Table S1, Table S2, Supporting Information) were collected and averaged, and the averages were used as environmental factor data for a given sampling site. For Sanya and Sansha, the seawater temperature, salinity and depth were measured during the sampling *in situ* with CTD (Idronaut, Italy), while dissolved oxygen (DO) was assessed *in situ* using a YSI 6600V2-02 multi-parameter instrument (YSI, USA). The seawater samples were collected and immediately filtered (Whatman GF/F, 47 mm) to analyze dissolved nutrients (nitrite, nitrate, ammonia and phosphate) using a Lachat QC8500 Flow Injection Autoanalyzer (Lachat Instruments, USA).

### DNA extraction and amplicon sequencing

A small fragment (~0.5 cm × 0.5 cm) cut from each fixed piece of coral was first rinsed with 1*PBSE (137 mM NaCl, 2.7 mM KCl, 4.3 mM Na_2_HPO_4_·7H_2_O, 1.4 mM KH_2_PO_4_ and 10 mM EDTA) and then mashed in 1*PBSE using a mortar and a pestle. The mashed coral serous was centrifuged at 12000 g, and the pellets were collected for DNA extraction. DNA was extracted from each pellet using the FastDNA^®^ Spin Kit for Soil (MP Biomedicals, France) following the protocol provided with the kit. After quality and purity examinations, the extracted DNA samples were applied as PCR templates. The *Symbiodinium* ITS2 region of the rDNA was amplified by PCR using primers (F: 5′GAATTGCAGAACTCCGTG-3′; R: 5′ GGATCCATATGCTTAAGTTCAGCGGGT-3′) designed to produce 330–360 bp ITS2 fragments^49^. At the 5′ terminus of the forward primer, six-nucleotide unique barcodes were attached for multiplexed sequencing. The PCR amplifications were conducted in a 50-μL reaction volume containing ~50 ng of DNA, 25 μL of 2× Taq Platinum PCR Master (Tiangen, China), 200 nM of each primer, and ddH_2_O up to the final volume. The reactions were performed under the following conditions: 94 °C for 5 min, followed by 35 cycles of 94 °C for 30 s, 51 °C for 30 s, 72 °C for 30 s, and a final extension at 72 °C for 5 min. Each sample was amplified in three independent reactions to minimize potential PCR bias, and then purified using the PureLink^®^ PCR Purification Kit (Invitrogen, USA). The purified PCR products were quantified using a Thermo NanoDrop 2000 UV-Vis Spectrophotometer and then mixed based on equal mass for subsequent multiplexed amplicon sequencing. The final DNA samples were sequenced following a paired-end (PE) 300 bp × 2 strategy on an Illumina MiSeq sequencer operated by the Novogene company (Beijing, China). The sequencing datasets were submitted to the NCBI Sequence Read Archive under accession number SRP066283.

### Data processing, ITS2 database establishment and data analysis

Strict quality control and sequence filtration were applied to ensure the accuracy of the following analysis. Adaptors, short reads, and low quality reads were first removed by the sequencing company. The PEAR (paired-end read merger) tool was applied to obtain full-length ITS2 rDNA fragments with merging overlapping PE reads to generate ITS2 sequences^50^ (http://sco.h-its.org/exelixis/web/software/pear/, see commands in Supporting Information). ITS2 tags were demultiplexed into all samples in the QIIME platform^51^ by identifying unique barcodes (http://qiime.org/index.html, see commands in Supporting Information). We found that the previous ITS2 database included some duplicate sequences^52^, therefore we uploaded the previous ITS2 database to CD-HIT Suite website (http://weizhongli-lab.org/cdhit_suite/cgi-bin/index.cgi?cmd=cd-hit-est), set sequence identity cut-off as 100%, compared both strands, set all other parameters as default^53^, removed the duplicates, merged the annotations and used the results to establish a non-redundant ITS2 database (ITS2 Database.doc, Supporting Information). Next, all of the sequences were aligned to the ITS2 database by BLASTN^54^ based on E-value of 1e-5 (see commands in Supporting Information), the output were then filtered in Microsoft Excel (Microsoft Corp., USA) based on 97% similarity for *Symbiodinium* subclade identification. For ITS2 rDNA, 97% sequence similarity has been proved to be reliable for *Symbiodinium* subclade classification and could permit differences of ten base pairs for identification within the same *Symbiodinium* subclade as a consequence of intra-genomic sequence divergence^10^.

Thirteen (sub)dominated *Symbiodinium* subclades were picked from 131 total aligned *Symbiodinium* subclades for following analysis. The relative abundances of picked *Symbiodinium* subclades were >10% in at least one sample or their average relative abundances in all samples were at least around 1% and the filtering results can indicate the original communities well (whole_community_data.xls, Supporting Information). To profile *Symbiodinium* communities in different samples, a heat map and scatter plots were created with OriginPro 2015 (OriginLab, USA). To justify significances between *Symbiodinium* community structures across the sampling sites, a two-way ANOSIM and a permutational multivariate analysis of variance (PERMANOVA) were performed using PRIMER 7 software^55^. To present the relationships of the *Symbiodinium* community structures from different sampling sites, a nMDS ordination as well as clustering analysis were developed based on the square root-transformed Bray-Curtis similarity matrix of *Symbiodinium* profiles (*Symbiodinium* relative abundance) in PRIMER. To test the relationships among environmental factors, *Symbiodinium* community, and sampling sites, CCA was conducted in Canoco 5^56^. CCA uses ecological data sets to extract synthetic environmental gradients which are the basis for revealing the differential habitat preferences of microbes and has been proved to be reliable when combining spatial and seasonal sampling together, as long as the sample size is granted and the environment conditions can reflect habitat preference of the communities^57^,^58^. To identify the phylogenetic relationships among the dominant *Symbiodinium* subclades found in this study, two phylogenetic trees were constructed based on the Kimura 2-parameter model with uniform rates among sites using Maximum Likelihood in MEGA 6^59^ and Bayesian Inference in Mrbayes^60^, respectively.

## Additional Information

**How to cite this article**: Tong, H. *et al*. Temperature shapes coral-algal symbiosis in the South China Sea. *Sci. Rep.* **7**, 40118; doi: 10.1038/srep40118 (2017).

**Publisher's note:** Springer Nature remains neutral with regard to jurisdictional claims in published maps and institutional affiliations.

## Supplementary Material

Supplementary Materials

Whole community data

ITS2 database

## Figures and Tables

**Figure 1:**
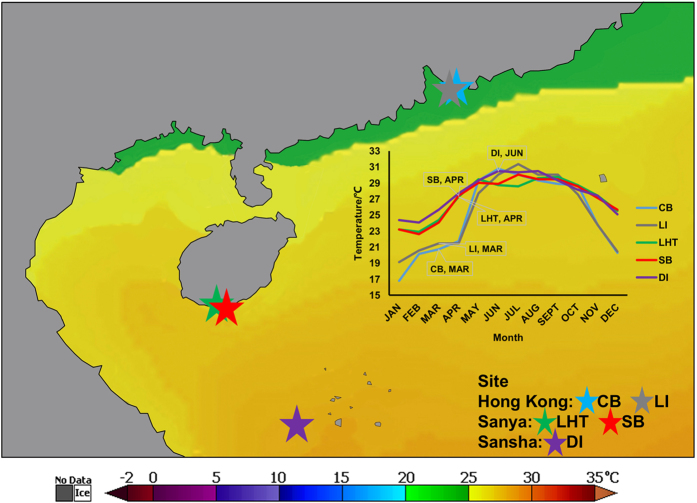
Sampling sites in the South China Sea: Crescent Bay (CB, Hong Kong), Lamma Island (LI, Hong Kong), Luhuitou (LHT, Sanya), Sunny Bay (SB, Sanya) and Drummond Island (DI, Sansha). The plot displays mean SST for each sampling site in 2014 (NASA Earth Observations) and the labels indicate sampling month for each site. The temperature heatmap was generated on the NOAA Satellite and Information Service website (http://coralreefwatch.noaa.gov/satellite/bleaching5km/index_5km_sst.php) and using NOAA Coral Reef Watch Virtual Stations SST data for 2014 (NOAA Coral Reef Watch. 2014, updated daily. *NOAA Coral Reef Watch Annual 5-km Satellite Sea Surface Temperature Product for Coral Triangle*, Jan. 1, 2014–Dec. 31, 2014. College Park, Maryland, USA: NOAA Coral Reef Watch. Data set accessed at http://coralreefwatch.noaa.gov/satellite/bleaching5km/index_5km_sst.php) shows the thermal gradient in the South China Sea.

**Table 1:** Sampling sites, sampling dates and sample ID abbreviations for *G. fascicularis* and *Montipora* spp.

Regions	Climate	Sites	Coordinates	Dates (yyyy-mm-dd)	*G. fascicularis*	*Montipora* spp.
Hong Kong	Subtropical	Crescent Bay (CB)	E114.314°, N22.531°	2014-03-24	*G. fascicularis* (GA)	*M. peltiformis* (MO)
		Lamma Island (LI)	E114.135°, N22.187°	2014-03-19	N.A.	*M. venosa* (MO)
Sanya	Tropical	Luhuitou (LHT)	E109.471°, N18.212°	2014-04-04	*G. fascicularis* (GA)	*M. monasteriata* (MO)
		Sunny Bay (SB)	E109.610°, N18.199°	2014-04-03	*G. fascicularis* (GA)	*M. monasteriata* (MO)
Sansha	Tropical	Drummond Island (DI)	E111.778°, N16.523°	2014-06-19	*G. fascicularis* (GA)	N.A.
