# Supplementary material for: Temperature shapes coral-algal symbiosis in the South China Sea
Source: Sci Rep. 2017 Jan 13;7:40118. doi: 10.1038/srep40118 (PMC5234030; doi:10.1038/srep40118)
Supplement: Supplementary Materials [file srep40118-s1.doc]

**Temperature shapes coral-algal symbiosis in the South China Sea**

Haoya Tong ^1^, Lin Cai^1^, Guowei Zhou^1,2,3^, Tao Yuan^2^, Weipeng Zhang^1^, Renmao Tian^1^, Hui Huang^2,3*^, Pei-Yuan Qian ^1^, *

^1^HKUST Shenzhen Research Institute and Division of Life Science, Hong Kong University of Science and Technology, Clear Water Bay, Hong Kong SAR, China

^2^ Key Laboratory of Tropical Marine Bio-resources and Ecology, South China Sea Institute of Oceanology, Chinese Academy of Sciences, China

**^*^Co-corresponding author:**

**Hui Huang**, PhD

South China Sea Institute of Oceanology, Chinese Academy of Sciences, 164 West Xingang Road, Guangzhou, China

Phone & Fax: +862-8446-0294

E-mail: [huanghui@scsio.ac.cn](mailto:huanghui@scsio.ac.cn)

**Pei-Yuan Qian**, PhD

Shenzhen Research Institute and Division of Life Science, Hong Kong University of Science and Technology, Hong Kong SAR, China

Phone: +852-2358-7331

Fax: +852-2358-1559

E-mail: [boqianpy@ust.hk](mailto:boqianpy@ust.hk)

**Supplementary Materials**

**Table S1**. Details regarding the Hong Kong Environmental Protection Department (HKEPD) water quality monitoring stations.

| **Sampling sites** | **HKEPD monitoring date** | **HKEPD monitoring station numbers** | **Coordinates** |
| --- | --- | --- | --- |
| CB | 2014/03/17 | MM3 | E114 ^o^ 18.615’, N22 ^o^ 33.714’ |
|  |  | MM4 | E114 ^o^ 21.483’, N22 ^o^ 33.817’ |
|  |  | MM5 | E114 ^o^ 23.633’, N22 ^o^ 31.233’ |
|  |  | MM7 | E114 ^o^ 17.824’, N22 ^o^ 31.409’ |
|  |  | MM17 | E114 ^o^ 20.960’, N22 ^o^ 30.192’ |
| LI | 2014/03/17 | SM3 | E114 ^o^ 8.980’, N22 ^o^ 13.527’ |
|  |  | SM5 | E114 ^o^ 6.728’, N22 ^o^ 12.141’ |
|  |  | SM6 | E114 ^o^ 4.743’, N22 ^o^ 11.500’ |
|  |  | SM18 | E114 ^o^ 4.746’, N22 ^o^ 9.211’ |
|  |  | SM19 | E114 ^o^ 13.077’, N22 ^o^ 9.211’ |

**Table S2.** Environmental data for five sampling sites.

| Regions | Sampling sites | Locations | Temperature (°C) | Depth  (m) | | Salinity | | DO (mg/L) | NO_3_^-^(µg/L) | NO_2_^-^(µg/L) | NH_4_^+^ (µg/L) | PO_4_^3-^(µg/L) |
| --- | --- | --- | --- | --- | --- | --- | --- | --- | --- | --- | --- | --- |
| Hong Kong | CB | E114.314^o^, N22.531^o^ | 16.74 | 4.10 | 33.14 | | 6.44 | | 21.40 | 7.40 | 21.00 | 7.20 |
|  | LI | E114.135^o^, N22.187^o^ | 16.56 | 2.94 | 33.14 | | 6.60 | | 108.00 | 18.20 | 26.40 | 14.00 |
| Sanya | SB | E109.610^o^, N18.199^o^ | 26.83 | 1.63 | 33.87 | | 6.53 | | 19.54 | 5.28 | 9.66 | 2.48 |
|  | LHT | E109.471^o^, N18.212^o^ | 25.89 | 0.98 | 33.62 | | 5.25 | | 27.40 | 11.97 | 22.88 | 8.85 |
| Sansha | DI | E111.778^o^, N16.523^o^ | 30.41 | 6.03 | 33.12 | | 6.13 | | 22.97 | 3.75 | 10.53 | 2.37 |

Fig. S1 Complete linkage cluster analysis of *G. fascicularis* and *Montipora* spp. with dominant *Symbiodinium* subclade community compositions based on the square root-transformed relative abundance matrix by the Bray-Curtis measure of dissimilarity. Eight numbers represent eight groups in Fig. 4 (A) that are cut based on 60% similarity.

Fig. S2 Relationships among selected environmental factors, *Symbiodinium* subclades, and sampling sites. CCA indicates the relationship among environmental factors, *Symbiodinium* subclades, and coral *Symbiodinium* community compositions from different sampling sites. (A): CCA of *G. fascicularis* samples. The first axis (CCA Axis 1) explains 75.88% of the total variation and 92.71% of the fitted variation; the second axis (CCA Axis 2) explains 5.96% of the total variation and 7.28% of the fitted variation. (B): CCA of *Montipora* spp. samples. CCA Axis 1 explains 31.72% of the total variation and 60.18% of the fitted variation; CCA Axis 2 explains 20.89% of the total variation and 39.63% of the fitted variation.

**Commands:**

**PEAR commands:**

pear-0.9.5-64 -f forward_read_file -r reverse_read_file -o output_file -m 500 -n 400 -q 30 -p 0.001 -y 5G -j 5

**QIIME commands:**

Extract barcodes:

extract_barcodes.py -f input_file.fastq -l 6 -c barcode_single_end

Split libraries:

split_libraries_fastq.py -i reads.fastq -b barcodes.fastq -m sample_mapping.xls -q 20 -o output --barcode_type 6 -n 1 -r 2 -s 1 --max_barcode_errors 0

**BLAST commands:**

Format database:

makeblastdb -in Symbio_nr.fasta -dbtype nucl -parse_seqids -out Symbiodinium_database

Conduct Blastn:

blastn –query input_file –out output_file–db Symbiodinium_database –outfmt 6 –evalue 1e-5 –num_threads 4 –max_target_seqs 1
