# Supplementary material for: Temperature shapes coral-algal symbiosis in the South China Sea
Source: Sci Rep. 2017 Jan 13;7:40118. doi: 10.1038/srep40118 (PMC5234030; doi:10.1038/srep40118)
Supplement: ITS2 database [file srep40118-s3.doc]

| >GS_A1 |
| --- |
| AACCAATGGCCTCTTGAACGTGCATTGCGCTCTTGGGATATGCCTGAGAGCATGTCTGCT |
| TCAGTGCTTCTACTTTCATTTTCTGCTGCTCTTGTTATCAGGAGCAGTGTTGCTGCATGC |
| TTCTGCAAGTGGCACTGGCATGCTAAATATCAAGTTTTGCTTGCTGTTGTGACTGATCAA |
| CATCTCATGTCGTTTCAGTTGGCGAAACAAAAGCTCATGTGTGTTCTTAACACTTCCTAG |
| CATGAAGTCAGACAA |
| >GS_A1.1=A13 |
| AACCAATGGCCTCTTGAACGTGCATTGCGCTCTTGGGATATGCCTGAGAGCATGTCTGCT |
| TCAGTGCTTCTACTTTCATCTTCTGCTGCTCTTGTTATCAGGAGCAGTGTTGCTGCATGC |
| TTCTGCAAGTGGCACTGGCATGCTAAATATCAAGTTTTGCTTGCTGTTGTGACTGATCAA |
| CATCTCATGTCGTTTCAGTTGGCGAAACAAAAGCTCATGTGTGTTCTTAACACTTCCTAG |
| CATGAAGTCAGACAA |
| >GS_A1.2 |
| AACCAATGGCCTCTTGAACGTGCATTGCGCTCTTGGGATATGCCTGAGAGCATGTCTGCT |
| TCAGTGCTTCTACTTTCATTTTCTGCTGCTCTTGTTATCAGGAGCAGTGTTGCTGCATGC |
| TTCTGCAAGTGGCACTGGCATGCTGAATATCAAGTTTTGCTTGCTGTTGTGACTGATCAA |
| CATCTCATGTCGTTTCAGTTGGCGAAACAAAAGCTCATGTGTGTTCTTAACACTTCCTAG |
| CATGAAGTCAGACAA |
| >ST_A1a |
| AACCAATGGCCTCTTGAACGTGCATTGCGCTCTTGGGATATGCCTGAGAGCATGTCTGCTTCAGTGCTTCTACTTTCATTTTCTGCTGCTCTTGTTATCAGGAGCAGTGTTGCTGCATGCTTCTACAAGTGGCACTGGCATGCTAAATATCAAGTTTTGCTTGCTGTTGTGACTGATCAACATCTCATGTCGTTTCAGTTGGCGAAACAAAAGCTCATGTGTGTTCTTAACACTTCCTAGCATGAAGTCAGACAA |
| >ST_STR1 |
| AACCAATGGCCTCTTGAACGTGCATTGCGCTCTTGGGATATGCCTGAGAGCATGTCTGCTTCAGTGCTTCTACTTTCATTTTCTGCTGGTCTTGTTATCAGGAGCAGTGTTGCTGCATGCTTCTGCAAGTGGCACTGGCATGCTAAATATCAAGTTTTGCTTGCTGTTGTGACTGATCAACATCTCATGTCGTTTCAGTTGGCGAAACAAAAGCTCATGT |
| >LJ_A1c |
| GTCTGACTTCATGCTAGGAAGTGTTAAGAACACACATGAGCTTTTGTTTCGCCAACTGAAACGACATGAGATGTTGATCAGTCATAACAGCAAGCAAAACTTGATATTTAGCATGCCAGTGCCACTTGCAGAAGCATGCAGCAACACTGCTCCTGATAACAAGAGCAGCAGAAAATGAAAGTAGAAGCACTGAAGCAGACATGCTCTCAGGCATATCCCAAGAGCGCAATGCACGTTCAAGAGGCCATTGGTT |
| >ST_A1med |
| AACCAATGGCCTCTTGAACGTGCATTGCGCTCTTGGGATATGCCTGAGAGCATGTCTGCTTCAGTGCTTCTTATATCCACTTGTTGCTGCTGCTCTTTCGTTAAAGGGCAGCGTGGGTGCATGCTACTGCTTTGCAGCACTGGCATGCGTAGATTTGCTGCTTGCTCACTGGTTCGATTGATCACTTCATCAAATCTTGTCAGTGTTGCAAGTCTTGCGTATGTGTTTTGACACATCCTAGCATGAAGTCAGACAA |
| >GS_A2 |
| AACCAATGGCCTCTTGAACGTGCATTGCGCTCTTGGGATATGCCTGAGAGCATGTCTGCT |
| TCAGTGCTTCTAACTCTCTATGTGTCCTGCTGCTCTCTCCCTTTGAGAGCAGTGCTGCTG |
| CATGCTACTGCGCTGCTGCACTGGCATGCTCAATATCAAGTTTTGCCCACTGGTTTGACT |
| TGATCATCATCTTGTCTTGCAGATTGGGTAAAATAGGAAATAGTGTTTGCAACACTTCCT |
| AGCATGAAGTCAGACAA |
| >GS_A3 |
| AACCAATGGCCTCTTGAACGTGCATTGCGCTCTTGGGATATGCCTGAGAGCATGTCTGCT |
| TCAGTGCTTCTACTTTCTTTTCTGCTGCTCTTGTTATCAGGAGCAGTGCTGCTGCATGCT |
| TCTGCAATTGGCACTGGCATGCTAAGTACCAAGTTTCGCTTGCTGTTGTGACTGATCAAC |
| ATCTCATGTCGTTTCAGTTGGCGAAACAAAGGCTTGTGTGTTCCAACACTTCCTAGCATG |
| AAGTCAGACAA |
| >ST_A3a_TD1E |
| AACCAATGGCCTCTTGAACGTGCATTGCGCTCTTGGGATATGCCTGAGAGCATGTCTGCTTCAGTGCTTCTACTTTCTTTTCTGCTGCTCTTGTTATCAGGAGCAGTGCTGCTGCATGCTTCTGCAATTGGCACTGGCATGCTAAGTACCAAGTTTCGCTTGCTGTTGTGACTGATCAACATCTCATGTCGTTTCAGTTGGCAAAACAAAGGCTTGTGTGTTCCAACACTTCCTAGCATGAAGTCAGACAA |
| >GS_A3b |
| AACCAATGGCCTCTTGAACGTGCATTGCGCTCTTGGGATATGCCTGAGAGCATGTCTGCT |
| TCAGTGCTTCTACTTTCTTTTCTGCTGCTCTTGTTATCAGGAGCAGTGCTGCTGCATGCT |
| TCTGCAATTGGCACTGGCATGCTAAGTACCAAGTTTTGCTTGCTGTTGTGACTGATCAAC |
| ATCTCATGTCGTTTCAGTTGGCGAAACAAAGGCTTGTGTGTTCCAACACTTCCTAGCATG |
| AAGTCAGACAA |
| >GS_A4 |
| AACCAATGGCCTCTTGAACGTGCATTGCGCTCTTGGGATATGCCTGAGAGCATGTCTGCT |
| TCAGTGCTTCTACTTTCATTTTCTGCTGCTCTTGTTATCGGGAGCAGTGTTGCTGCATGC |
| TTCTGCAAAAGGCACTGGCATGCTAAGTATCAAGTTTTGCTTGCTGTTCTGACTGATCAA |
| CATCTCATGTCGTTTCAGTTGGCGAAACAAAAGCTGAAGTGTGTTCTTAACACTTCCTAG |
| CATGAAGTCAGACAA |
| >GS_A4a |
| AACCAATGGCCTCTTGAACGTGCATTGCGCTCTTGGGATATGCCTGAGAGCATGTCTGCT |
| TCAGTGCTTCTACTTTCATTTGCTGCTGCTCTTGTTATCGGGAGCAGTGTTGCTGCATGC |
| TTCTGCAAAAGGCACTGGCATGCTAAGTATCAAGTTTTGCTTGCTGTTCTGACTGATCAA |
| CATCTCATGTCGTTTCAGTTGGCGAAACAAAAGCTGAAGTGTGTTCTTAACACTTCCTAG |
| CATGAAGTCAGACAA |
| >GS_A4.1 |
| AACCAATGGCCTCTTGAACGTGCATTGCGCTCTTGGGATGCCTGAGAGCATGTCTGCTTC |
| AGTGCTTCTACTTTCATTTTCTGCTGCTCTTGTTATCGGGAGCAGTGTTGCTGCATGCTT |
| CTGCAAAAGGCACTGGCATGCTAAGTATCAAGTTTTGCTTGCTGTTCTGACTGATCAACA |
| TCTCATGTCGTTTCAGTTGGCGAAACAAAAGCTGAAGTGTGTTCTTAACACTTCCTAGCA |
| TGAAGTCAGACAA |
| >GS_A4.2 |
| AACCAATGGCCTCTTGAACGTGCGCTCTTGGGATATGCCTGAGAGCATGTCTGCTTCAGT |
| GCTTCTACTTTCATTTTCTGCTGCTCTTGTTATCGGGAGCAGTGTTGCTGCATGCTTCTG |
| CAAAAGGCACTGGCATGCTAAGTATCAAGTTTTGCTTGCTGTTCTGACTGATCAACATCT |
| CATGTCGTTTCAGTTGGCGAAACAAAAGCTGAAGTGTGTTCTTAACACTTCCTAGCATGA |
| AGTCAGACAA |
| >GS_A4.3 |
| AACCAATGGCCTCTTGAACGTGCATTGCGCTCTTGGGATATGCCTGACAGCATGTCTGCT |
| TCAGTGCTTCTACTTTCATTTTCTGCTGCTCTTGTTATCGGGAGCAGTGTTGCTGCATGC |
| TTCTGCAAAAGGCACTGGCATGCTAAGTATCAAGTTTTGCTTGCTGTTCTGACTGATCAA |
| CATCTCATGTCGTTTCAGTTGGCGAAACAAAAAAGCTGAAGTGTGTTCTTAACACTTCCT |
| AGCATGAATTCAGACAA |
| >GS_A5 |
| AACCAATGGCCTCTTGAACGTGCATTGCGCTCTTGGGATATGCCTGAGAGCATGTCTGCT |
| TCAGTGCTTCTACTTTCATTTTCTGCTGCTCTTGTTATCAGGAGCAGTGCTGCTGCATGC |
| TTCTGCAATGGGCACTGGCATGCTAAGTACCAAGTTTTGCTTGCTGTTGTGACTGATCAA |
| CATCTCATGTCGTTTCAGTTGGCGAAACAAAGGCTTGTGTGTTCCAACACTTCCTAGCAT |
| GAAGTCAGACAA |
| >GS_A6 |
| AACCAATGGCCTCTTGAACGTGCATTGCGCTCTTGGGATATGCCTGAGAGCATGTCTGCT |
| TCAGTGCTTCTACTTTCTTTTCTGCTGCTCTTGTTATCAGGAGCAGTGCTGCTGCATGCT |
| TCTGCAATTGGCACTGGCATGCTAAGTACCAAGTTTCGCTCGCTGTTGTGACTGATCAAC |
| ATCTCATGTCGTTTCAGTTGGCGAAACAAAGGCTTGTGTGTTCCAACACTTCCTAGCATG |
| AAGTCAGACAA |
| >GS_A7 |
| AACCAATGGCCTCTTGAACGTGCATTGCGCTCTTGGGATATGCCTGAGAGCATGTCTGCT |
| TCAGTGCTTCTACTTTCTTTTCTGCTGCTCTTGTTATCATGAGCAGTGCTGCTGCATGCT |
| TCTGCAATTGGCACTGGCATGCTAAATACCAAGTTTTGCTTGCTGTTGTGACTGATCAAC |
| ATCTCATGTCGTTTCAGTTGGCGAAACAAAGGCTTGTGTGTTCCAACACTTCCTAGCATG |
| AAGTCAGATAA |
| >GS_A8 |
| AACCAATGGCCTCTTGAACGTGCATTGCGCTCTTGAGATATGCCTGAGAGCATGTCTGCT |
| TCAGTGCTTCTACTTTCTTTTCTGCTGCTCTTGTTATCATGAGCAGTGCTGCTGCATGCT |
| TCTGCAATTGGCACTGGCATGCTAAGTATCAAGTTTTGCTTGCTGTTGTGACTGATCAAC |
| ATCTCATGTCGTTTCAGTTGGCGAAACAAAGGCTTGTGTGTTCCAACACTTCCTAGCATG |
| AAGTCAGATAA |
| >GS_A9 |
| AACCAATGGCCTCTTGAACGTGCATTGTGCTCTTGGGATATGCCTGAGAGCATGTCTGCT |
| TCAGTGCTTCTACTTTCATTTTCTGCTGGTCTTGTTATCAGGAGCAGTGTTGCTGCGTGC |
| TTCTGCAAGTGGCACTGGCATGCTAAATATCAAGTTTTGCTTGCTGTTGTGACTGATCAA |
| CATCTCATGTCGTTTCAGTTGGCGAAACAAAAGCTCATGTGTGTTCTTAACACTTCCTAG |
| CATGAAGTCAGACAA |
| >GS_A10 |
| AACCAATGGCCTCTTGAACGTGCATTGCGCTCTTGGGATATGCCTGAGAGCATGTCTGCT |
| TCAGTGCTTCTTCTTTCATTTTCTGCTGCTCTTGTTATCCGGAGCAGTGTTGCTGCAAGC |
| TTCTGCAAGTGGCACTGGCATGCTAAATATCGTTTTGCTTGCTGTTGTGACTGATCAACA |
| TCTCATGTCGTTTCAGTTGGCGAAACAAAAGCTCATGTGTGTTCTTAACACTTCCTAGCA |
| TGAAGTCAGACAA |
| >GS_A11 |
| AACCAATGGCCTCTTGAACGTGCATTGCGCTCTTGGGATATGCCTGAGAGCATGTCTGCT |
| TCAGTGCTTCTACTATCTTTTCTGCTGCTCTTGTTGTCAGGAGCAGTGCTGCTGCATGCT |
| TCTGCAATTGGCACTGGCATGCTAAGTACCAAGTTTCGCTTGCTGTTGTGACAGATCAAC |
| ATCTCATGTCGTTTCAGTTGATGAAACAAGGCTTGCGTGTTCCAACACTTCCTAGCATGA |
| AGTCAGACAA |
| >GS_A12 |
| AACCAATGGCCTCTTGAACGTGTATTGCGCTCTTGGGATATGCCTGAGAGCATGTCTGCT |
| TCAGTGCTTCTACTTTCATTTTCTGCTGCTCTTGCTATCAGGAGCAGTGTTGCTGCATGC |
| TTCTGCAAGCGGCACTGGCATGCTAAATATCAAGTTTTGCTTGCTGTTGTGACTGATCAA |
| CATCTCATGTCGTTTCAGTTGGCGAAACAAAAGCTCATGTGTGTTCTTAACACTTCCTAG |
| CATGAAGTCAGACAA |
| >LJ_A14 |
| TTGTCTGACTTCATGCTAGGAAGTGTTAAGAACACACTTCAGCTTTTGTTTCGCCAACTGAGACGACATGAGATGTTGATCAGTCAGAACAGCAAGCAAAACTTGATACTTAGCATGCCAGTGCCTTTTGCAGAAGCATGCAGCAACACTGCTCCCGATAACAAGAGCAGCAGAGAATGAAAGTAGAAGCACTGAAGCAGACATGCTCTCAGGCATATCCCAAGAGCGCAATGCACGTTCAAGAGGCCATTGGTTCA |
| >GS_A15 |
| AACCAATGGCCTCTTGAACGTGCATTGCGCTCTTGGGATATGCTTGAGAGCATGTCTGCT |
| TCAGTGCTTCTACTTTCTTCTCTGGTGCTCTTGTTATCATGAGCAGTGCTGCTGCATGCT |
| TCTGCAATTGGCACTGGCATGCTAAGTACCAAGTTTTGCTTGCTGTTGTGACTGATCAAC |
| ATCTCATGTCGTTTCAGTTAGCGAAACAAAGGCTTGTGTGTTCCAACACTTCCTAGCATG |
| AAGTCAGATAA |
| >GS_A15a |
| AACCAATGGCCTCTTGAACGTGCATTGCGCTCTTGGGATATGCTTGAGAGCATGTCTGCT |
| TCAGTGCTTCTACTTTCTTCTCTGGTGCTCTTGTTATCATGAGCAGTGCTGCTGCATGCT |
| TCTGCAATTGGCACTGGCATGCTAAGTACCAAGTTTTGCTTGCTGTTGTGACTGATCAAC |
| ATCTCATGTCGTTTCAGTTGGCGAAACAAAGGCTTGTGTGTTCCAACACTTCCTAGCATG |
| AAGTCAGATAA |
| >GS_A15b |
| AATCAATGGCCTCTTGAACGTGCATTGCGCTCTTGGGATATGCTTGAGAGCATGTCTGCT |
| TCAGTGCTTCTACTTTCTTCTCTGGTGCTCTTGTTATCATGAGCAGTGCTGCTGCATGCT |
| TCTGCAATTGGCACTGGCATGCTAAGTACCAAGTTTTGCTTGCTGTTGTGACTGATCAAC |
| ATCTCATGTCGTTTCAGTTAGCGAAACAAAGGCTTGTGTGTTCCAACACTTCCTAGCATG |
| AAGTCAGATAA |
| >GS_A15c |
| AACCAATGGCCTCTTGAACGTGCATTGCGCTCTTGGGATATGCTTGAGAGCATGTCTGCT |
| TCAGTGCTTCTACTTTCTTCTCTGGTGCTCTTGTTATCATGAGCAGTGCTGCTGCATGCT |
| TCTGCAATTGGCACTGGCATGCTAAGTACCAAGTTTTGCTTGCTGTTGTGACTGATCAAC |
| ATCTCATGTCGTTTCAGTTAGCGAAACAAAGGCTTGTGTGCTCCAACACTTCCTAGCATG |
| AAGTCAGATAA |
| >GS_A16 |
| AACCAATGGCCTCTTGAACGTGCATTGCGCTCTTGGGATATGCCTGAGAGCATGTCTGCT |
| TCAGTGCTTCTACTTTCTTTTCTGCTGCTCTTGTTATCATGAGCAGTGCTGCTGCATGCT |
| TCTGCAATTGGCACTGGCATGCTAGGTACCAAGTTTTGCTTGCTGTTGTGACTGATCAAC |
| ATCTCATGTCGTTTCAGTTGGCGAAACAAAGGCTTGTGTGTTCCAACACTTCCTAGCATG |
| AAGTCAGATAA |
| >ST_FREE(A) |
| AACCAATGGCCTCTTGAACGTGCATTGCGCTCTTGGGATATGCCTGAGAGCATGTCTGCTTCAGTGCTTCTACTTTTCCTATTCCTGCTGCTCCTTTCAAGGGGTGGTGCTGTTGTGTGCTACTGCATACTTTGCATTGGCATGCTCAGTATTAAGCATTGCCCACTGGGTTGACTGATCAACGTTTCATGTCTTTTTCAGTCGGGCAACTCAACACCTGGTGTCTTGAACACTTCCTAGCATGAAGTCAGACAAGTGA |
| >GS_B1 |
| AACCGATGGCCTCCTGAACGCGCATTGCGCTCTCGGGATTTCCTGAGAGCAGGTCTGCTT |
| CAGTGCTTAGCATTATCTACCTGTGCTTGCAAGCAGCATGTATGTCTGCATTGCTGCTTC |
| GCTTTCCAACAAGTCATCGATCGCTTTTGTGTTCGTAAATGGCTTGTTTGCTGCCTGGCC |
| CATGCGCCAAGCTTGAGCGTACTGTTGTTCCAAGCTTTGCTTGCATCGTGCAGCTCAAGC |
| GCGCAGCTGTCGGGATGCTGATGCATGCCCTTAGCATGA |
| >GS_B1a |
| AACCGATGGCCTCCTGAACGCGCATTGCGCTCTCGGGATTTCCTGAGAGCAGGTCTGCTT |
| CAGTGCTTAGCATTATCTACCTGTGCTTGCAAGCAGCATGTATGTCTGCATTGCTGCTTC |
| GCTTTCCAACAAGCCATCGATCGCTTTTGTGTTCGTAAATGGCTTGTTTGCTGCCTGGCC |
| CATGCGCCAAGCTTGAGCGTACTGTTGTTCCAAGCTTTGCTTGCATCGTGCAGCTCAAGC |
| GCGCAGCTGTCGGGATGCTGATGCATGCCCTTAGCATGA |
| >GS_B1b |
| AACCGATGGCCTCCTGAACGCGCATTGCGCTCTCGGGATTTCCTGAGAGCAGGTCTGCTT |
| CAGTGCTTAGCATTATCTACCTGTGCTTGCGAGCAGCGTATGTCTGCATTGCTGCTTCGC |
| TTTCCAACAAGTCATCGATCGCTTCGTAAATGGCTTGTTTGCTGCCTGGCCCACGTGCCA |
| AGCTTGAGCGTACTGTTGTTCCAAGCTTTGCTTGCATCGTGCAGCTCAAGCGCGCAGCTG |
| TCGGGATGCTGATGCATGCCCTTAGCATGA |
| >GS_B1d |
| AACCGATGGCCTCCTGAACGCGCATTGCGCTCTCGGGATTTCCTGAGAGCAGGTCTGCTT |
| CAGTGCTTAGCATTATCTACCTGTGCTTGCAAGCAGCATGTATGTCTGCATTGCTGCTTC |
| GCTTTCCAACAAGTCATTGATCGCTTTTGTGTTCGTAAATGGCTTGTTTGCTGCCTGGCC |
| CATGCGCCAAGCTTGAGCGTACTGTTGTTCCAAGCTTTGCTTGCATCGTGCAGCTCAAGC |
| GCGCAGCTGTCGGGATGCTGATGCATGCCCTTAGCATGA |
| >GS_B1e |
| AACCGATGGCCTCCTGAACGCGCATTGCGCTCTCGGGATTTCCTGAGAGCAGGTCTGCTT |
| CAGTGCTTAGCATTATCTACCTGTGCTTGCAAGCAGCATGTATGTCTGCATTGCTGCTTC |
| GCTTTCCAACAAGTCATCAATCGCTTTTGTGTTCGTAAATGGCTTGTTTGCTGCCTGGCC |
| CATGCGCCAAGCTTGAGCGTACTGTTGTTCCAAGCTTTGCTTGCATCGTGCAGCTCAAGC |
| GCGCAGCTGTCGGGATGCTGATGCATGCCCTTAGCATGA |
| >GS_B1g |
| AACCGATGGCCTCCTGAACGCGCATTGCGCTCTCGGGATTTCCTGAGAGCAGGTCTGCTT |
| CAGTGCTTAGCATTATCTACCTGTGCTTGCAAGCAGCATGTATGTCTGCATTGCTGCTTC |
| GCTTTCCAACAAGTCATCGACCGCTTTTGTGTTCGTAAATGGCTTGTTTGCTGCCTGGCC |
| CCACGCGCCAAGCTTGAGCGTACTGTTGTTCCAAGCTTTGCTTGCATCGTGCAGCTCAAG |
| CGCGCAGCTGTCGGGATGCTGATGCATGCCCTTAGCATGA |
| >GS_B1i |
| TTGTCTGACTTCATGCTAAGGGCATGCATCAGCATCCCGACAGCTGCGCGCTTGAGCTGCACGATGCAAGCAAAGCTTGGAACAACAGTACGCTCAAGCTTGGCGCATGGGCCAGGCAGCAAACAAGCCATTTACGAACACAAAAGCGATCGATGACGTGTTGGAAAGCGAAGCAGCAATGCAGACATACATGCTGCTTGCAAGCACAGGTAGATAATGCTAAGCACTGAAGCAGACCTGCTCTCAGGAAATCCCGAGAGCGCAATGCGCGTTCAGGAGGCCATCGGTT |
| >GS_B1j |
| AACCGATGGCCTCCTGAACGCGCATTGCGCTCTAGGGATTTCCTGAGAGCAGGTCTGCTT |
| CAGTGCTTAGCATTATCTACCTGTGCTTGCAAGCAGCATGTATGTCTGCATTGCTGCTTC |
| GCTTTCCAACAAGTCATCGATCGCTTTTGTGTTCGTAAATGGCTTGTTTGCTGCCTGGCC |
| CATGCGCCAAGCTTGAGCGTACTGTTGTTCCAAGCTTTGCTTGCATCGTGCAGCTCAAGC |
| GTGCAGCTGTCGGGATGCTGATGCATGCCCTTAGCATGA |
| >GS_B1k |
| AACCGATGGCCTCATCGCTTTGTGTTCGTAAATGGCTTGTTTGCTGCCTGGCCCATGCGC |
| CAAGCTTGAGCATACTGTTGTTCCAAGCTTTGCTTGCATCGTCCAGCTCAAGCGCGCAGC |
| TGTCGGGATGCTGATGCATGCCCTTAGCATGA |
| >GS_B1L |
| AACCGATGGCCTCCTGAACGCGCATTGCGCTCTCGGGATTTCCTGAGAGCAGGTCTGCTT |
| CAGTGCTTAGCATTATCTACCTGTGCTTGCAAGCAGCATGTATGTCTGCATTGCTGCTTC |
| GCTTTCCAACAAGTCATCGATCGCTTTTGTGTTCGTAAATGGCTTGTTTGCTGCCTGGCC |
| CACGCGCCAAGCTTGAGCGTACTGTTGTTCCAAGCTTTGCTTGCATCGTGCAGCTCAAGC |
| GCGCAGCTGTCGGGATGCTGATGCATGCCCTTAGCATGA |
| >GS_B1m |
| AACCGATGGCCTCCTGAACGCGCATTGCGCTCTCGGGATTTCCTGAGAGCAGGTCTGCTT |
| CAGTGCTTAGCATTATCTACCTGTGCTTGCAAGCAGCATGTATGTCTGCATTGCTGCTTC |
| GCTTTCCGACAAGTCATCGATCGCTTTTGTGTTCGTAAATGGCTTGTTTGCTGCCTGGCC |
| CATGCGCCAAGCTTGAGCGTACTGTTGTTCCAAGCTTTGCTTGCATCGTGCAGCTCAAGC |
| GCGCAGCTGTCGGGATGCTGATGCATGCCCTTAGCATGA |
| >GS_B2 |
| AACCGATGGCCTCCTGAACGCGCATTGCGCTCTCGGGATTTCCTGAGAGCAGGTCTGCTT |
| CAGTGCTTAGCATTATCTACCTGTGCTTGCAAGCAGCATGTCTACACTGCTGCTTTGCTT |
| TCCAACAAGTCATCGATCGCGTTTGTGTTCGTAAATGGCTTGTTTGCTGCCTGGCCCATG |
| CGCCAAGCTTGAGCGTACTGTTGTTCCAAGCTTAGCTTGCATCGTACAGCTCAAGCGCGC |
| AGCTGTTGGGATGCTGATGCATGCCCTTAGCATGA |
| >GS_B3 |
| AACCGATGGCCTCCTGAACGCGCATTGCGCTCTCGGGATTTCCTGAGAGCAGGTCTGCTT |
| CAGTGCTTAGCATTATCTACCTCTGCTTGCAAGCAGCATGTCTACACTGCTGCTTTGCTT |
| TCCAAGAAGTCATCGATCGCGTTTGTGTTCGTAAATGGCTTGTTTGCTGCCTGGCCCATG |
| CGCCAAGTTTGAGCGTACTGTTGTTCCAAGCTTAGCTTGCATCGTACAGCTCAAGCGCGC |
| AGCTGTTGGGATGCTGATGCATGCCCTTAGCATGA |
| >GS_B4 |
| AACCGATGGCCTCCTGAACGCGCATTGCGCTCTCGGGATTTCCTGAGAGCACGTCTGCTT |
| CAGTGCTTAGCCTTATTTACTTGTGCTTGCAAGCAGCATGTGTGCACTGCTGCTTTGCTT |
| TCCAACAAGTCACTGGCGTGTAGTGGCTTGTTTGCTGCCTGGCCCATGCGCCAAGCTTGA |
| GCGTACTGTTGTTCCAAGCTTATGCCTGCCTGGTGTAGCTTGCATCATGCTGCTCAAGCG |
| CGCAGCTGTCGGGATGCTGGTGCATGCCCTTAGCATGA |
| >GS_B5 |
| AACCGATGGCCTCCTGAACGCGCATTGCGCTCTCGGGATTTCCTGAGAGCAGGTCTGCTT |
| CAGTGCTTAGCATTATCTACCTGTGCTTGCAAGCAGCATGTATGTCTGCACTGCTGCTTC |
| GCTTTCCAACAAGTCATCGATCGCTTTTGTGTTCGTAAATGGCTTGTTTGCTGCCTGGCC |
| CATGCGCCAAGCTTGAGCGTACTGTTGTTCCAAGCTTTGCTTGCATCGTGCAGCTCAAGC |
| GCGCAGCTGTCGGGATGCTGATGCATGCCCTTAGCATGA |
| >GS_B5a |
| AACCGATAGCCTCCTGAACGCGCATTGCGCTCTCGGGATTTCCTGAGAGCAGGTCTGCTT |
| CAGTGCTTAGCATTATCTACCTGTGCTTGCAAGCAGCATGTATGTCTGCACTGCTGCTTC |
| GCTTTCCAACAAGTCATCGATCGCTTTTGTGTTCGTAAATGGCTTGTTTGCTGCCTGGCC |
| CATGCGCCAAGCTTGAGCGTACTGTTGTTCCAAGCTTTGCTTGCATCGTGCAGCTCAAGC |
| GCGCAGCTGTCGGGATGCTGATGCATGCCCTTAGCATGA |
| >GS_B6 |
| AACCGATGGCCTCCTGAACGCGCATTGCGCTCTCGGGATTTCCTGAGAGCAGGTCTGCTT |
| CAGTGCTTAGCATTATCTACCTGTGCTTGCAAGCAGCATGTCTACACTGCTGCTTCGCTT |
| TCCAACAAGTCATCGATCGCGTTTGTGTTCGTAAATGGCTTGTTTGCTGCCTGGCCCATG |
| CGCCAAGCTTGAGCGTACTGTTGTTCCAAGCTTAGCTTGCTTGCATCGTACAGCTCAAGC |
| GCGCAGCTGTTGGGATGCTGATGCATGCCCTTTAGCATGA |
| >GS_B7 |
| AACCGATGGCCTCCTGAACGCGCATTGCGCTCTCGGGATTTCCTGAGAGCAGGTCTGCTT |
| CAGTGCTTAGCATTCTCTACCTGTGCTTGCAAGCAGCATGTATGTCTGCATTGCTGCTTC |
| GCTTTCCAACAAGTCATCGATCGCTTTTGTGTTCGTAAATGGCTTGTTTGCTGCCTGGCC |
| CATGCGCCAAGCTTGAGCGTACTGTTGTTCCAAGCTTTGCTTGCATCGTGCAGCTCAAGC |
| GCGCAGCTGTCGGGATGCTGATGCATGCCCTTAGCATGA |
| >GS_B8 |
| AACCGATGGCCTCCTGAACGCGCATTGCGCTCTCGGGATTTCCTGAGAGCAGGTCTGCTT |
| CAGTGCTTAGCATTATCTACCTGTGCTTGCAAGCAGCATGTATGTCTGCATTGCTGCTTC |
| GCTTTCCCAACAAGTCATCGATCGCTTTTGTGTTCGTAAATGGCTTGTTTGCTGCCTGGC |
| CCACGCGCCAAGCTTGAGCGTACTGTTGTTCCAAGCTTTGCTTGCATCGTGCAGCTCAAG |
| CGCGCAGCTGTCGGGATGCTGATGCATGCCCTTAGCATGA |
| >GS_B9 |
| AACCGATGGCCTCCTGAACGTGCATTGCGCTCTCGGGGTTTCCTGAGAGCAGGTCTGCTT |
| CAGTGCTTAGCATTATCTACCTGTGCTTGCAAGCAGCATGTCTACACTGCTGCTTCGCTT |
| TCCAACAAGTCATCGATCGCGTTTGTGTTCGTAAATGGCTTGTTTGCTGCCTGGCCCATG |
| CGCCAAGCTTGAGCGTACTGTTGTTCCAAGCTTAGCTTGCATCGTATAGCTCAAGCGCGC |
| AGCTGTTGGGATGCTGATGCATGCCCTTAGCATGA |
| >GS_B10 |
| AACCGATGGCCTCCTGAACGCGCATTGCGCTCTCGGGATTTCCTGAGAGCAGGTCTGCTT |
| TAGTGCTTAGCATTATCTACCTGTGCTTGCAAGCAGCATGTATGTCTGCATTGCTGCTTC |
| GCTTTCCAACAAGTCATCGATCGCTTTTGTGTTCGTAAATGGCTTGTTTGCTGCCTGGCC |
| CATGCGCCAAGCTTGAGCGTGCTGTTGTTCCAAGCTTTGCTTGCATCGTGCAGCTCAAGC |
| GCGCAGCTGTCGGGATGCTGATGCATGCCCTTAGCATGA |
| >GS_B11 |
| AACCGATGGCCTCCTGAACGCGCATTGCGCTCTCGGGATTTCCTGAGAGCAGGTCTGCTT |
| CAGTGCTTAGCATTATCTACCTGTGCTTGCAAGCAGCATGTATGTCTGCACACTGCTGTT |
| TCGCTTTCCAACAAGTCATCGATCGCTTTTGTGTTCGTAAATGGCTTGTTCGCTGCCTGG |
| CCCATGCGCCAAGCTTGAGCGTACTGTTGTTCCAAGCTTTGCTTGCATCGTGCTGCTCAA |
| GCGCGCAGCTGTCGGGATGCTGATGCATGCCCTTAGCATGA |
| >GS_B13 |
| TGAACGCGCATTGCGCTCTCGGGATTTCCTGAGAGCAGGTCTGCTTCAGTGCTTAGCATT |
| CTCTACCTGTGCGTGCAAGCAGCATGTATGTCTGCATTGCTGCTTCGCTTTCCAACAAGT |
| CATCGATCGCTTTTGTGTTCGTAAATGGCTTGTTTGCTGCCTGGCCATGCGCCAAGCTTG |
| AGCGTACTGTTGTTCCAAGCTTTGCTTGCATCGTGCAGCTCAAGCGCGCAGCTGTCGGGA |
| TGCTGATGCATGCCCTTAGCATGA |
| >GS_B14 |
| AACCGATGGACCTCCTGAACGCGCACTGCGCTCTCGGGATTTCCTGAGAGCAGGTCTGCT |
| TCAGTGCTTAGCATTATCTACCTGTGCTTGCAAGCAGCATGTATGTCTGCATTGCTGCTT |
| CGCTTTCCAACAAGTCATCGATCGCTTTTGTGTTCGTAAATGGCTTGTTTGCTGCCTGGC |
| CCATGCGCCAAGCTTGAGCGTACTGTTGTTCCAAGCTTTGCTTGCATCGTGCAGCTCAAG |
| CGCGCAGCTGTCGGGATGCTGATGCATGCCCTTAGCATGA |
| >GS_B15 |
| AACCGATGGCCTCCTGAACGCGCAKTGCGCTCTCGGGATTTCCTGAGAGCAGGTCTGCTT |
| CAGTGCTTAGCATTATCTACCTGTGCTTGCAAGCAGCGTGTCTACACTGCTGCTTCGCTT |
| TCCAACAAGTCATCGATCGCGTTTGTGTTCGTAAGTGGCTTGTTTGCTGCCTGGCCCATG |
| CGCCAAGCTTGAGCGTACTGTTGTTCCAAGCTTTGCTTGCATCGTACAGCTCAAGCGCGC |
| AGCTGTTGGGATGCTGATGCATGCCCT |
| >GS_B17 |
| AACCGATGGCCTCCTGAACGCGCATTGCGCTCTCGGGATTTCCTGAGAGCAGGTCTGCTT |
| CAGTGCCTAGCATTATCTACCTGTGCTTGCAAGCAGCATGTATGTCTGCATTGCTGCTTC |
| GCTTTCCAACAAGTCATCGATCGCTTTTGTGTTCGTAAATGGCTTGTTTGCTGCCTGGCC |
| CATGCGCCAAGCTTGAGCGTACTGTTGTTCCAAGCCTTGCTTGGATCGTGCAGCTCAAGC |
| GCGCAGCTGTCGGGATGCTGATGCATGCCCTTAGCATGA |
| >GS_B18 |
| AACCGATGGCCTCCTGAACGCGCATTGCGCTCTCGGGATTTCCTGAGAGCAGGTCTGCTT |
| CAGTGCTTAGCATTATCTACCTGTGCTTGCAAGCAGCATGTCTACACTGCTGCTTCGCTT |
| TCCAACAAGTCATCGATCGCATTTGTGTTCGTAAATGGCTTGTTTGCTGCCTGGCCCATG |
| CGCCAAGTTTGAGCGTACTGTTGTTCCAAGCTTAGCTTGCATCGTACAGCTCAAGCGCGC |
| AGCTGTTGGGATGCTGATGCATGCCCTTAGCATGA |
| >ST_B18a_FB4A |
| AACCGATGGCCTCCTGAACGCGCATTGCGCTCTCGGGATTTCCTGAGAGCAGGTCTGCTTCAGTGCTTAGCATTATCTACCTGTGCTTGCAAGCAGCATATCTACACTGCTGCTTCGCTTTCCAACAAGTCATCGATCGCATTTGTGTTCGTAAATGGCTTGTTTGCTGCCTGGCCCATGCGCCAAGTTTGAGCGTACTGTTGTTCCAAGCTTAGCTTGCATCGTACAGCTCAAGCGCGCAGCTGTTGGGATGCTGATGCATGCCCTTAGCATGAAGTCAGACAAGAGA |
| >GS_B19 |
| AACCGATGGCCTCCTGAACGCGCATTGCGCTCTCGGGATTTCCTGAGAGCAGGTCTGCTT |
| CAGTGCTTAGCATTATCTACCTGTGCTTGCAAGCAGCATGTCTACACTGCTGCTTCGCTT |
| TCCAACAAGTCATCGATCGCGTTTGTGTTCGTAAATGGCTTGTTTGCTGCCTGGCCCATG |
| CGCCAAGCTTGAGCGTACTGTTGTTCCAAGCTTAGCTTGCATCGTACAGCTCAAGCGCGC |
| AGCTGTTGGGATGCTGATGCATGCCCTTAGCATGA |
| >GS_B20 |
| AACCGATGGCCTCCTGAACGCGCATTGCGCTCTCGGGATTTCCTGAGAGCAGGTCTGCTT |
| CAGTGCTTAGCATTATCTACCTGTGCTTGCAAGCAGCATGTATGTCTGCATTGCTGCTTC |
| GCTTTCCAACAAGTCATCGATCGCTTTTGTGCGATCGTAAATGGCTTGTTTGCTGCCTGG |
| CCCATGCGCCAAGCTTGAGCGTACTGTTGTTCCAAGCTTTGCTTGCATCGTGCAGCTCAA |
| GCGCGCAGCGTCGGGATGCTGATGCATGCCCTTAGCATGA |
| >GS_B21=B38 |
| AACCGATGGCCTCCTGAACGCGCATTGCGCTCTCGGGATTTCCTGAGAGCAGGTCTGCTT |
| CAGTGCTTAGCATTATCTACCTGTGCTTGCAAGCAGCATGTCTACACTGCTGCTTCGCTT |
| TCCAACAAATCATCGCGTTTGTGTTCGTAAATGGCTTGTTTGCTGCCTGGCCCATGCGCC |
| AAGCTTGAGCGTAGTGTTGTTCCAAGCTTAGCTTGCATCGTACAGCTCAAGCGCGCAGCT |
| GTTGGGATGCTGATGCATGCCCTTAGCATGA |
| >LJ_B23 |
| TTGTCTGACTTCATGCTAAGGGCATGCATCAGCATCCCAACAGCTGCGCGCTTGAGCTGTACGATGCAAGCTAAGCTTGGAACAACAGTACGCTCAAACTTGGCGCATGGGCCAGGCAGCAAACAAGCCATTTACGAACACACACACGATCGATGACTTGTTGGAAAGCGAAGCAGCAGTGTAGACATGCTGCTTGCAAGCAGAGGTAGATAAATGCTAAGCACTGAAGCAGACCTGCTCTCAGGGAATCCCGAGAGCGCAATGCGCGTTCAGGAGCCCATCGATT |
| >GS_B30 |
| AATCGATGGGCTCCTGAACGCGCATTGCGCTCTCGGGATTCCCTGAGAGCAGGTCTGCTT |
| CAGTGCTTAGCATTTATCTACCTCTGCTTGCAAGCAGCATGTCTACACTGCTGCTTCGGT |
| TTCCAACAAGTCATCGATCGTGTGTGTATTCGTAAATGGCTTGTTTGCTGCCTGGCCCAT |
| GCGCCAAGTTTGAGCGTACTGTTGTTCCAAGCTTAGCTTGCATCGTACAGCTCAAGCGCG |
| CAGCTGTTGGGATGCTGATGCATGCCCTTAGCATGA |
| >GS_B31 |
| AACCGATGGCCTCCTGAACGCGCATTGCGCTCTCGGGATTTCCTGAGAGCAGGTCTGCTT |
| CAGTGCTTAGCATTATCTACCTGTGCTTGCAAGCAGCATGCATGTCTGCATTGCTGCTTC |
| GCTTTCCAACAAGTCATCGATCGCTTTTGTGTTCGTAAATGGCTTGTTTGCTGCCTGGCC |
| CATGCGCCAAGCTTGAGCGTACTGTTGTTCCAAGCTTTGCTTGCATCGTGCAGCTCAAGC |
| GCGCAGCTGTCGGGATGCTGATGCATGCCCTTAGCATGA |
| >GS_B31a |
| AACCGATGGCCTCCTGAACGCGCATTGCGCTCTCGGGATTTCCTGAGAGCAGGTCTGCTT |
| CAGTGCTTAGCATTATCTACCTGTGCTTGCAAGCAGCATGCATGTCTGCATTGCTGCTTC |
| GCTTTCCAACAAGTCATCGATCGCTTTTGTGTTCGTAAATGGCTTGTTTGCTGCCTGGCC |
| CATGCGCCAAGCTTGAGCGTACTGTTGTTCCAAGCTTTGCTTGCATCGTGCACCTCAAGC |
| GCGCAGCTGTCGGGATGCTGATGCATGCCCTTAGCATGA |
| >GS_B32 |
| AACCGATGGCCTCCTGAACGCGCATTGCGCTCTCGGGATTTCCTGAGAGCAGGTCTGCTT |
| CAGTGCTTAGCATTATCTACCTGTGCTTGCAAGCAGCATGCATGTCTGCATTGCTGCTTC |
| GCTTTCCAACAAGTCATCGATCGCTTTTGTGTTCGTAAATGGCTTGTTTGCTGCCTGGCC |
| CATGCGCCAAGCTTGAGCGTACTATTGTTCCAAGCTTTGCTTGCATCGTGCAGCTCAAGC |
| GCGCAGCTGTCGGGATGCTGATGCATGCCCTTAGCATGA |
| >LJ_B33 |
| TTGTCTGACTTCATGCTAAGGGCATGCATCAGCATCCCAACAGCTGCGCGCTTGAGCTGTACAATGCAAGCTAAGCTTGGAACAACAGTACGCTCAAGCTTGGCGCATGGGCCAGGCAGCAAACAAGCCATTTACGAACACAAACGCGATCGATGACTTGTTGGAAAGCGAAGCAGCAGTGTGGACATGCTGCTTGCAAGCACAGGTAGATAATGCTAAGCACTGAAGCAGACCTGCTCTCAGGAAATCCCGAGAGCGCAATGCGCGTTCAGGAGGCCATCGGTT |
| >LJ_B36 |
| TTGTCTGACTTCATGCTAAGGGCATGCATCAGCATCCCGACAGCTGCGCGCTTGAGCTGCACGATGCAAGCAAAGCTTGGAACAACAGTACGCTCAAGCTTGGCGCATGGGCCAGGCAGCAAACAAGCCATTTACGAACACAAAAGCGATCGATGACTTGTTGGAAAGTGAAGCAGCAATGCAGACATACATGCTGCTTGCAAGCACAGGTAGATAATGCTAAGCACTGAAGCAGACCTGCTCTCAGGAAATCCCGAGAGCGCAATGCGCGTTCAGGAGGCCATCGGTT |
| >GS_B37 |
| AACCGATGGCCTCCTGAACGCGCATTGCGCTCTCGGGATTTCCTGAGAGCAGGTCTGCTT |
| CAGTGCTTTTAGCATTATCTACCTGTGCTTGCAAGCAGCATGTATGTCTGCATTGCTGCT |
| TCGCTTTCCAACAAGTCATCGATCGCTTTTGTGTTCGTAAATGGCTTGTTTGCTGCCTGG |
| CCCATGCGCCAAGCTTGAGCGTACTGTTGTTCCAAGCTTTGCTTGCATCGTGCAGCTCAA |
| GCGTGCAGCTGTCGGGATGCTGATGCATGCCCTTAGCATGA |
| >GS_B40 |
| AACCGATGGCCTCCTGAACGCGCATTGCGCTCTCGGGATTTCCTGAGAGCAGGTCTGCTT |
| CAGTGCTTTTAGCATTTATCTACCTGTGCTTGCAAGCAGCGCTTCGCTTTCCAACAAGTC |
| ATCGATCGCGTTTGTGTTCGTAAGTGGCTTGTTTGCTGCCTGGCCCATGCGCCAAGCTTG |
| AGCGTACCGTACTGTTGTTCCAAGCTTTGCTTGCATCGTACAGCTCAAGCGCGCAGCTGT |
| TGGGATGCTGATGCATGCCCCTTAGCATGA |
| >ST_B02_71 |
| GCATTGCGCTCTCGGGATTTCCTGAGAGCAGGGCTGATTCAGTGCTTAGCATTATCTACCTGTGCTTGCAAGCAGCATGTCTACACATGTCTACACTGCTGCTTCGCTTTCCAACAAGTCATCGATCGCGTTTGTGTTCGTAAATGGCTTGTTTGCTGTGCCTGGCCTTAGCTTGCATAGAAACCCCTGCAACTAACGTAGGGATCCCAATGCATGCCCTTAGCATGAAGTCAGACAAG |
| >GS_Cspa |
| AAGCAATGGCCTCCTGAACGTGCGTTGCACTCTTGGGATTTCCTGAGAGTATGTCTGCTT |
| CAGTGCTTAACTTGCCCCAACTTTGCAAGCAGGATGTGTTTCTGCCTTGCGTTCTTATGA |
| GCTATTGCCCTCTGAGCCAATGGCTTGTTAATTGCTTGGTTCTTGCAAAATGCTTTGCGC |
| GCTGTTATTCAAGTTTCTACCTTCGTGGTTTTACTTGAGTGACGCTGCTCATGCTTGCAA |
| CTGCTGGGATGCAGGTGCATGCCTCTAGCATGAAGTCAGACAA |
| >GS_Cspb |
| AACCAATGGCCTCCTGAACGTGCGTTGCACTCTTGGGATTTCCTGAGAGTATGTCTGCTT |
| CAGTGCTTAACTTGCCCCAACTTTGCAAGCAGGATGTGTTTCTGCCTTGCGTTCTTATGA |
| GCTATTGCCCTCTGAGCCAATGGCTTGTTAATTGCTTGGTTCTTGCAAAATGCTTTGCGC |
| GCTGTTATTCAATTTTCTACCTTCGTGGTTTTACTTGAGTGACGCTGCTCATGCTTGCAA |
| CCGCTGGGATGCAGGTGCATGCCTCTAGCATGAAGTCAGACAA |
| >GS_Cspd |
| AACCAATGGCCTCCTGAACGTGCGTTGCACTCTTGGGATTTCCTGAGAGTATGTCTGCTT |
| CAGCGCTTAACTTGCCCCAACTTTGCAAGCAGGATGTGTTTCTGCCTTGCGTTCTTATGA |
| GCTATTGCCCTCTGAGCCAATGGCTTGTTAATTGCTTGGTTCTTGCAAAATGCTTTGCGC |
| GCTGCTATTCAGGTTTCTACCTTCGTGGTTTTACTTGAGTGACGCTGCTCATGCTTGCAA |
| CCGCTGGGATGCAGGTGCATGCCTCTAGCATGAAGTCAGACAA |
| >GS_Cspe |
| AACCAATGGCCTCCTGAACGTGCGTTGCACTCTTGGGATTTCCTGAGAGTATGTCTGCTT |
| CAGTGCTTAACTTGCCCCAACTTTGCAAGCAGGATGTGTTTCTGCCTTGCGTTCTTATGA |
| GCTATTGCCCTCTGAGCCAATGGCTTGTTAATTGCTTGGTTCTTGCAAGATGCTTTGCGC |
| GCTGTTATTCAAGGTTCTACCTTCGTGGTTTTACTTGAGTGACGCTGCTCATGCTTGCAA |
| CCGCTGGGATGCAGGTGTGCATGCCTCTAGCATGAAGTCAGACAA |
| >GS_Cspf |
| AAGCAATGGCCTCCTGAACGTGTGTTGCACTCTTGGGATTTCCTGAGAGTATGTCTGCTT |
| CAGTGCTTAACTTGCCCCAACTTTGCAAGCAGGATGTGTTTCTGCCTTGCGTTCTTATGA |
| GCTATTGCCCTCTGAGCCAAGGGCTTGTTAATTGCTTGGTTCTTGCAAAATGCTTTGCGC |
| GCTGTTATTCAAGTTTCTACCTTTGTGGTTTTACTTGAGTGACGCTGCGCATGGTTGCAA |
| CCGCTGGGATGCAGGTGCATGCCTCTAGCATGAAGTCAGACAA |
| >GS_C1 |
| AACCAATGGCCTCCTGAACGTGCGTTGCACTCTTGGGATTTCCTGAGAGTATGTCTGCTT |
| CAGTGCTTAACTTGCCCCAACTTTGCAAGCAGGATGTGTTTCTGCCTTGCGTTCTTATGA |
| GCTATTGCCCTCTGAGCCAATGGCTTGTTAATTGCTTGGTTCTTGCAAAATGCTTTGCGC |
| GCTGTTATTCAGGTTTCTACCTTCGTGGTTTTACTTGAGTGACGCTGCTCATGCTTGCAA |
| CCGCTGGGATGCAGGTGCATGCCTCTAGCATGAAGTCAGACAA |
| >GS_C1ca=C1b=C1e |
| AACCAATGGCCTCCTGAACGTGCGTTGCACTCTTGGGATTTCCTGAGAGTATGTCTGCTT |
| CAGTGCTTAACTTGCCCCAACTTTGCAAGCAGATGTGTTTCTGCCTTGCGTTCTTATGAG |
| CTATTGCCCTCTGAGCCAATGGCTTGTTAATTGCTTGGTTCTTGCAAAATGCTTTGCGCG |
| CTGTTATTCAGGTTTCTACCTTCGTGGTTTTACTTGAGTGACGCTGCTCATGCTTGCAAC |
| CGCTGGGATGCAGGTGCATGCCTCTAGCATGAAGTCAGACAA |
| >GS_C1c.C45 |
| AACCAATGGCCTCCTGAACGTGCGTTGCACTCTTGGGATTTCCTGAGAGTATGTCTGCTT |
| CAGTGCTTAACTTGCCCCAACTTTGCAAGCAGGATGTGTTTCTGCCTTGCGTTCTTATGA |
| GCTATTGCCCTCTGAGCCAATGGCTTGTGAATTGCTTGGTTCTTGCAAAATGCTTTGCGC |
| GCTGTTATTCAGGTTTCTACCTTCGTGGTTTTACTTGAGTGACGCTGCTCATGCTTGCAA |
| CCGCTGGGATGCAGGTGCATGCCTCTAGCATGAAGTCAGACAA |
| >GS_C1d |
| AATCAATGGCCTCCTGAACGTGCGTTGCACTCTTGGGATTTCCTGAGAGTATGTCTGCTT |
| CAGTGCTTAACTTGCCCCAACTTTGCAAGCAGGATGTGTTTCTGCCTTGCGTTCTTATGA |
| GCTATTGCCCTCTGAGCCAATGGCTTGTGAATTGCTTGGTTCTTGCAAAATGCTTTGCGC |
| GCGGTTATTCAGGTTTCTACCTTCGTGGTTTTACTTGAGTGACGCTGCTCATGCTTGCAA |
| CCGCTGGGATGCAGGTGCATGCCTCTAGCATGAAGTCAGACAA |
| >GS_C1f |
| AACCAATGGCCTCCTGAACGTGCGTTGCACTCTTGGGATTTCCTGAGAGTATGTCTGCTT |
| CAGTGCTTAACTTGCCCCAACTTTGCAAGCAGGATGTGTTTCTGCCTTGCATTCTTATGA |
| GCTATTGCCCTCTGAGCCAATGGCTTGTTAATTGCTTGGTTCTTGCAAAATGCTTTGCGC |
| GCTGTTATTCAGGTTTCTACCTTCGTGGTTTTACTTGAGTGACGCTGCTCATGCTTGCAA |
| CCGCTGGGATGCAGGTGCATGCCTCTAGCATGAAGTCAGACAA |
| >GS_C1g |
| AACCAATGGCCTCCTGAACGTGCGTTGCACTCTTGGGATTTCCTGAGAGCATGTCTGCTT |
| CAGTGCTTAACTTGCCCCAACTTTGCAAGCAGGATGTGTTTCTGCCTTGCGTTCTTATGA |
| GCTATTGCCCTCTGAGCCAATGGCTTGTTAATTGCTTGGTTCTTGCAAAATGCTTTGCGC |
| GCTGTTATTCAGGTTTCTACCTTCGTGGTTTTACTTGAGTGACGCTGCTCATGCTTGCAA |
| CCGCTGGGATGCAGGTGCATGCCTCTAGCATGAAGTCAGACAA |
| >GS_C1h |
| AACCAATGGCCTCCTGAACGTGCGTTGCACTCTTGGGATTTCCTGAGAGTATGTCTGCTT |
| CAGTGCTTAACTTGCCCCAACTTTGCAAGCAGGATGTGTTTCTGCCTTGTGTTCTTATGA |
| GCTATTGCCCTCTGAGCCAATGGCTTGTTAATTGCTTGGTTCTTGCAAAATGCTTTGCGC |
| GCTGTTATTCAGGTTTCTACCTTCGTGGTTTTACTTGAGTGACGCTGCTCATGCTTGCAA |
| CCGCTGGGATGCAGGTGCATGCCTCTAGCATGAAGTCAGACAA |
| >GS_C1i |
| AACCAATGGCCTCCTGAACGTGCGTTGCACTCTTGGGATTTCCTGAGAGTATGTCTGCTT |
| CAGTGCTTAACTTGCCCCAACTTTGCAAGCAGGATGTGTTTCTGCCTTGCGTTCTTATGA |
| GCCATTGCCCTCTGAGCCAATGGCTTGTTAATTGCTTGGTTCTTGCAAAATGCTTTGCGC |
| GCTGTTATTCAGGTTTCTACCTTCGCGGTTTTACTTGAGTGACGCTGCTCATGCTTGCAA |
| CCGCTGGGATGCAGGTGCATGCCTCTAGCATGAAGTCAGACAA |
| >GS_C1j |
| AACCAATGGCCTCCTGAACGTGCGTTGCACTCTTGGGATTTCCTGAGAGTATGTCTGCTT |
| CAGTGCTTACCTTGCCCCAACTTTGCAAGCAGGATGTGTTTCTGCCTTGCGTTCTTATGA |
| GCTATTGCCCTCTGAGCCAATGGCTTGTTAATTGCTTGGTTCTTGCAAAATGCTTTGCGC |
| GCTGTTATTCAGGTTTCTACCTTCGTGGTTTTACTTGAGTGACGCTGCTCATGCTTGCAA |
| CCGCTGGGATGCAGGTGCATGCCTCTAGCATGAAGTCAGACAA |
| >GS_C1k |
| AACCAATGGCCTCCTGAACGTGCGTTGCACTCTTGGGATTTCCTGAGAGTATGTCTGCTT |
| CAGTGCTTAACTTGCCCCAACTTTGCAAGCAGGATGTGTTTCTGCCTTGCGTTCTTATGA |
| GCTATTGCCCTCTGAGCCAATGGCTTGTGAATTGCTTGGTTCTTGCAAAATGCTTTGCGC |
| GCTGTTATTCAGGTTTCTACCTTCGTGGTTTTACTTGAGTGACGCTGCTCATGCTTGCAA |
| CCGCTGGGATGCACGTGCATGCCTCTAGCATGAAGTCAGACAA |
| >GS_C1m |
| AGCCAATGGCCTCCTGAACGTGCGTTGCACTCTTGGGATTTCCTGAGAGTATGTCTGCTT |
| CAGTGCTTAACTTGCCCCAACTTTGCAAGCAGGATGTGTTTCTGCCTTGCGTTCTTACGA |
| GCTATTGCCCTCTGAGCCAATGGCTTGTTAATTGCTTGGTTCTTGCAAAATGCTTTGCGC |
| GCTGTTATTCAGGTTTCTACCTTCGTGGTTTTACTTGAGTGACGCTGCTCATGCTTGCAA |
| CCGCTGGGATGCAGGTGCATGCCTCTAGCATGAAGTCAGACAA |
| >GS_C1r |
| AACCAATGGCCTCCTGAACGTGCGTTACACTCTTGGGATTTCCTGAGAGTATGTCTGCTT |
| CAGTGCTTAACTTGCCCCAACTTTGCAAGCAGGATGTGTTTCTGCCTTGCGTTCTTATGA |
| GCTATTGCCCTCTGAGCCAATGGCTTGTTAATTGCTTGGTTCTTGCAAAATGCTTTGCGC |
| GCTGTTATTCAGGTTTCTACCTTCGTGGTTTTACTTGAGTGACGCTGCTCATGCTTGCAA |
| CCGCTGGGATGCAGGTGCATGCCTCTAGCATGAAGTCAGACAA |
| >GS_C1s |
| AACCAATGGCCTCCTGAACGTGCGTTGCACTCTTGGGATTTCCTGAGAGTATGTCTGCTT |
| CAGTGCTTAACTTGCCCCAACTTTGCAAGCAGGATGTGTTTCTGCCTTGCGTTCTTATGA |
| GCTATTGCCCTCTGAGCCAATGGCTTGTTAATTGCTTGGTTCTTGCAAAATGCTTTGCGC |
| GCTGTTATTCAGGTGACGCTGCTCATGCTTGCAACCGCTGGGATGCAGGTGCATGCCTCT |
| AGCATGAAGTCAGACAA |
| >GS_C1t |
| AACCAATGGCCTCCTGAACGTGCGTTGCACTCTTGGGATTTCCTGAGAGTATGTCTGCTT |
| CAGTGCTTAACTTGCCCCAACTTTGCAAGCAGGATGTGTTTCTGCCTTGCGTTCTTATGA |
| GCTATTGCCCTCTGAGCCAATGGCTTGTGAATTGCTTGGTTCTTGCAAAATGCTTTGCGC |
| GCGGTTATTCAGGTTTCTACCTTCGTGGTTTTACTTGAGTGACGCTGCTCATGCTTGCAA |
| CCGCTGGGATGCAGGTGCATGCCTCTAGCATGAAGTCAGACAA |
| >GS_C1p=C1.8 |
| AATCAATGGCCTCCTGAACGTGCGTTGCACTCTTGGGATTTCCTGAGAGTATGTCTGCTT |
| CAGTGCTTAACTTGCCCCAACTTTGCAAGCAGGATGTGTTTCTGCCTTGCGTTCTTATGA |
| GCTATTGCCCTCTGAGCCAATGGCTTGTTAATTGCTTGGTTCTTGCAAAATGCTTTGCGC |
| GCTGTTATTCAGGTTTCTACCTTCGTGGTTTTACTTGAGTGACGCTGCTCATGCTTGCAA |
| CCGCTGGGATGCAGGTGCATGCCTCTAGCATGAAGTCAGACAA |
| >GS_C1aa |
| AACCAATGGCCTCCTGAACGTGCGTTGCACTCTTGGGATTTCCTGAGAGTATGTCTGCTT |
| CAGTGCTTAACTTCCAACTTTGCAAGCAGGATGTGTTTCTGCCTTGCGTGCTTATGAGCT |
| ATTGCCCTCTGAGCCAATGGTTTGTGAATCGCTTGGTTCTTGCAAAATGCTTTGCGCGCT |
| GTTATTCAGGTATCTACCTTCGTGGTTTTACTTGAGTGACGCTGCTCATGCTTGCAACCG |
| CTGGGATGCAGGTGCATGCCTCTAGCATGAAGTCAGACAA |
| >GS_C# |
| AACCAATGGCCTCCTGAACGTGCGTTGCACTCTTGGGATTTCCTGAGAGTATGTCTGCTT |
| CAGTGCTTAACTTGCCCCAACTTTGCAAGCAGGATGTGTTTCTGCCTTGCGTTCTTATGA |
| GCTATTGCCCTCTGAGCCAATGGCTTGTTAATTGCTTGGTTCTTGCAAACTGCTTTGCGC |
| GCTGTTATTCAGGTTTCTACCTTCGTGGTTTTACTTGAGTGACGCTGCTCATGCTTGCAA |
| CCGCTGGGATGCAGGTGCATGCCTCTAGCATGAAGTCAGACAA |
| >GS_C1# |
| AACCAATGGCCTCCTGAACGTGCGTTGCACCCTTGGGATTTCCTGAGAGTATGTCTGCTT |
| CAGTGCTTAACTTGCTCCAACTTTGCAAGCAGGATGTGTTTCTGCCTTGCGTTCTTATGA |
| GCTATTGCCTTCTGCGCCAATGGCTTGTTAATTGCTTGGTTCTTGCAAAATGCTTTGCGC |
| GCTGTTATTCAAGTTTCTACCTTCGCGGTTTTACTTGAGTGACGCTGCTCATGCTTGCAA |
| CCGCTGGGATGCAGGTGCATGCCTCTAGCATGAAGTCAGACAA |
| >GS_C1.5 |
| AACCAATGGCCTCCTGAACGTGCGTTGCACTCTTGGGATTTCCTGAGAGTATGTCTGCTT |
| CAGTGCTTAACTTGCCCCAACTTTGCAAGCAGGATGTGTTTCTGCCTTGCGTTCTTATGA |
| GCTATTGCCCTCTGAGCCAATGGCTTGTTAATTGCTTGGTTCTTGCAAAATGCTTTGCGC |
| GCTGTTATTCAGGTTTCTACCTTCGTGGTCTTACTTGAGTGACGCTGCTCATGCTTGCAA |
| CCGCTGGGATGCAGGTGCATGCCTCTAGCATGAAGTCAGACAA |
| >GS_C1.6 |
| AACCAATGGCCTCCTGAACGCGCGTTGCACTCTTGGGATTTCCTGAGAGTATGTCTGCTT |
| CAGTGCTTAACTTGCCCCAACTTTGCAAGCAGGATGTGTTTCTGCCTTGCGTTCTTATGA |
| GCTATTGCCCTCTGAGCCAATGGCTTGTTAATTGCTTGGTTCTTGCAAAATGCTTTGCGC |
| GCTGTTATTCAGGTTTCTACCTTCGTGGTTTTACTTGAGTGACGCTGCTCATGCTTGCAA |
| CCGCTGGGATGCAGGTGCATGCCTCTAGCATGAAGTCAGACAA |
| >GS_C1.7 |
| AACCAATGGCCTCCTGAACGTGCGTTGCACTCTTGGGATTTCCTGAGAGTATGTCTGCTT |
| CAGTGCTTAACTTGCCCCAACTTTGCAGGCAGGATGTGTTTCTGCCTTGCGTTCTTATGA |
| GCTATTGCCCTCTGAGCCAATGGCTTGTTAATTGCTTGGTTCTTGCAAAATGCTTTGCGC |
| GCTGTTATTCAGGTTTCTACCTTCGTGGTTTTACTTGAGTGACGCTGCTCATGCTTGCAA |
| CCGCTGGGATGCAGGTGCATGCCTCTAGCATGAAGTCAGACAA |
| >GS_C1.v1a |
| AACCAATGGCCTCCTGAACGTGCGTTGCACTCTTGGGATTTCCTGAGAGTATGTCTGCTT |
| CAGTGCTTAACTTGCCCCAACTTTGCAAGCAGGATGTGTTTCTGCCTTGCGTTCTTATGA |
| GCTATTGCCCTCTGAGCCAATGGCTTGTTAATTGCTTGGTTCTTGCAAAATGCTTTGCGC |
| GCTGTTATTCAGGTTTCTACCTTCGTGGTTTTACTTGAGTGACGCTGCTCATGCTTGCAA |
| CCGCTGGGATGCAGGTGCATGCCTCTAGCATGAAGTCAGATAA |
| >GS_C1.v1b |
| AACCAATGGCCTCCTGAACGTGCGTTGCACTCTTGGGATTTCCTGAGAGTATGTCTGCTT |
| CAGTGCTTAACTTGCCCCAACTTTGCAAGCAGGATGTGTTTCTGCCTTGCGTTCTTATGA |
| GCTATTGCCCTCTGAGCCAATGGCTTGTTAATTGCTTGGTTCTTGCAAAATGCTTTGCGC |
| GCTGTTATTCAGGTTTCTACCTTCGTGGTTTTACTTGAGTGACGTTGCTCATGCTTGCAA |
| CCGCTGGGATGCAGGTGCATGCCTCTAGCATGAAGTCAGATAA |
| >GS_C2 |
| AACCAATGGCCTCCTGAACGTGCGTTGCACTCTTGGGATTTCCTGAGAGTATGTCTGCTT |
| CAGTGCTTAACTTGCCCCAACTTTGCAAGCAGGATGTGTTTCTGCCTTGCGTTCTTATGA |
| GCTATTGCCTTCTGCGCCAATGGCTTGTTAATTGCTTGGTTCTTGCAAAATGCTTTGCGC |
| GCTGTTATTCAAGTTTCTACCTTCGCGGTTTTACTTGAGTGACGCTGCTCATGCTTGCAA |
| CCGCTTGGGATGCAGGTGCATGCCTCTAGCATGAAGTCAGACAA |
| >LJ_C2r |
| TTGTCTGACTTCATGCTAGAGGCATGCACCTGCATCCCAGCGGTTGCAAGCATGAGCAGCGTCACTCAAGTAAAACCACGAAGGTAGAAACTTGAATAACAGCGCGCAAAGCATTTTGCAAGAACCAAGCAATTAACAAGCCATTGGCTCAGAGGGCAATAGCTCATAAGAATGCAAGGCAGAAACACATCCTGCTTGCAAAGTTGGGGCAAGTTAAGCACTGAAGCAGACATACTCTCAGGAAATCCCAAGAGTGCAACGCACGTTCAGGAGGCCATTGGTTCACGGAGTT |
| >GS_C3_new |
| AACCAATGGCCTCCTGAACGTGCGTTGCACTCTTGGGATTTCCTGAGAGTATGTCTGCTT |
| CAGTGCTTAACTTGCCCCAACTTTGCAATCAGGATGTGTTTCTGCCTTGCGTTCTTATGA |
| ACTATTGCCCTCTGAGCCAATGGCTTGKTAATTGCTTGGTTCTTGCAAAATGCTTTGCGC |
| GCTGTTATTCAAGTTTCTACCTTCSTGGTTTTACTTGAGTGACGCTGCTCATGC |
| >GS_C3aa |
| AAGCAATGGCCTCCTGAACGTGCGTTGCACTCTTGGGATTTCCTGAGAGTATGTCTGCTT |
| CAGTGCTTAACTTGCCCCAACTTTGCAAGCAGGATGTGTTTCTGCCTTGCGTTCTTATGA |
| GCTATTGCCCTCTGAGCCAATGGCTTGTTAATTGCTTGGTTCCTGCAAAATGCTTTGCGC |
| GCTGTTATTCAAGTTTCTACCTTCGCGGTTTTACTTGAGTGACGCTGCTCATGCTTGCAA |
| CCGCTGGGATGCAGGTGCATGCCTCTAGCATGAAGTCAGACAA |
| >GS_C3ff |
| AATCAATGGCCTCCTGAACGTGCGTTGCACTCTTGGGATTTCCTGAGAGTATGTCTGCTT |
| CAGTGCTTAACTTGCCCCAACTTTGCAAGCAGGATGTGTTTCTGCCTTGCGTGCTTATGA |
| GCTATTGCCCTCTGAGCCAATGGCTTGTTACTTGCTTGGTTCTTGCAAAATGCTTTGCGC |
| GCTGTTATTCAAGTTTCTACCTTCGTGGTTTTACTTGAGTGACGCTGCTCATGCTTGCAA |
| CCGCTGGGATGCAGGTGCATGCCTCTAGCATGAAGTCAGACAA |
| >GS_C3a |
| AACCAATGGCCTCCTGAACGTGCGTTGCACTCTTGGGATTTCCTGAGAGTATGTCTGCTT |
| CAGTGCTTAACTTGCCCCAACTTTGCAAGCAGGATGTGTTTCTGCCTTGCGTTCTTATGA |
| GCTATTGCCCTCTGAGCCAATGGCTTGTTAATTGCTTGGTTCTTGCAAAATGCTTTGCGC |
| GCTGTTATTCAAGTTTCTACCTTCGTGGTTTTACTTGAGTGATGCTGCTCATGCTTGCAA |
| CCGCTGGGATGCAGGTGCATGCCTCTAGCATGAAGTCAGACAA |
| >GS_C3b |
| AACCAATGGCCTCCTGAACGTGCGTTGCACTCTTGGGATTTCCTGAGAGTATGTCTGCTT |
| CAGTGCTTAACTTGCCCAACTTTGCAAGCAGGATGTGTTTCTGCCTTGCGTTCTTATGAG |
| CTATTGCCCTCTGAGCCAATGGCTTGTTAATTGCTTGGTTCTTGCAAAATGCTTTGCGCG |
| CTGTTATTCAAGTTTCTACCTTCGTGGTTTTACTTGAGTGACGCTGCTCATGCTTGCAAC |
| CGCTGGGATGCAGGTGCATGCCTCTAGCATGAAGTCAGACAA |
| >GS_C3d=C21 |
| AACCAATGGCCTCCTGAACGTGCGTTGCACTCTTGGGATTTCCTGAGAGTATGTCTGCTT |
| CAGTGCTTAACTTGCCCCAACTTTGCAAGCATTTCTGCCTTGCGTTCTTATGAGCTATTG |
| CCCTCTGAGCCAATGGCTTGTTAATTGCTTGGTTCTTGCAAAATGCTTTGCGCGCTGTTA |
| TTCAAGTTTCTACCTTCGTGGTTTTACTTGAGTGACGCTGCTCATGCTTGCAACCGCTGG |
| GATGCAGGTGCATGCCTCTAGCATGAAGTCAGACAA |
| >GS_C3e |
| AACCAATGGCCCCCTGAACGTGCGTTGCACTCTTGGGATTTCCTGAGAGTATGTCTGCTT |
| CAGTGCTTAACTTGCCCCAACTTTGCAAGCAGGATGTGTTTCTGCCTTGCGTTCTTATGA |
| GCTATTGCCCTCTGAGCCAATGGCTTGTTAATTGCTTGGTTCTTGCAAAATGCTTTGCGC |
| GCTGTTATTCAAGTTTCTACCTTCGTGGTTTTACTTGAGTGACGCTGCTCATGCTTGCAA |
| CCGCTGGGATGCAGGTGCATGCCTCTAGCATGAAGTCAGACAA |
| >GS_C3f |
| AACCAATGGCCTCCTGAACGTGCGTTGCACTCTTGGGATTTCCTGAGAGTATGTCTGCTT |
| CAGTGCTTAACTTGCCCCAACTTTGCAAGCAGGATGTGTTTCTGCCTTGCGTTCTTATGA |
| GCTATTGCCCTCTGAGCCAATGGCTTGTTAATTGCTTGGTTCTTGCAAAATGCTTTGCGC |
| GCTGTTATTCAAGTTTCTACCTTCGTTGTTTTACTTGAGTGACGCTGCTCATGCTTGCAA |
| CCGCTGGGATGCAGGTGCATGCCTCTAGCATGAAGTCAGACAA |
| >GS_C3g |
| AACCAATGGCCTCCTGAACGTGTGTTGCACTCTTGGGATTTCCTGAGAGTATGTCTGCTT |
| CAGTGCTTAACTTGCCCCAACTTTGCAAGCAGGATGTGTTTCTGCCTTGCGTTCTTATGA |
| GCTATTGCCCTCTGAGCCAATGGCTTGTTAATTGCTTGGTTCTTGCAAAATGCTTTGCGC |
| GCTGTTATTCAAGTTTCTACCTTCGTGGTTTTACTTGAGTGACGCTGCTCATGCTTGCAA |
| CCGCTGGGATGCAGGTGTGCATGCCTCTAGCATGAAGTCAGACAA |
| >GS_C3h |
| AACCAATGGCCTCCTGAACGTGCGTTGCACTCTTGGGGTTTCCTGAGAGTATGTCTGCTT |
| CAGTGCTTAACTTGCCCCAACTTTGCAAGCATTTCTGCCTTGCGTTCTTATGAGCTATTG |
| CCCTCTGAGCCAATGGCTTGTTAATTGCTTGGTTCTTGCAAAATGCTTTGCGCGCTGTTA |
| TTCAAGTTTCTACCTTCGTGGTTTTACTTGAGTGACGCTGCTCATGCTTGCAACCGCTGG |
| GATGCAGGTGCATGCCTCTAGCATGAAGTCAGACAA |
| >GS_C3ha |
| AACCAATGGCCTCCTGAACGTGCGTTGCACTCTTGGGATTTCCTGAGAGTATGTCTGCTT |
| CAGTGCTTAACTTGCCCCAACTTTGCAAGCATTTCTGCCTTGCGTTCTTATGAGCTATTG |
| CCCTCTGAGCCAATGGTTTGTTAATTGCTTGGTTCTTGCAAAATGCTTTGCGCGCTGTTA |
| TTCAAGGTTCTACCTTCGTGGTTTTACTTGAGTGACGCTGCTCATGCTTGCAACCGCTGG |
| GATGCAGGTGCATGCCTCTAGCATGAAGTCAGACAA |
| >GS_C3j |
| AAGCAATGGCCTCCTGAACGTGCGTTGCACTCTTGGGATTTCCTGAGAGTATGTCTGCTT |
| CAGTGTTTAACTTGCCCCAACTTTGCAAGCAGGATGTGTTTCTGCCTTGCGTTCTTATGA |
| GCTATTGCCCTCTGAGCCAATGGCTTGTTAATTGCTTGGTTCTTGCAAAATGCTTTGCGC |
| GCTGTTATTCAAGTTTCTACCTTCGTGGTTTTACTTGAGTGACGCTGCTCATGCTTGCAA |
| CCGCTGGGATGCAGGTGCATGCCTCTAGCATGAAGTCAGACAA |
| >GS_C3k |
| AACCAATGGCCTCCTGAATGTGCGTTGCACTCTTGGGATTTCCTGAGAGTATGTCTGCTT |
| CAGTGCTTAACTTGCCCCAACTTTGCAAGCAGGATGTGTTTCTGCCTTGCGTTCGTATGA |
| GCTGTTGCCCTCTGAGCCAATGGCTTGTTAATTGCTTGGTTCTTGCAAAATGCTTTGCGC |
| GCTGTTATTCAAGTTTCTACCTTCGTGGTTTTACTTGAGTGATGCTGCTCATGCTTGCAA |
| CCGCTGGGATGCAGGTGCATGCCTCTAGCATGAAGTCAGACAA |
| >GS_C3L |
| AACCAATGGCCTCCTGAACGTGCGTTGCACTCTTGGGATTTCCTGAGAGTATGTCTGCTT |
| CAGTGCTTAACTTGCCCAACTTTGCAAGCAGGATGTGTTTCTGCCTGCGTTCTTATGAGC |
| TATTGCCCTCTGAGCCAATGGCTTGTTAATTGCTTGGTTCTTGCAAAATGCTTTGCGCGC |
| TGTTATTCAAGTTTCTACCTTCGTGGTTTTTTTACTTGAGTGACGCTGCTCATGCTTGCA |
| ACCGCTGGGATGCAGGTGCATGCCTCTAGCATGAAGTCAGACAA |
| >GS_C3n |
| AATCAATGGCCTCCTGAACGTGCGTTGCACTCTTGGGATTTCCTGAGAGTATGTCTGCTT |
| CAGTGCTTAACTTGCCCCAACTTTGCAAGCAGGATGTGTTTCTGCCTTGCGTTCTTATGA |
| GCTATTGCCCTCTGAGCCAATGGCTTGTTACTTGCTTGGTTCTTGCAAAATGCTTTGCGC |
| GCTGTTATTCAAGTTTCTACCTTCGTGGTTTTACTTGAGTGACGCTGCTCATGCTTGCAA |
| CCGCTGGGATGCAGGTGCATGCCTCTAGCATGAAGTCAGACAA |
| >GS_C3t |
| AACCAATGGCCTCCTGAACGTGCGTTGCACTCTTGGGATTTCCTGAGAGTATGTCTGCTT |
| CAGTGCTTAACTTGCCCCAACTTTGCAAGCAGGATGTGTTTCTGCCTTGCGTTCTTATGA |
| GCTATTGCCCTCTGAGCCAATGGCTTGTTACTTGCTTGGTTCTTGCAAAATGCTTTGCGC |
| GCTGTTATTCAAGTTTCTACCTTCGTGGTTTTACTTGAGTGACGCTGCTCATGCTTGCAA |
| ACGCTGGGATGCAGGTGCATGCCTCTAGCATGAAGTCAGACAA |
| >GS_C3p |
| AACCAATGGCCTCCTGAACGTGCGTTGCACTCTTGGGATTCCTGAGAGTATGTCTGCTTC |
| AGTGCTTGACTTGCCCCAACTTTGCAAGCAGGATGTGTTTCTGCCTTGCGTTCTTATGAG |
| CTATTGCCCTCTGAGCCAATGGCTTGTTAATTGCTTGGTTCTTGCAAAATGCTTTGCGCG |
| CTGTTATTCAAGTTTCTACCTTCGTTGTTTTACTTGAGTGACGCTGCTCATGCTTGCAAC |
| CGCTGGGATGCAGGTGCATGCCTCTAGCATGAAGTCAGACAA |
| >GS_C3q |
| AACCAATGGCCTCCTGAACGTGCGTTGCACTCTTGGGATTTCCTGAGAGTATGTCTGCTT |
| CAGTGCTTTAACTTGCCCCAACTTTGCAAGCATTTCTGCCTTGCGTTCATATGAGCTATT |
| GCCCTCTGAGCCAATGGCTTGTTAATTACTTGGTTCTTGCGAAATGGTTTGTGCGCTGTT |
| ATTCAAGTTTCTACCTTTGTGGTTTTACTTGAGTGACGCTGCTCATGCTTGCAACCGCTG |
| GGATGCAGGTGCATGCCTCTAGCATGAAGTCAGACAA |
| >LJ_C3s |
| TTGTCTGACTTCATGCTAGAGGCATGCACCTGCATCCCAGCGGTTGCAAGCATGAGCAGCGTCACTCAAGTAAAACCACGAAGGTAGAAACTTGAATAACAGCGCGCAAAGCATTTTGCAAGAACCAAGCAATTAACAAGCCATTGGCTCAGAGGGCAATAGCTCATAAGAACGCAAGGCAGAAACACATCCTGCTTGCAAAGTTGGGGCAAGTTAAGCACTGAAGCAGACATACTCTCAGGAAATCCCAAAAGTGCAACGCACGTTCAGGAGGCCATTGGTTCA |
| >GS_C3u |
| AAGCAATGGCCTCCTGAACGTGCGTTGCACTCTTGGGATTTCCTGAGAGTATGTCTGCTT |
| CAGTGCTTAACTTGCCCCAACTTTGCAAGCAGGATGTGTTTCTGCCTTGCGTTCTTATGA |
| GCTATTGCCCTCTGAGCCAATGGCTTGTTAATTGCTTGGTTCTTGCAAAATGCTTTGCGC |
| GCTGTTATTCAAGTTTCTACCTTCGCGGTTTTACTTGAGTGACGCTGCTCATGCTTGCAA |
| CCGCTGGGATGCAGGTGCATGCCTCTAGCATGAAGTCAGACAA |
| >GS_C3v |
| AACCAATGGCCTCCTGAACGTGCGTTGCACTCTTGGGATTTCCTGAGAGTATGTCTGCTT |
| CAGTGCTTAACTTGCCCCAACTTTGCAAGCAGGATGTGTTTCTGCCTTGCGTTCTTATGA |
| GCTATTGCCCTCTGAGCCAATGGCTTGTTCATTGCTTGGTTCTTGCAAAATGCTTTGCGC |
| GCTGTTATTCAGGTTTCTACCTTCGTGGTTTTACTTGAGTGATGCTGCTCATGCTTGCAA |
| CCGCTGGGATGCAGGGTGCATGCCTCTAGCATGAAGTCAGACAA |
| >GS_C3w |
| AATCAATGGCCTCCTGAACGTGCGTTGCACTCTTGGGATTTCCTGAGAGTATGTCTGCTT |
| CAGTGCTTAACTTGCCCCAACTTTGCAAGCAGGATGTGTTTCTGCCTTGCGTTCTTATGA |
| GCTATTGCCCTCTGAGCCAATGGCTTGTTAATTGCTTGGTTCTTGCAAAATGCTTTGCGC |
| GCTGTTATTCAAGTTTCTACCTTCGTGGTTTTACTTGAGTGACGCTGCTCATGCTTGCAA |
| CCGCTGGGATGCAGGTGCATGCCTCTAGCATGAAGTCAGACAA |
| >GS_C3y |
| AACCAATGGCCTCCTGAACGTGCGTTGCACTCTTGGGATTTCCTGAGAGTATGTCTGCTT |
| CAGTGCTTAACTTGCCCCAACTTTGCAAGCAGGATGTGTTTCTGCCTTGCGTTCTTATGA |
| GCTATTGCCCTCTGAGCCAGTGGCTTGTTAATTGCTTGGTTCTTGCAAAATGCTTTGCGC |
| GCTGTTATTCAAGTTTCTACCTTCGTGGTTTTACTTGAGTGACGCTGCTCATGCTTGCAA |
| CCGCTGGGATGCAGGTGCATGCCTCTAGCATGAAGTCAGACAA |
| >GS_C3z |
| AACCAATGGCCTCCTGAACGTGCGTTGCACTCTTGGGATTTCCTGAGAGTATGTCTGCTT |
| CAGTGCTTAACTTGCCCCAACTTTGCAAGCAGGATGTGTTTCTGCCTTGCGTTCTTATGA |
| GCTATTGCCCTCTGAGCCAATGGCTTGTTAATTGCTTGGTTCTTGCAAAATGCTTTGTGC |
| GCTGTTATTCAAGTTTCTACCTTCGTGGTTTTACTTGAGTGACGCTGCTCATGCTTGCAA |
| CTGCTGGGATGCAGGTGCATGCCTCTAGCATGAAGTCAGACAA |
| >GS_C3.2 |
| AACCAATGGCCTCCTGAACGTGCGTTGCACTCTTGGGATTTCCTGAGAGTATGTCTGCTT |
| CAGTGCTTAACTTGCCCCAACTTTGCAAGCAGATGTGTTTCTGCCTTGCGTTCTTATGAG |
| CTATTGCCCTCTGAGCCAATGGCTTGTTAATTGCTTGGTTCTTGCAAAATGCTTTGCGCG |
| CTGTTATTCAAGTTTCTACCTTCGTGGTTTTACTTGAGTGACGCTGCTCATGCTTGCAAC |
| CGCTGGGATGCAGGTGCATGCCTCTAGCATGAAGTCAGACAA |
| >GS_C3.7 |
| AACCAATGGCCTCCTGAACGTGCGTTGCACTCTTGGAATTTCCTGAGAGTATGTCTGCTT |
| CAGTGCTTAACTTGCCCCAACTTTGCAAGCAGGATGTGTTTCTGCCTTGCGTTCTTATGA |
| GCTATTGCCCTCTGAGCCAATGGCTTGTTAATTGCTTGGTTCTTGCAAAATGCTTTGCGC |
| GCTGTTATTCAAGTTTCTACCTTCGTGGTTTTACTTGAGTGACGCTGCTCATGCTTGCAA |
| CCGCTGGGATGCAGGTGCATGCCTCTAGCATGAAGTCAGACAA |
| >GS_C3.8 |
| AACCAATGGCCTCCTGAACGTGCGTTGCACTCTTGGGATTTCCTGAGAGTATGTCTGCTT |
| CAGTGCTTAACTTGCCCCAACTTTGCAAGCAGGATGTGTTTCTGCCTTGCGTTCTTATGA |
| GCTATTGCCCTCTGAGCCAATGGCTTGTTAATTGCTTGGTTCTTGCAAAATGCTTTGCGC |
| GCTGTTATTCAAGTTTCTATCTTCGTGGTTTTACTTGAGTGACGCTGCTCATGCTTGCAA |
| CCGCTGGGATGCAGGTGCATGCCTCTAGCATGAAGTCAGACAA |
| >GS_C3.9 |
| AACCAATGGCCTCCTGAACGTGCGTTGCACTCTTGGGATTTCCTGAGAGTATGTCTGCTT |
| CAGTGCTTAACTTGCCCAACTTTGCAAGCAGGATGTGTTTCTGCCTTGCGTTCTTATGAG |
| CTATTGCCCTCTGAGCCAATGGCTTGTTAATTGCTTGGTTCTTGCAAAATGCTTTGCGCG |
| CTGTTATTCAAGTTTCTATCTTCGTGGTTTTACTTGAGTGACGCTGCTCATGCTTGCAAC |
| CGCTGGGAGCAGGTGCATGCCTCTAGCATGAAGTCAGACAA |
| >GS_C3.10 |
| AACCAATGGCCTCCTGAACGTGCGTTGCACTCTTGGGATTTCCTGAGAGTATGTCTGCTT |
| CAGTGCTTAACTTGCCCCAACTTTGCAAGCAGGATGTGTTTCTGCCTTGCGTTCTTATGA |
| GCTATTGCCCTCTGAGCCAATGGCTTGTTAATTGCTTGGTTCTTGCAAAATGCTTTGCGC |
| GCTGTTATTCAAGTTTCTACCTTCGTGGTTTTACTTGAGTGACGCTGCTCATGCTTGCAA |
| CTGCTGGGATGCAGGTGCATGCCTCTAGCATGAAGTCAGACAA |
| >GS_C3.11 |
| AACCAATGGCCTCCTGAACGTGCGTTGCACTCTTGGGATTTCCTGAGAGTATGTCTGCTT |
| CAGTGCTTAACGTGCCCCAACTTTGCAAGCAGGATGTGTTTCTGCCTTGCGTTCTTATGA |
| GCTATTGCCCTCTGAGCCAATGGCTTGTTAATTGCTTGGTTCTTGCAAAATGCTTTGCGC |
| GCTGTTATTCAAGTTTCTACCTTCGTGGTTTTACTTGAGTGACGCTGCTCATGCTTGCAA |
| CCGCTGGGATGCAGGTGCATGCCTCTAGCATGAAGTCAGACAA |
| >GS_C3.12 |
| AACCAATGGCCTCCTGAACGTGCGTTGCACTCTTGGGATTTCCTGAGAGTATGTCTGCTT |
| CAGTGCTTAACTTGCCCCAACTTTGCAAGCAGGATGTGTTTCTGCCTTGCGTTCTTATGA |
| GCTATTGCCCTCTGAGCCAATGGCTTGTGAATTGCTTGGTTCTTGCAAAATGCTTTGCGC |
| GCTGTTATTCAAGTTTCTACCTTCGTGGTTTTACTTGAGTGACGCTGCTCATGCTTGCAA |
| CCGCTGGGATGCAGGTGCATGCCTCTAGCATGAAGTCAGACAA |
| >GS_C3.14 |
| AACCAATGGCCTCCTGAACGTACGTTGCACTCTTGGGATTTCCTGAGAGTATGTCTGCTT |
| CAGTGCTTAACTTGCCCCAACTTTGCAAGCAGGATGTGTTTCTGCCTTGCGCTCTTATGA |
| GCTATTGCCCTCTGAGCCAATGGCTTGTTAATTGCTTGGTTCTTGCAAAATGCTTTGCGC |
| GCTGTTATTCAAGTTTCTACCTTCGTGGTTTTACTTGAGTGACGCTGCTCATGCTTGCAA |
| CCGCTGGGATGCAGGTGCATGCCTCTAGCATGAAGTCAGACAA |
| >GS_C4 |
| AACCAATGGCCTCCTGAACGTGCGTTGCACTCTTGGGATTTCCTGAGAGTATGTTTGCTT |
| CAGTGCTTAACTTGCCCCAACTTTGCAAGCAGGATGTGTTTCTGCCTTGCGTTCTTATGA |
| GCTATTGCCCTCTGAGCCAATGGCTTGTGAATTGCTTGGTTCTTGCAAAATGCTTTGCGC |
| GCTGTTATTCAGGTTTCTACCTTCGTGGTTTTACTTGAGTGACGCTGCTCATGCTTGCAA |
| CCGCTGGGATGCAGGTGCATGCCTCTAGCATGAAGTCAGACAA |
| >GS_C5 |
| AACCAATGGCCTCCTGAACGTGCGTTGCACTCTTGGGATTTCCTGAGAGTATGTCTTCTT |
| CAGTGCTTAACTTGCCCCAACTTTGCAAGCAGGATATGTTTCTGCCTTGCGTTCTTATGA |
| GCTATTGCCCTCTGCGCCAATGGCTTGTTAATTGCTTGGTTCTTGCAAAATGCTTTGCGC |
| GCTGTTATTCAAGTTTCTACCCTCGCGGTTTTACTTGAGTGACGCTGCTCATGCTTGCAA |
| CCGCTGGGATGCAGGTGCATGCCTCTAGCATGAAGTCAGACAA |
| >GS_C6 |
| AACCAATGGCCTCCTGAACGTGCGTTGCACTCTTGGGATTTCCTGAGAGTATGTCTGCTT |
| CAGTGCTTACCTTGCCCCAACTTTGCAAGCAGGATGTGTTTCTGCCTTGCGTGCTTATGA |
| GCTATTGCCCTCTGAGCCAATGGCTTGTTAATTGCTTGGTTCTTGCAAAATGCTTTGCGC |
| GCTGTTATTCAAGTTTCTACCTTCGTGGTTTTACTTGAGTGACGCTGCTCATGCTTGCAA |
| CCGCTGGGATGCAGGTGCATGCCTCTAGCATGAAGTCAGACAA |
| >GS_C7 |
| AACCAATGGCCTCCTGAACGTGCGTTGCACTCTTGGGATTTCCTGAGAGTATGTCTGCTT |
| CAGTGCTTAACTTGCCCCAACTTTGCAAGCAGGATGTGTTTCTGCCTTGTGTTCTTATGA |
| GCTATTGCCCTCTGAGCCAATGGCTTGTTAATTGCTTGGTTCTTGCAAAATGCTTTGCGC |
| GCTGTTATTCAAGTTTCTACCTTCGTGGTTTTACTTGAGTGACGCTGCTCATGCTTGCAA |
| CCGCTGGGATGCAGGTGCATGCCTCTAGCATGAAGTCAGACAA |
| >GS_C8 |
| AACCAATGGCCTCCTGAACGTGCGTTGCACTCTTGGGATTTCCTGAGAGTATGTCTGCTT |
| CAGTCCTTAACTTGCCCCAACTTTGCAAGCAGGATGTGTTTCTGCCTTGCGTTCTTATGA |
| GCTATTGCCCTCTGAGCCAATGGCTTGTGAATTGCTTGGTTCTTGCAAAATGCTTTGCGC |
| GCTGTTATTCAGGTTTCTACCTTCGTGGTTTCACTTGAGTGACGCTGCTCATGCTTGCAA |
| CCGCTGGGATGCAGGTGCATGCCTCTAGCCTGAAGTCAGACAA |
| >GS_C8a |
| AACCAATGGCCTCCTGAACGTGCGTTGCACTCTTGGGATTTCCTGAGAGTATGTCTGCTT |
| CAGTCCTTAACTAGCCCCAACTTTGCAAGCAGGATGTGTTTCTGCCTTGCGTTCTTATGA |
| GCTATTGCCCTCTGAGCCAATGGCTTGTGAATTGCTTGGTTCTTGCAAAATGCTTTGCGC |
| GCTGTTATTCAGGTTTCTACCTTCGTGGTTTCACTTGAGTGACGCTGCTCATGCTTGCAA |
| CCGCTGGGATGCAGGTGCATGCCTCTAGCCTGAAGTCAGACAA |
| >GS_C8b |
| AACCAATGGCCTCCTGAACGTGCGTTGCACTCTTGGGATTTCCTGAGAGTGTGTCTGCTT |
| CAGTCCTTAACTTGCCCCAACTTTGCAAGCAGGATGTGTTTCTGCCTTGCGTTCTTATGA |
| GCTATTGCCCTCTGAGCCAATGGCTTGTGAATTGCTTGGTTCTTGCAAAATGCTTTGCGC |
| GCTGTTATCAGGTTTCTACCTTCGTGGTTTCACTTGAGTGACGCTGCTCATGCTTGCAAC |
| CGCTGGGATGCAGGTGCATGCCTCTAGCCTGAAGTCAGACAA |
| >GS_C8c |
| AACCAATGGCCTCCTGAACGTGCGTTGCACTCTTGGGATTTCCTGAGAGTATGTCTGCTT |
| CAGTCCTTAACTTGCCCCAACTTTGCAAGCAGGATGTGTTTCTGCCCTGCGTTCTTATGA |
| GCTATTGCCCTCTGAGCCAATGGCTTGTGAATTGCTTGGTTCTTGCAAAATGCTTTGCGC |
| GCTGTTATTCAGGTTTCTACCTTCGTGGTTTCACTTGAGTGACGCTGCTCATGCTTGCAA |
| CCGCTGGGATGCAGGTGCATGCCTCTAGCCTGAAGTCAGACAA |
| >GS_C9 |
| AACCAATGGCCTCCTGAACGTGCGTTGCACTCTTGGGATTTCCTGAGAGTATGTCTGCTT |
| CAGTGCTTAACTTGCCCCAACTTTGCAAGCAGGATGTGTTTCTGCCTTGCATTCCTATGA |
| GCTATTGCCCTCTGAGCCAATGGCTTGTGAATTGCTTGGTTCTTGCAAAATGCTTTGCGC |
| GCTGTTATTCAGGTTTCTACCTTCGTGGTTTTACTTGAGTGACGCTGCTCATGCTTGCAA |
| CCGCTGGGATGCAGGTGCATGCCTCTAGCATGAAGTCAGACAA |
| >GS_C10a |
| AACCAATGGCCTCCTGAACGTGCGTTGCACTCTTGGGATTTCCTGAGAGTATGTCTGCTT |
| CAGTGCTTAACTTGCCCCAACTTTGCAAGCAGGATGTGTTTCTGCCTTGCGTTCTTATGA |
| GCTATTGCCCTCTGCGCCAATGGCTTGTGAATTGCTTGGTTCTTGCAAAATGCTTTGCCC |
| GCTGTTATTCAGGTTTCTACCTTCGTGGTTTTACTTGAGTGACGCTGCTCATGCTTGCAA |
| CCGCTGGGATGCAGGTGCATGCCTCTAGCATGAAGTCAGACAA |
| >GS_C11 |
| AACCAATGGCCTCCTGAACGTGCGTTGCACTCTTGGGATTTCCTGAGAGTATGTCTGCTT |
| CAGTGCTTAACTTGCCCCAACTTTGCAAGCAGGATGTGTTTCTGCCTTGCGTTCTTATGA |
| GCTATTGCCCTCTGAGCCAATGGCTTGTTAATTGCTTGGTTCTTGCAAAATGCTTTGCGC |
| GATGTTATTCAAGTTTCTACCTTCGTGGTTTTACTTGAGTGACGCTGCTCATGCTTGCAA |
| CCGCTGGGATGCAGGTGCATGCCTCTAGCATGAAGTCAGACAA |
| >GS_C12 |
| AACCAATGGCCTCCTGAACGTGCGTTGCACTCTTGGGATTTCCTGAGAGTATGTCTCTGC |
| TTCAGTGCTTAACTTGCCCCAACTTTGCAAGCAGGATGTGTTTCTGCCTTGTGTTCTTAT |
| GAGCTATTGCCCTCTGAGCCAATGGCTTGTTAATTGCTTGGTTCTTGCAAAATGCTTTGC |
| GCGCTGTTATTCAAGTTTCTACCTTCGTGGTTTTACTTGAGTGACGCTGCTCATGCTTGC |
| AACCGCTGGGATGCAGGTGCATGCCTCTAGCATGAAGTCAGACAA |
| >GS_C10=C13 |
| AACCAATGGCCTCCTGAACGTGCGTTGCACTCTTGGGATTTCCTGAGAGTATGTCTGCTT |
| CAGTGCTTAACTTGCCCCAACTTTGCAAATGCAAGCAGGATGTGTTTCTGCCTTGCGTTC |
| TTATGAGCTATTGCCCTCTGAGCCAATGGCTTGTGAATTGCTTGGTTCTTGCAAAATGCT |
| TTGCCCGCTGTTATTCAGGTTTCTACCTTCGTGGTTTTACTTGAGTGACGCTGCTCATGC |
| TTGCAACCGCTGGGATGCAGGTGCATGCCTCTAGCATGAAGTCAGACAA |
| >GS_C14 |
| AATCAATGGCCTCCTGAACGTGCGTTGCACTCTTGGGATTTCCTGAGAGTATGTCTGCTT |
| CAGTGCTTAACTTGCCCCAACTTTGCAAGCAGGATGTGTTCTGCCTTGCGTTCTTATGAG |
| TTATTGTCCTCTGAGCCAATGGCTTGTGAATTGCTTGGTTCTTGCAAAATGCTTTGCGCG |
| CTGTTATTCAGGTTTCTACCTTCGTGGTTTTACTTGAGTGACGCTGCTCATGCTTGCAAC |
| CGCTGGGATGCAGGTGCATGCCTCTAGCATGAAGTCAGACAA |
| >GS_C15a |
| AACCAATGGCCTCCTGAACGTGCGTTGCACCCTTGGGATTTCCTGAGAGTATGTCTGCTT |
| CAGTGCTTAACTTGCCCCAACTTTGCAAGCAGGATGTGTTTCTGCCTTGCGTTCTTATGA |
| GCTATTGCCTTCTGCGCCAATGGCTTGTTAATTGCTTGGTTCTTGCAAAATGCTTTGCGC |
| GCTGTTATTCAAGTTTCTACCTTCGTGGTTTTACTTGAGTGACGCTGCTCATGCTTGCAA |
| CCGCTGGGATGCAGGTGCATGCCTCTAGCATGAAGTCAGACAA |
| >GS_C15b |
| AACCAATGGCCTCCTGAACGTGCGTTGCACCCTTGGGATTTCCTGAGAGTCTGTCTGCTT |
| CAGTGCTTAACTTGCCCCAACTTTGCAAGCAGGATGTGTTTCTGCCTTGCGTTCTTATGA |
| GCTATTGCCTTCTGCGCCAATGGCTTGTTAATTGCTTGGTTCTTGCAAAATGCTTTGCGC |
| GCTGTTATTCAAGTTTCTACCTTCGCGGTTTTACTTGAGTGACGCTGCTCATGCTTGCAA |
| CCGCTGGGATGCAGGTGCATGCCTCTAGCATGAAGTCAGACAA |
| >GS_C15c |
| AACCAATGGCCTCCTGAACGTGCCTTGCACCCTTGGGATTTCCTGAGAGTATGTCTGCTT |
| CAGTGCTTAACTTGCCCCAACTTTGCAAGCAGGATGTGTTTCTGCCTTGCGTTCTTATGA |
| GCTATTGACTTCTGCGCCAATGGCTTGTTAATTGCTTGGTTCTTGCAAAATGCTTTGCGC |
| GCTGTTATTCAAGTTTCTACCTTCGCGGTTTTACTTGAGTGACGCTGCTCATGCTTGCAA |
| CCGCTGGGATGCAGGTGCATGCCTCTAGCATGAAGTCAGACAA |
| >GS_C15d |
| AACCAATGGCCTCCTGAACGTGCCTTGCACCCTTGGGATTTCCTGAGAGTATGTCTGCTT |
| CAGTGCTTAACTTGCCCCAACTTTGCAAGCAGGATGTGTTTCTGCCTTGCGTTCTTATGA |
| GCTATTGACTTCTGCGCCAATGGCTTGTTAATTGCTTGGTTCTTGCAAAATGCTTTGCGC |
| ACTGTTATTCAAGTTTCTACCTTCGCGGTTTTACTTGAGTGACGCTGCTCATGCTTGCAA |
| CCGCTGGGATGCAGGTGCATGCCTCTAGCATGAAGTCAGACAA |
| >GS_C15e |
| AACCAATGGCCTCCTGAACGTGCGTTGCACCCTTGGGATTTCCTGAGAGTATGTCTGCTT |
| CAGTGCTTAACTTGCCCCAACTTTGCAAGCAGGATGTGTTTCTGCCTTGCGTTCTTATGA |
| GCTATTGCCTTCTGCGCCAATGGCTTGTTAATTGCTTGGTTCTTGCAAAATGCTTTGCGC |
| GCTGTTATTCAAGTTTCTACCTTCGCGGTTTTACTTGAGTGACGTGCTCATGCTTGCAAC |
| CGCAGGGATGCAGGTGCATGCCTCTAGCATGAAGTCAGACAA |
| >GS_C15f |
| AACCAATGGCCTCCTGAACGTGCGTTGCACCCTTGGGATTTCCTGAGAGTATGTCTGCTT |
| CAGTGCTTAACTTGCCCCAACTTTGCAAGCAGGATGTGTTTCTGCCTTGCGTTCTTATGA |
| GCTATTGCCTTCTGCGCCCAATGGCTTGTTAATTGCTTGGTTCTTGCAAAATGCTTTGCG |
| CGCTGTTATTCAAGTTTCTACCTTCGCGGTTTTACTTGAGTGACGCTGCTCATGCTTGCA |
| ACCGCTGGGATGCAGGTGCATGCCTCTAGCATGAAGTCAGACAA |
| >GS_C15g |
| AACCAATGGCCTCCTGAACGTGCGTTGCACCCTTGGGATTTCCTGAGAGTATGTCTGCTT |
| CAGTGCTTAACTTGCCCTAACTTTGCAAGCAGGATGTGTTTCTGCCTTGCGTTCTTATGA |
| GCTATTGCCTTCTGCGCCAATGGCTTGTTAATTGCTTGGTTCTTGCAAAATGCTTTGCGC |
| GCTGTTATTCAAGTTTCTACCTTCGCGGTTTTACTTGAGTGACGCTGCTCATGCTTGCAA |
| CCGCTGGGATGCAGGTGCATGCCTCTAGCATGAAGTCAGACAA |
| >GS_C15h |
| AATCAATGGCCTCCTGAACGTGCGTTGCACCCTTGGGATTTCCTGAGAGTATGTCTGCTT |
| CAGTGCTTAACTTGCCCCAACTTTGCAAGCAGGATGTGTTTCTGCCTTGCGTTCTTATGA |
| GCTATTGCCTTCTGCGCCAATGGCTTGTTAATTGCTTGGTTCTTGCAAAATGCTTTGCGC |
| GCTGTTATTCAAGTTTCTACCTTCGCGGTTTTACTTGAGTGACGCTGCTCATGCTTGCAA |
| CCGCTGGGATGCAGGTGCATGCCTCTAGCATGAAGTCAGACAA |
| >GS_C15i |
| AACCAATGGCCTCCTGAACGTGCGTTGCACCCTTGGGATTTCCTGAGAGTATGTCTGCTT |
| CAGTGCTTAACTTGCCCCAACTTTGCAAGCAGGATGTGTTTCTGCCTTGCGTTCTTATGA |
| GCTATTGCCTTCTGCGCCAATGGCTTGTTAATTGCTCGGTTCTTGCAAAATGCTTTGCGC |
| GCTGTTATTCAAGTTTCTACCTTCGCGGTTTTACTTGAGTGACGCTGCTCATGCTTGCAA |
| CCGCTGGGATGCAGGTGCATGCCTCTAGCATGAAGTCAGACAA |
| >GS_C15j |
| AACCAATGGCCTCCTGAACGTGCGTTGCACCCTTGGGATTTCCTGAGAGTATGTCTGCTT |
| CAGTGCTTAACTTGCCCCAACTTTGCAAGCAGGATGTGTTTCTGCCTTGCGTTCTTATGA |
| GCTATTGCCTTCTGCGCCAATGGCTTGTTAATTGCTTGGTTCTTGCAAAATGCTTTGCGC |
| GCTGTTATTCAAGTTTCTACCTTCGCGGTTTTACTTGAGTGACGCTGCTCGTGCTTGCAA |
| CCGCTGGGATGCAGGTGCATGCCTCTAGCATGAAGTCAGACAA |
| >GS_C15k |
| AACCAATGGCCTCCTGAACGTGCGTTGCACCCTTGGGATTTCCTGAGAGTATGTCTGCTT |
| CAGTGCTTAACTTGCCCCAACTTTGCAAGCAGGATGTGTTTCTGCCTTGCGTTCTTATGA |
| GCTATTGCCTTCTGCGCCAATGGCTTGTTAATTGCTTGGTTCTTGCAAAATGCTTTGCGC |
| GCTGTTATTCAAGTTTCTACCTTCGCGGTTTTACTTGAGTGACGCTGCTCATGCTTGCAA |
| CCGCTGGGATGCAGGTGCATGCCTCTAGACAGAAGTCAGACAA |
| >GS_C15L |
| AACCAATGGCCTCCTGAACGTGCGTTGCACCCTTGGGATTTCCTGAGAGTATGTCTGCTT |
| CAGTGCTTAACTTGCCCCAACTTTGCAAGCAGGATGTGTTTCTGCCTTGTGTTCTTATGA |
| GCTATTGCCTTCTGCGCCAATGGCTTGTTAATTGCTTGGTTCTTGCAAAATGCTTTGCGC |
| GCTGTTATTCAAGTTTCTACCTTCGCGGTTTTACTTGAGTGACGCTGCTCATGCTTGCAA |
| CCGCTGGGATGCAGGTGCATGCCTCTAGCATGAAGTCAGACAA |
| >GS_C15m |
| AATCAATGGCCTCCTGAACGTGCGTTGCACCCTTGGGATTTCCTGAGAGTATGTTTGCTT |
| CAGTGCTTAACTTGCCCCAACTTTGCAAGCAGGATGTGTTTCTGCCTTGCGTTCTTATGA |
| GCTATTGCCTTCTGCGCCAATGGCTTGTTAATTGCTTGGTTCTTGCAAAATGCTTTGCGC |
| GCTGTTATTCAAGTTTCTACCTTCGCGGTTTTACTTGAGTGACGCTGCTCATGCTTGCAA |
| CCGCTGGGATGCAGGTGCATGCCTCTAGCATGAAGTCAGACAA |
| >GS_C15.1 |
| AACCAATGGCCTCCTGAACGTGCGTTGCACCCTTGGGATTTCCTGAGAGTATGTCTGCTT |
| CAGTGCTTAACTTGCCCCAACTTTGCAAGCAGGATGTGCTTCTGCCTTGCGTTCTTATGA |
| GCTATTGCCTTCTGCGCCAATGGCTTGTTAATTGCTTGGTTCTTGCAAAATGCTTTGCGC |
| GCTGTTATTCAAGTTTCTACCTTCGCGGTTTTACTTGAGTGACGCTGCTCATGCTTGCAA |
| CCGCTGGGATGCAGGTGCATGCCTCTAGCATGAAGTCAGACAA |
| >GS_C15.2_(type_1) |
| AACCAATGGCTTCCTGAACGTGCGTTGCACCCTTGGGATTTCCTGAGAGTATGTCTGCTT |
| CAGTGCTTAACTTGCCCCAACTTTGCAAGCAGGATGTGTTTCTGCCTTGCGTTCTTATGA |
| GCTATTGCCTTCTGCGCCAATGGCTTGTTAATTGCTTGGTTCTTGCAAAATGCTTTGCGC |
| GCTGTTATTCAAGTTTCTACCTTCGCGGTTTTACTTGAGTGACGCTGCTCATGCTTGCAA |
| CCGCTGGGATGCAGGTGCATGCCTCTAGCATGAAGTCAGACAA |
| >GS_C15.2_(type_2) |
| AACCAATGGCCTCCTGAACGTGCGTTGCACCCTTGGGATTTCCTGAGAGTATGTCTGCTT |
| CAGTGCTTAACTTGCCCCAACTTTGCAAGCAGGATGTGTTTCTGCCTTGCGTGCTTATGA |
| CCTATTGCCTTCTGCGCCAATGGCTTGTTAATTGCTTGGTTCTTGCAAAATGCTTTGCGC |
| GCTGTTATTCAAGTTTCTACCTTCGCGGTTTTACTTGAGTGACGCTGCTCATGCTTGCAA |
| CCGCTGGGATGCAGGTGCATGCCTCTAGCATGAAGTCAGACAA |
| >GS_C15.3 |
| AACCAATGGCTTCCTGAACGTGCGTTGCACCCTTGGGATTTCCTGAGAGTATGTCTGCTT |
| CAGTGCTTAACTTGCCCCAACTTTGCAAGCAGGATGTGTTTCTGCCTTGCGTTCTTATGA |
| GCTATTGCCTTCTGCGCCAATGGCTTGTTAATTGCTTGGTTCTTGCAAAATGCTTTGCGC |
| GCTGTTATTCAAGTTTCTACCTTCGTGGTTTTACTTGAGTGACGCTGCTCATGCTTGCAA |
| CCGCTGGGATGCAGGTGCATGCCTCTAGCATGAAGTCAGACAA |
| >GS_C15.4 |
| AAGCAATGGCCTCCTGAACGTGCGTTGCACCCTTGGGATTTCCTGAGAGTATGTCTGCTT |
| CAGTGCTTAACTTGCCCCAACTTTGCAAGCAGGATGTGTTTCTGCCTTGCGTTCTTATGA |
| GCTATTGCCTTCTGCGCCAATGGCTTGTTAATTGCTTGGTTCTTGCAAAATGCTTTGCGC |
| GCTGTTATTCAAGTTTCTACCTTCGTGGTTTTACTTGAGTGACGCTGCTCATGCTTGCAA |
| CCGCTGGGATGCAGGTGCATGCCTCTAGCATGAAGTCAGACAA |
| >GS_C15.5 |
| AAGCAATGGCCTCCTGAACGTGCGTTGCACCCTTGGGATTTCCTGAGAGTATGTCTGCTT |
| CAGTGCTTAACTTGCCCCAACTTTGCAAGCAGGATGCGTTTCTGCCTTGCGTTCTTATGA |
| GCTATTGCCTTCTGCGCCAATGGCTTGTTAATTGCTTGGTTCTTGCAAAATGCTTTGCGC |
| GCTGTTATTCAAGTTTCTACCTTCGTGGTTTTACTTGAGTGACGCTGCTCATGCTTGCAA |
| CCGCTGGGATGCAGGTGCATGCCTCTAGCATGAAGTCAGACAA |
| >GS_C15.6 |
| AAGCAATGGCCTCCTGAACGTGCGTTGCACCCTTGGGATTTCCTGAGAGTATGTCTGCTT |
| CAGTGCTTAACTTGCCCCAACTTTGCAAGCAGGATGTGTTTCTGCCTTGCGTTCTTATGA |
| GCTATTGCCTTCTGCGCCAATGGCTTGTTAATTGCTTGGTTCTTGCAAAATGCTTTGCGC |
| GCTGTTATTCAAGTTTCTACCTTCGCGGTTTTACTTGAGTGACGCTGCTCATGCTTGCAA |
| CCGCTGGGATGCAGGTGCATGCCTCTAGCATGAAGTCAGACAA |
| >GS_C15.7 |
| AACCAATGGCCTCCTGAACGTGCGTTGCACCCTTGGGATTTCCTGAGAGTATGTCTGCTT |
| CAGTGCTTAACTTGCCCCAACTTTGCAAGCAGGATGTGTTTCTGCCTTGCGTTCTTATGA |
| GCTATTGCCTTCTGCGCCAATGGCTTGTTAATTGCTTGGTTCTTGCAAAATGCTTTGCGC |
| GCTGTTATTCAAGTTTCTACCTTCGCGGTTTTACTTGAGTGACGCTGCTCATGCTTGCAA |
| CCGCTGGGATGCAGGTGCATGCCTCTAGCATGAAGTCAGAGAA |
| >GS_C15.8 |
| AACCAATGGCCTCCTGAACGTGCGTTGCACCCTTGGGATTTCCTGAGAGTATGTCTGCTT |
| CAGTACTTAACTTGCCCCAACTTTGCAAGCAGGATGTGTTTCTGCCTTGCGTTCTTATGA |
| GCTATTGCCTTCTGCGCCAATGGCTTGTTAATTGCTTGGTTCTTGCAAAATGCTTTGCGC |
| GCTGTTATTCAAGTTTCTACCTTCGCGGTTTTACTTGAGTGACGCTGCTCATGCTTGCAA |
| CCGCTGGGATGCAGGTGCATGCCTCTAGCATGAAGTCAGACAA |
| >GS_C15.9 |
| AACCAATGGCCTCCTGAACGTGCGTTGCACCCTTGGGATTTCCTGAGAGTATGTCTGCTT |
| CAGTGCTTAACTTGCCCCAACTTTGCAAGCAGGATGTGTTTCTGCCTTGCGTTCTTATGA |
| GCTATTGCCTTCTGCGCCAATGGCTTGTTAATTGCTTGGTTCTTGCAAAATGCTTTGCGC |
| GCTGTTATTCAAGTTTCTACCTTTGCGGTTTTACTTGAGTGACGCTGCTCATGCTTGCAA |
| CCGCTGGGATGCAGGTGCATGCCTCTAGCATGAAGTCAGACAA |
| >GS_KB1 |
| AACCAATGGCGAAGGTGTGTTGCACCCTTGGGATTTCCTGAGAGTATGTCTGCTTCAGTG |
| CTTAACTTGCCCCAACTTTGCAAGCAGGATGTGTTTCTGCCTTGCGTTCTTATGAGCTAT |
| TGCCTTCTGCGCCAATGGCTTGGTAATTGCTTGGTTCTTGCAGAACGCTTTGCGTGCTGT |
| TATCATTTTCTACCTTCGCGGTTTTACTTGAGTGACGCTGCTCATGCTTGCAACCGCTGG |
| GATGCAGGTGCATGCCTCTAGCATGAAGTCAGACAA |
| >GS_KB2 |
| AACCAATGGCGAAGGTGTGTTGCACCTTTGGGATTTCCTGAGAGTATGTCTGCTTCAGTG |
| CTTAACTTGCCCCAACTTTGCAAGCAGGATGTGTTTCTGCCTTGCGTTCTTATGAGCTAT |
| TGCCTTCTGCGCCAATGGCTTGGTAATTGCTTGGTTCTTGCAGAACGCTTTGCGTGCTGT |
| TATTCCAGTTTCTACCTTCGCGGTTTTACTTGAGTGACGCTGCTCATGCTTGCAACCGCT |
| GGGATGCAGGTGCATGCCTCTAGCATGAAGTCAGACAA |
| >GS_KB3 |
| AACCAATGGCGAACGTGTGTTGCACCCTTGGGATTTCCTGAGAGTATGTCTGCTTCAGTG |
| CTTAACTTGCCCCAACTTTGCAAGCAGGATGTGTTTCTGCCTTGCGTTCTTATGAGCTAC |
| TGCCTTCTGCGCCAATGGCTTGGTAATTGCTTGGTTCTTGCAAAACGCTTTGCGTGCTGT |
| TATTCCAGTTTCTACCTTCGCGGTTTTACTTGAGTGACGCTGCTCATGCTTGCAACCGCT |
| GGGATGCAGGTGCATGCCTCTAGCATGAAGTCAGACAA |
| >GS_KB4 |
| AACCAATGGCGAACGTGTGTTGCACCCTTGGGATTTCCTGAGAGTATGTCTGCTTCAGTG |
| CTTAACTTGCCCCAACTTTGCAAGCAGGATGTGTTTCTGCCTTGCGTTCTTATGAGCTAC |
| TGGCTTCTGCGCCAATGGCTTGGTAATTGCTTGGTTCTTGCAAAACGCTTTGCGTGCTGT |
| TATTCCAGTTTCTACCTTCGCGGTTTTACTTGAGTGACGCTGCTCATGCTTGCAACCGCT |
| GGGATGCAGGTGCATGCCTCTAGCATGAAGTCAGACAA |
| >GS_C16 |
| AACCAATGGCCTCCTGAACGTGCGTTGCACTCTTGGGATTTCCTGAGAGTATGTCTGCTT |
| CAGTACTTAACTTGCCCCAACTTTGCAAGCAGGATGTGTTTCTGCCTTGCGTTCTTATGA |
| GCTATTGCCCTCTGAGCCAATGGCTTGTTAATTGCTTGGTTCTTGCAAACTGCTTTGCGC |
| GCTGTTATTCAAGTTTCTACCTTCGTGGTTTTACTTGAGTGACGCTGCTCATGCTTGCAA |
| CCGCTGGGATGCAGGTGCATGCCTCTAGCATGAAGTCAGACAA |
| >GS_C16a |
| AACCAATGGCCTCCTGAACGTGCGTTGCACTCTTGGGATTTCCTGAGAGTATGTCTGCTT |
| CAGTACTTAACTTGCCCCAACTTTGCAAGCAGGATGTGTTTCTGCCTTGCGTTCTTATGA |
| GCTATTGCCCTCTGAGCCAATGGCTTGTTAATTGCTTGGTTCTTGCAAACTGCTTTGCGC |
| GCTGTTATTCAAGTTTCTACCTTCGTGGTTTTACTTGAGTAACGCTGCTCATGCTTGCAA |
| CCGCTGGGATGCAGGTGCATGCCTCTAGCATGAAGTCAGACAA |
| >GS_C17a |
| AACCAATGGCCTCCTGAACGTGCGTTGCACTCTTGGGATTTCCTGAGAGTATGTCTGCTT |
| CAGCGCTTAACTTGCCCCAACTTTGCAAGCATTTCTGCCTTGCGTTCTTATGAGCTATTG |
| CCCTCTGAGTCAATGGCTTGTTAATTGCTTGGTTCTTGCAAAATGCTTTGCGCGCTGTTA |
| TTCAAGTTTCTACCTTCGTGGTTTTACTTGAGTGACGCTGCTCATGCTTGCAACCGCTGG |
| GATGCAGGTGCATGCCTCTAGCATGAAGTCAGACAA |
| >GS_C17=C17.2 |
| AACCAATGGCCTCCTGAACGTGCGTTGCACTCTTGGGATTTCCTGAGAGTATGTCTGCTT |
| CAGCGCTTAACTTGCCCCAACTTTGCAAGCATTTCTGCCTTGCGTTCTTATGAGCTATTG |
| CCCTCTCTGAGCCAATGGCTTGTTAATTGCTTGGTTCTTGCAAAATGCTTTGCGCGCTGT |
| TATTCAAGTTTCTACCTTCGTGGTTTTACTTGAGTGACGCTGCTCATGCTTGCAACCGCT |
| GGGATGCAGGTGCATGCCTCTAGCATGAAGTCAGACAA |
| >GS_C18 |
| AACCAATGGCCTCCTGAACGTGCGTTGCACTCTTGGGATTTCCTGAGAGTATGTCTGCTT |
| CAGTGCTTAACTTGCCCCAACTTTGCAAGCAGGATGTGTTTCTGCCTTGCGTTCTTATGA |
| GCTATTGCCCTCTGAGCCAATGGCTTGTTAATTGCTTGGTTCTTGCAAAATGCGTTGCGC |
| GCTGTTATTCAGGTTTCTACCTTCGTGGTTTTACTTGAGTGACGCTGCTCATGCTTGCAA |
| CCGCTGGGATGCAGGTGCATGCCTCTAGCATGAAGTCAGACAA |
| >GS_C19 |
| AACCAATGGCCTCCTGAACGTGCGTTGCACCCTTGGGATTTCCTGAGAGTATGTCTGCTT |
| CAGTGCTTAACTTGCTCCAACTTTGCAAGCAGGATGTGTTTCTGCCTTGCGTTCTTATGA |
| GCTATTGCCTTCTGGCCAATGGCTTGTTAATTGCTTGGTTCTTGCAAAATGCTTTGCGCG |
| CTGTTATTCAAGTTTCTACCTTCGCGGTTTTACTTGAGTGACGCTGCTCATGCTTGCAAC |
| CGCTGGGATGCAGGTGCATGCCTCTAGCATGAAGTCAGACAA |
| >GS_C20 |
| AACCAATGGCCTCCTGAACGTGCGTTGCACTCTTGGGTTTTCCTGAGAGTATGTCTGCTT |
| CAGTGCTTAACTTGCCCCAACTTTGCAAGCAGGATGTGTTTCTGCCTTGCGTTCTTATGA |
| GCTATTGCTCTCTGAGCCAATGGCTTGTCAATTGCTTGGTTCTTGCAAAATGCTTTGCGC |
| GCTGTTATTCAAGTTTCTACCTTCGTGGTTTTACTTGAGTGACGCTGCTCATGCTTGCAA |
| CCGCTGGGATGCAGGTGCATGCCTCTAGCATGAAGTCAGACAA |
| >GS_C21.11 |
| AACCAATGGCCTCCTGAACGTGCGTTGCACTCTTGGGATTTCCTGAGAGTATGTCTGCTT |
| CAGTGCTTAACTTGCCCCAACTTTGCAAGCATTTCTGCCTTGCGTTTTTATGAGCTATTG |
| CCCTCTGAGCCAATGGCTTGTTAATTGCTTGGTTCTTGCAAAATGCTTTGCGCGCTGTTA |
| TTCAAGTTTCTACCTTCGTGGTTTTACTTGAGTGACGCTGCTCATGCTTGCAACCGCTGG |
| GATGCAGGTGCATGCCTCTAGCATGAAGTCAGACAA |
| >GS_C21.12 |
| AACCAATGGCTTCGTGAACGTGCGTTGCACTCTTGGGATTTCCTGAGAGTATGTCTGCTT |
| CAGTGCTTAACTTGCCCCAACTTTGCAAGCATTTCTGCCTTGCGTTCTTATGAGCTATTG |
| CCCTCTGAGCCAATGGCTTGTTAATTGCTTGGTTCTTGCAAAATGCTTTGCGCGCTGTTA |
| TTCAAGTTTCTACCTTCGTGGTTTTACTTGAGTGACGCTGCTCATGCTTGCAACCGCTGG |
| GATGCAGGTGCATGCCTCTAGCATGAAGTCAGACAA |
| >GS_C21.13 |
| AACCAATGGCTTCGTGAACGTGCGTTGCACTCTTGGGATTTCCTGAGAGTATGTCTGCTT |
| CAGTGCTTAACTTGCCCCAACTTTGCAAGCATTTCTGCTTTGCGTTCTTATGAGCTATTG |
| CCCTCTGAGCCAATGGCTTGTTAATTGCTTGGTTCTTGCAAAATGCTTTGCGCGCTGTTA |
| TTCAAGTTTCTACCTTCGTGGTTTTACTTGAGTGACGCTGCTCATGCTTGCAACCGCTGA |
| GATGCAGGTGCATGCCTCTAGCATGAAGTCAGACAA |
| >GS_C21.14 |
| AACCAATGGCCTCCTGAACGTGCGTTGCACTCTTGGGATTTCCTGAGAGTATGTCTGCTT |
| CAGTGCTTAACTTGCCCCAACTTTGCAAGCATTTCTGCCTTGCGTTCTTATGAGCTATTG |
| CCCTCTGAGCCAATGGCTTGTTAATTGCTTGGTTCTTGCAAAATGCTTTGCGCGCTGTTA |
| TTCAAGTTTCTACCTTCGTGGTTTTACTTGAGTGACGCTGCTCATGCTTGCAACTGCTGG |
| GATGCAGGTGCATGCCTCTAGCATGAAGTCAGACAA |
| >GS_C21.16 |
| AACCAATGGCCTCCTGAACGTGCGTTGCACTCTTGGGATTTCCTGAGAGTATGTCTGCTT |
| CAGTGCTTAACTTGCCCCAACTTTGCAAGCATTTCTGCCTTGCGTTCTTATGAGCTATTG |
| CCCTCTGAGCCAATGGCTTGTTAATTGCTTGGTTCTTGCAAAATGCTTTGCGCGCTGTTA |
| TTCAAGTTTCTACCTTCGTGGTTTTACTTGAGTGACGTTGCTCATGCTTGCAACCGCTGG |
| GATGCAGGTGCATGCCTCTAGCATGAAGTCAGACAA |
| >GS_C21a |
| AACCAATGGCCTCCTGAACGTGCGTTGCACTCTTGGGATTTCCTGAGAGGATGTCTGCTT |
| CAGTGCTTAACTTGCCCCAACTTTGCAAGCATTTCTGCCTTGCGTTCTTATGAGCTATTG |
| CCCTCTGAGCCAATGGCTTGTTAATTGCTTGGTTCTTGCAAAATGCTTTGCGCGCTGTTA |
| TTCAAGTTTCTACCTTCGTGGTTTTACTTGAGTGACGCTGCTCATGCTTGCAACCGCTGG |
| GATGCAGGTGCATGCCTCTAGCATGAAGTCAGACAA |
| >GS_C22 |
| AACCAATGGCCTCCTGAACGTGCGTTGCACTCTTGGGATTTCCTGAGAGTATGTCTGCTT |
| CAGTGCTTAACTTGCCCCAACTTTGCAAGCAGGATGTGTTTCTGCCTTGCGTTCTTATGA |
| GCTATTGCCCTCTGAGCCAATGGCTTGTTAATTGCTTGGTTCTTGCAAAATGCTTTGCGC |
| GCTGTTATTCAAGTTTCTACCTTCGTGGTTTTACTTGAGTCTCTCATGCTTGCAACCGCT |
| GGGATGCAGGTGCATGCCTCTAGCATGAAGTCAGACAA |
| >GS_C22a |
| AACCAATGGCCTCCTGAACGTGCGTTGCACTCTTGGGATTTCCTGAGAGTATGTCTGCTT |
| CAGTGCTTAACTTGCCCCAACTTTGCAAGCAGGATGTGTTTCTGCCTTGCGTTCTTATGA |
| GCTATTGCCCTCTGAGCCAATGGCTTGTTAATTGCTTGGTTCTTGCAAAATGCTTTGCGC |
| GCTGTTATTCAAGTTTCCACCTTCGTGGTTTTACTTGAGTCTCTCATGCTTGCAACCGCT |
| GGGATGCAGGTGCATGCCTCTAGCATGAAGTCAGACAA |
| >GS_C23 |
| AACCAATGGCCTCCTGAACGTGCGTTGCACTCTTGGGATTTCCTGAGAGTATGTCTGCTT |
| CAGTGCTTAACTTGCCCCAACTTTGCAAGCAGGATGTGTGTCTGCCTTGCGTTCTTATGC |
| GCTATTGCCCTCTGAGCCAATGGCTTGTTAATTGCTTGGTTCTTGCAAAATGCTTTGCGC |
| GCTGTTATTCAGGTTTCTACCTTCGTGGTTTTACTTGAGTGACGCTGCTCATGCTTGCAA |
| CCGCTGGGATGCAGGTGCATGCCTCTAGCATGAAGTCAGACAA |
| >GS_C24 |
| AACCAATGGCCTCCTGAACGTGCGTTGCACTCTTGGGATTTCCTGAGAATATGTCTGCTT |
| CAGTGCTTAACTTGCCCCAACTTTGCAAGCAGGATGTGTTTCTGCCTTGCGTTCTTATGA |
| GCTATTGCCCTCTGAGCCAATGGCTTGTTAATTGCTTGGTTCTTGCAAAATGCTTTGCGC |
| GCTGTTATTCAGGTTTCTACCTTCGTGATTTTACTTGAGTGACGCTGCTCATGCTTGCAA |
| CCGCTGGGATGCAGGTGCATGCCTCTAGCATGAAGTCAGACAA |
| >GS_C25 |
| AATCAATGGCCTCCTGAACGTTCGTTGCACTCTTGGGATTTCCTGAGAGTATGTCTGCTT |
| CAGTGCTTAACTTGCCCCAACTTTGCAAGCAGGATGTGTTTCTGCCTTGCGCTCTTATGA |
| GTCATTGCCCTCTGAGCCAATGGCTTGTTAATTGCTTGGCTCTTGCAAAATGCTTTGCGC |
| GCTGTTATTCACGTTTCTACCTTCGTGGTTTTACTTGAGTGACACGCTGCTCATGCTTGC |
| AACCGCTGGGATGCAGGTGCATGCCTCTAGCATGAAGTCAGACAA |
| >GS_C26=C26a=C35a_(type_2) |
| AATCAATGGCCTCCTGAACGTGCGTTGCACTCTTGGGATTTCCTGAGAGTATGTCTGCTT |
| CAGCGCTTAACTTGCCCCAACTTTGCAAGCATTTCTGCCTTGCGTTCTTATGAGCTATTG |
| CCTCTCTGAGCCAATGGCTTGTTAATTGCTTGGTTCTTGCAAAATGCTTTGCGCGCTGTT |
| ATTCAAGTTTCTACCTTCGTGGTTTTACTTGAGTGACGCTGCTCATGCTTGCAACCGCTG |
| GGATGCAGGTGCATGCCTCTAGCATGAAGTCAGACAA |
| >GS_C21.b1 |
| AACCAATGGCCTCCTGAACGTGCGTTGCACTCTTGGGATTTCCTGAGAGTATGTCTGCTC |
| CAGTGCTTAACTTGCCCCAACTTTGCAAGCATTTCTGCCTTGCGTTCTTATGAGCTATTC |
| CCCTCTGAGCCAATGGCTTGTTAATTGCTTGGTTCTTGCAAAATGCTTTGCGCGCTGTTA |
| TTCAAGTTTCTACCTTCGTGGTTTTACTTGAGTGACGCTGCTCATGCTTGCAACCGCTGG |
| GATGCAGGTGCATGCCTCTAGCATGAAGTCAGACAA |
| >GS_C26.b1=C35_(type_2) |
| AACCAATGGCCTCCTGAACGTGCGTTGCACTCTTGGGATTTCCTGAGAGTATGTCTGCTT |
| CAGCGCTTAACTTGCCCCAACTTTGCAAGCATTTCTGCCTTGCGTTCTTATGAGCTATTG |
| CCTCTCTGAGCCAATGGCTTGTTAATTGCTTGGTTCTTGCAAAATGCTTTGCGCGCTGTT |
| ATTCAAGTTTCTACCTTCGTGGTTTTACTTGAGTGACGCTGCTCATGCTTGCAACCGCTG |
| GGATGCAGGTGCATGCCTCTAGCATGAAGTCAGACAA |
| >GS_C26.b2 |
| AACCAATGGCCTCCTGAACGTGCGTTGCACTCTTGGGATTTCCTGAGAGTATGTCTGCTT |
| CAGCGCTTAACTTGCCTCAACTTTGCAAGCATTTCTGCCTTGCGTTCTTATGAGCTATTG |
| CCTCTCTGAGCCAATGGCTTGTTAATTGCTTGGTTCTTGCAAAATGCTTTGCGCGCTGTT |
| ATTCAAGTTTCTACCTTCGTGGTTTTACTTGAGTGACGCTGCTCATGCTTGCAACCGCTG |
| GGATGCAGGTGCATGCCTCTAGCATGAAGTCAGACAA |
| >GS_C26.b3 |
| AATCAATGGCTTCCTGAACGTGCGTTGCACTCTTGGGATTTCCTGAGAGTATGTCTGCTT |
| CAGCGCTTAACTTGCCCCAACTTTGCAAGCATTTCTGCCTTGCGTTCTTATGAGCTATTG |
| CCTCTCTGAGCCAATGGCTTGTTAATTGCTTGGTTCTTGCAAAATGCTTTGCGCGCTGTT |
| ATTCAAGTTTCTACCTTCGTGGTTTTACTTGAGTGACGCTGCTCATGCTTGCAACCGCTG |
| GGATGCAGGTGCATGCCTCTAGCATGAAGTCAGACAA |
| >GS_C27=GS_C30_(type_1) |
| AACCAATGGCCTCCTGAACGTGCGTTGCACTCTTGGGATTTCCTGAGAGTATGTCTGCTT |
| CAGTGCTTAACTTGCCCCAACTTTGCAAGCATTTCTGCCTTGCGTTCTTATGAGCTATTG |
| CCCCTCTGAGCCGATGGCTTGTTAATTGCTTAGTTCTTGCAAAATGCTTTGCGCGCTGTT |
| ATTCAAGTTTCTACCTTCGTGGTTTTACTTGAGTGACGCTGCTCATGCTTGCAACCGCTG |
| GGATGCAGGTGCATGCCTCTAGCATGAAGTCAGACAA |
| >GS_C27.1 |
| AACCAATGGCCTCCTGAACGTGCGTTGCACTCTTGGGATTTCCTGAGAGTATGTCTGCTT |
| CAGTGCTTAACTTGCCCCAACTTTGCAAGCATTTCTGCCTTGCGTTCTTATGAGCTATTG |
| CCCCTCTGAGCCGATGGCTTGTTAATTGCTTAGTTCTTGCAAAATGCTTTGCGCGCTGTT |
| ATTCAAGTTTCTACCTTCGTGGTTTTACTTGAGTGACGCTGCTCATGCTTGCAACCGCTG |
| GGATGCAGGTGCATGCCTCTAGCATGAAGTTAGACAA |
| >GS_C28 |
| AACCAATGGCCTCCTGAACGTGCGTTGCACTCTTGGGATTTCCTGAGAGTATGTCTGCTT |
| CAGTGCTTAACTTTTGCCCCAACTTTGCAAGCAGGATGTGTTTCTGCCTTGCGTTCATAT |
| GAGCTATTGCCCTCTGAGCCAATGGCTTGTTAATTGCTTGGTTCTTGCAAAATGCTTTGC |
| GCGCTGTTATTCAAGTTTCTACCTTCGTGGTTTTACTTGAGTGACGCTGCTCATGCTTGC |
| AACCGCTGGGATGCAGGTGCATGCCTCTAGCATGAAGTCAGACAA |
| >GS_C29 |
| AACCAATGGCCTCCTGAACGTGCGTTGCACTCTTGGGATTTCCTGAGAGTATGTCTGCTT |
| CAGTGCTTAACTTGCCCCAACTTTGCAAGCAGGATGTGTTTCTGCCTTGCGTTCTTATGA |
| GCTATTGCCCTCTGAGCCAATGGCTTGTTAATTGCTTGGCTCTTGCAAAATGCTTTGCGC |
| GCTGTTATTCAAGTTTCTACCTTCGTGGTTTTACTTGAGTGACGCTGCTCATGCTTGCAA |
| CCGCTGGGATGCAGGTGCATGCCTCTAGCATGAAGTCAGACAA |
| >GS_C31d=C30_(type_2) |
| AACCAATGGCCTCCTGAACGTGCGTTGCACTCTTGGGATTTCCTGAGAGTATGTCTGCTT |
| CAGCGCTTAACTTGCCCCAACTTTGCAAGCACTCTTCTGCCTTGCGTTCTTATGAGCTAT |
| TGCCCTCTCTGAGCCAATGGCTTGTTAATTGCTTGGTTCTTGCAAAATGCTTTGCGCGCT |
| ATTATTCAAGTTTCTACCTTCGTGGTTTTACTTGAGTGACGCTGCTCATGCTTGCAACCG |
| CTGGGATGCAGGTGCATGCCTCTAGCATGAAGTCAGACAA |
| >GS_C31 |
| AACCAATGGCCTCCTGAACGTGCGTTGCACTCTTGGGATTTCCTGAGAGTATGTCTGCTT |
| CAGCGCTTAACTTGCCCCAACTTTGCAAGCACTCTTCTGCCTTGCGTTCTTATGAGCTAT |
| TGCCCTCTCTGAGCCAATGGCTTGTTAATTGCTTGGTTCTTGCAAAATGCTTTGCGCGCT |
| GTTATTCAAGTTTCTACCTTCGTGGTTTTACTTGAGTGACGCTGCTCATGCTTGCAACCG |
| CTGGGATGCAGGTGCATGCCTCTAGCATGAAGTCAGACAA |
| >GS_C31a |
| AATCAATGGCCTCCTGAACGTGCGTTGCACTCTTGGGATTTCCTGAGAGTATGTCTGCTT |
| CAGCGCTTAACTTGCCCCAACTTTGCAAGCACTCTTCTGCCTTGCGTTCTTATGAGCTAT |
| TGCCCTCTCTGAGCCAATGGCTTGTTAATTGCTTGGTTCTTGCAAAATGCTTTGCGCGCT |
| GTTATTCAAGTTTCTACCTTCGTGGTTTTACTTGAGTGACGCTGCTCATGCTTGCAACCG |
| CTGGGATGCAGGTGCATGCCTCTAGCATGAAGTCAGACAA |
| >GS_C31c |
| AACCAATGGCCTCCTGAACGTGCGTTGCACTCTTGGGATTTCCTGAGAGTATGTCTGCTT |
| CAGCGCTTAACTTGCCCCAACTTTGCAAGCACTTCTGCCTTGCGTTCTTATGAGCTATTG |
| CCCTCTCTGAGCCAATGGCTTGTTAATTGCTTGGTTCTTGCAAAATGCTTTGCGCGCTGT |
| TATTCAAGTTTCTACCTTCGTGGTTTTACTTGAGTGACGCTGCTCATGCTTGCAACCGCT |
| GGGATGCAGGTGCATGCCTCTAGCATGAAGTCAGACAA |
| >GS_C31.1 |
| AACCAATGGCCTCCTGAACGTGCGTTGCACTCTTGGGATTTCCTGAGAGTATGTCTGCTT |
| CAGCGCTTAACTTGCCCCAACTTTGCAAGCACTCTTCTGCCTTGCGTTCTTATGAGCTAT |
| TGCCCTCTCTGAGCCAATGGCTTGTTAATTGCTTGATTCTTGCAAAATGCTTTGCGCGCT |
| GTTATTCAAGTTTCTACCTTCGTGGTTTTACTTGAGTGACGCTGCTCATGCTTGCAACCG |
| CTGGGATGCAGGTGCATGCCTCTAGCATGAAGTCAGACAA |
| >GS_C31.5 |
| AACCAATGGCCTCCTGAACGTGCGTTGCACTCTTGGGATTTCCTGAGAGTATGTCTGCTT |
| CAGCGCTTAACTTGCCCCAACTTTGCAAGCACTCTTCTGCCTTGCGTTCTTATGAGCTAT |
| TGCCCTCTCTGAGCCAATGGCTTGTTAATTGCTTGGTTCTTGCAAAATGCTTTGCGCGCT |
| GTTATTCAAGTTTCTACTTTCGTGGTTTTACTTGAGTGACGCTGCTCATGCTTGCAACCG |
| CTGGGATGCAGGTGCATGCCTCTAGCATGAAGTCAGACAA |
| >GS_C31.6 |
| AACCAATGGCCTCCTGAACGTGCGTTGCACTCTTGGGATTTCCTGAGAGTATGTCTGCTT |
| CAGCGCTTAACTTGCCCCAACTTTGCAAGCACTCTTCTGCCTTGCGTTCTTATGAGCTAT |
| TGCCCTCTCTGAGCCAATGGCTTGTTAATTGCTTGGTTCTTGCAAAATGCTTTGCGCGCT |
| GTTATTCAAGTTTCTACCTTCGTGGTTTTACTTGAGTGACGCTGCTCATGCTTGCAACCG |
| CTGGGATGCGGGTGCATGCCTCTAGCATGAAGTCAGACAA |
| >GS_C31.9 |
| AACCAATGGCCTCCTGAACGTGGGTTGCACTCTTGGGATTTCCTGAGAGTATGTCTGCTT |
| CAGCGCTTAACTTGCCCCAACTTTGCAAGCATCTTCTGCCTTGCGTTCTTATGAGCTATT |
| GCCCTCTCTGAGCCAATGGCTTGTTAATTGCTTGGTTCTTGCAAAATGCTTTGCGCGCTG |
| TTATTCAAGTTTCTACCTTCGTGGTTTTACTTGAGTGACGCTGCTCATGCTTGCAACCGC |
| TGGGATGCAGGTGCATGCCTCTAGCATGAAGTCAGACAA |
| >GS_C31.10 |
| AACCAATGGCCTCCTGAACGTGCGTTGCACTCTTGGGATTTCCTGAGAGTATGTCTGCTT |
| CAGCGCTTAACTTGCCCCAACTTTGCAAGCATCTTCTGCCTTGCGTTCTTATGAGCTATT |
| GCCCTCTCTGAGCCAATGGCTTGTTAATTGCTTGGTTCTTGCAAAATGCTTTGCGCGCTG |
| TTATTCAAGTTTCTACCTTCGTGGTTTTACTTGAGTGACGCTGCTCATGCTTGCAACCGC |
| TGGGATGCAGGTGCATGCCTCTAGCATGAAGTCAGACAA |
| >GS_C32 |
| AACCAATGGCCTCCTGAACGTGCGTTGCACTCTTGGGATTTCCTGAGAGTATGTCTGCTT |
| CAGCGCTTAACTTGCCCCAACTTTGCAAGCATTTCTGCCTTGCGTTTTTATGAGCTATTG |
| CCCTCTGAGCCAATGGCTTGTTAATTGCTTGGTTCTTGCAAAATGCTTTGCGCGCTGTTA |
| TTCAAGTTTCTACCTTTGTGGTTTTACTTGAGTGACGCTGCTCATGCTTGCGACCGCTGG |
| GATGCAGGTGCATGCCTCTAGCATGAAGTCAGACAA |
| >GS_C33a=C33_(type_1) |
| AATCAATGGCCTCCTGAACGTGCGTTGCACTCTCGGGATTTCCTGAGAGCATGTCTGCTT |
| CAGTGCTTACCTTGCCCCAACTTTGCAAGCAGGATGTGTTTCTGCCTTGCGTTCTTATGA |
| GTTATTGCCCTCTGAGGCAATGGCTTGTTAATTGCTTGGTTCTTGCAAAATGCTTTGCGC |
| GCTGTTATTCAGGTTTCTACCTTCGTGGTTTTACTTGAGTGACGCTGCTCATGCTTGCAA |
| CCGCTGGGATGCAGGTGCATGCCTCTAGCATGAAATCAGACAA |
| >GS_C33_(type_2) |
| AATCAATGGCCTCCTGAACGTGCGTTGCACTCTCGGGATTTCCTGAGAGCATGTCTGCTT |
| CAGTGCTTACCTTGCCCCAACTTTGCAAGCAGGATGTGTTTCTGCCTTGCGTTCTTATGA |
| GTTATTGCCCTCTGAGGCAATGGCTTGTTAATGGCTTGGTTCTTGCAAAATGCTTTGCGC |
| GCTGTTATTCAGGTTTCTACCTTCGTGGTTTTACTTGAGTGACGCTGCTCATGCTTGCAA |
| CCGCTGGGATGCAGGTGCATGCCTCTAGCATGAAATCAGACAA |
| >GS_C33.1 |
| AACCAATGGCCTCCTGAACGTGCGTTGCACTCTTGGGATTTCCTGAGAGTATGTCTGCTT |
| CAGTGCTTAACTTGCCCCAACTTTGCAAGCAGGATGTGTTTCTGCCTTGCGTTCTTATGA |
| GCTATTGCCCTCTGAGCCAATGGCTTGTTAATTGCTTGGGTCTTGCAAAATGCTTTGCGC |
| GCTGTTATTCAGGTTTCTACCTTCATGGTTTTACTTGAGTGACGCTGCTCATGCTTGCAA |
| CCGCTGGGATGCAGGTGCATGCCTCTAGCATGAAGTCAGACAA |
| >GS_C34 |
| AACCAATGGCCTCCTGAACGTGCGTTGCACTCTCGGGATTTCCTGAGAGCATGTCTGCTT |
| CAGTGCTTAACTTGCCCCAACTTTGCAAGCAGGATGTGTTTCTGCCTTGCGTTCTTATGA |
| GTTATTGCCCTCTGAGGCAATGGCTTGTTAATTGCTTGGTTCTTGCAAAATACTTTGCGC |
| GCTGTTATTCAGGTTTCTACCTTCGTGGTTTTACTTGAGTGACGCTGCTCATACTTGCAA |
| CCGCTGGGATGCAGGTGCATGCCTCTAGCATGAAGTCAGACAA |
| >GS_C35_(type_1)=C35a_(type_1) |
| AACCAATGGCCTCCTGAACGTGCGTTGCACTCTTGGGATTTCCTGAGAGTATGTCTGCTT |
| CAGTGCTTACCTTGCCCCAACTTTGCAAGCAGGATGTGTTTCTGCCTTGCGTTCTTATGA |
| GCTATTGGCCTCTGAGCCAATGGCTTGTTAATTGCTTGGTTCTTGCAAAATGCTTTGCGC |
| GCTGTTATTCAAGTGTCTACCTTCGTGGTTTTACTTGAGTGACACTGCTCATGCTTGCAA |
| CCGCTGGGATGCAGGTGCATGCCTCTAGCATGAAGTCAGACAA |
| >GS_C35_(type_3) |
| AACCAATGGCCTCCTGAACGTGCGTTGCACTCTTGGGATTTCCTGAGAGTATGTCTGCTT |
| CAGTGCTTAACTTGCCCCAACTTTGCAAGCAGGATGTGTTTCTGCCTTGCGTTCTTATGA |
| GCTATTGGCCTCTGAGCCAATGGCTTGTTAATTGCTTGGTTCTTGCAAAATGCTTTGCGC |
| GCTGTTATTCAAGTGTCTACCTTCGTGGTTTTACTTGAGTGACACTGCTCATGCTTGCAA |
| CCGCTGGGATGCAGGTGCATGCCTCTAGCATGAAGTCAGACAA |
| >GS_C36 |
| AACCAATGGCCTCCTGAACGTGCGTTGCACTCTTGGGATTTCCTGAGAGTATGTCTGCTT |
| CAGTGCTTAACTTGCCCCAACTTTGCAAGCAGGATGTGTTTCTGCCTTGCGTTCTTATGA |
| GCTATTGCCCTCTGAGCCAATGGCTTGTTAATTGCTTGGTTCTTGCAAAATGCTTTGCGC |
| GCTGTTATTCAAGTTTCTACCTTCGTGGTTTTACTTGAGTGACGCTGCTCATGCTTGCAA |
| CCGCCGGGATGCAGGTGCATGCCTCTAGCATGAAGTCAGACAA |
| >GS_C37 |
| AACCAATGGCCTCCTGAACGTGCGTTGCACTCTTGGGATTTCCTGAGAGTATGTCTGCTT |
| CAGTGCTTAACTTGTCCCAACTTTGCAAGCAGGATGTGTTTCTGCCTTGCGTTCTTATGA |
| GCTATTGCCCTCTGAGCCAATGGCTTGTTAATTACTTGGTTCTTGCAAAATGCTTTGCGC |
| GCTGTTATTCAGGTTTCTACCTTCGTGGTTTTACTTGAGTGACGCTGCTCGTGCTTGCAA |
| CCGCTGGGATGCAGGTGCATGCCTCTAGCATGAAGTCAGACAA |
| >GS_C38 |
| AACCAATGGCCTCCTGAACGTGCGTTGCACTCTTGGGATTTCCTGAGAGTATGTCTGCTT |
| CAGTGCTTAACTTGCCCCAACTTTGCAAGCAGGATGTGTTTCTGCCTTGTGTTCTTATGA |
| GCTATTGCCCTCTGAGCCAATGGCTTGTTAATTGCTTGGTTCTTGCAAAATGCTTTGCGC |
| GCTGTTATTCAAGTTTCTACCTTCGTGGTTTTACTTGAGTGACGCTGCACATGCTTGCAA |
| CCGCTGGGATGCAGGTGCATGCCTCTAGCATGAAGTCAGACAA |
| >GS_C38a |
| AACCAATGGCCTCCTGAACGTGCGTTGCACTCTTGGGATTTCCTGAGAGTATGTCTGCTT |
| CAGTGCTTAACTTGCCCCAACTTTGCAAGCAGGATGTGTTTCTGCCTTGTGTTCTTATGA |
| GCTATTGCCCTCTGAGCCAATGGCTTGTTAATTGCTTGGTTCTTGCAAAATGCTTTGCGC |
| GCTGTTATTCAAGTTTCTACCCTTTGTGGTTTTACTTGAGTGACGCTGCTCATGCTTGCA |
| ACCGCTGGGATGCAGGTGCATGCCTCTAGCATGAAGTCAGACAA |
| >GS_C39 |
| AACCAATGGCCTCCTGAACGTGCGTTGCACTCTTGGGATTTCCTGAGAGTATGTCTGCTT |
| CAGTGCTTAACTTGCCCCAACTTTGCAAGCAGGATGTGTTTCTGCCTTGCGTTCTTATGA |
| GCTATTGCCCTCTGAGCCAATGGCTTGTTAATTGCTTGGTTCTTGCAAAATGCTTTGCGC |
| GCTGTTATTCAGGTTTTTACTTGAGTGACGCTGCTCATGCTTGCAACCGCTGGGATGCAG |
| GTGCATGCCTCTAGCATGAAGTCAGACAA |
| >GS_C40 |
| AACCAATGGCCTCCTGAACGTGCGTTGCACTCTTGGGATTTCCTGAGAGTATGTCTGCTT |
| CAGTGCTTAACTTGCCCCAACTTTGCAAGCAGGATGTGTTTCTGCCTTGCGTTCTTATGA |
| GCTATTGCCCTCTGAGCCAATGGCTTGTTAATTGCTTGGTTCTGGCAAAATGCTTTGCGC |
| GCTGTTATTCAAGTTTCTACCTTCGTGGTTTTACTTGAGTGACGCTGCTCATGCTTGCGA |
| CCGCTGGGATGCAGGTGCATGCCTCTAGCATGAAGTCAGACAA |
| >GS_C40a |
| AACCAATGGCCTCCTGAACGTGCGTTGCACTCTTGGGATTTCCTGAGAGTATGTCTGCTT |
| CAGTGCTTAACTTGCCCCAACTTTGCAAGCAGGATGTGTTTCTGCCTTGCATTCTTATGA |
| GCTATTGCCCTCTGAGCCAATGGCTTGTTAATTGCTTGGTTCTGGCAAAATGCTTTGCGC |
| GCTGTTATTCAAGTTTCTACCTTCGTGGTTTTACTTGAGTGACGCTGCTCATGCTTGCGA |
| CCGCTGGGATGCAGGTGCATGCCTCTAGCATGAAGTCAGACAA |
| >GS_C40b |
| AACCAATGGCCTCCTGAACGTGCGTTGCACTCTTGGGATTTCCTGAGAGTATGTCTGCTT |
| CAGTGCTTAACTTGCCCCAACTTTGCAAGCAGGATGTGTTTCTGCCTTGCGTTCTTATGA |
| GCTATTGCCCTCTGAGCCAATGGCTTGGTTAATTGCTTGGTTCTGGCAAAATGCTTTGCG |
| CGCTGTTATTCAAGTTTCTACCTTCGTGGTTTTACTTGAGTGACGCTGCTCATGCTTGCG |
| ACCGCTGGGATGCAGGTGCATGCCTCTAGCATGAAGTCAGACAA |
| >GS_C41 |
| AACCAATGGCCTCCTGAACGTGCGTTGCACTCTTGGGATTTCCTGAGAGTATGTCTGCTT |
| CAGTGCTTAACTTGCCCCAACTTTGCAAGCAGGATGTGTTTCTGCCTTGCGTTCTTATGA |
| GCTATTGCCCTCTGAGCCAATGGCTTGTTAATTGCTTGGTTCTTGCAAAATGCTTTGCGC |
| GCTGTTATTCAGGTTTCTACCTTCGTGATTTTACTTGAGTGACGCTGCTCATGCTTGCAA |
| CCGCTGGGATGCAGGTGCATGCCTCTAGCATGAAGTCAGACAA |
| >GS_C42a=C42_(type_1) |
| AATCAATGGCCTCCTGAACGTGCGTTGCACTCTTGGGATTTCCTGAGAGTATGTCTGCTT |
| CAGTGCTTAACTTGCCCCAACTTTGCAAGCAGGATGTGTTTCTGCCTTGCGTTCTTATGC |
| GCTATTGCCCTCTGAGCCAATGGCTTGTGAATTGCTTGGTTCTTGCAAAATGCTTTGCGC |
| GCTGTTATTCAGGTTTCTACCTTCGTGGTTTTACTTGAGTGACGCTGCTCATGCTTGCAA |
| CCGCTGGGATGCAGGTGCATGCCTCTAGCATGAAGTCAGACAA |
| >GS_C42_(type_2) |
| AATCAATGGCCTCCTGAACGTGCGTTGCACTCTTGGGATTTCCTGAGAGTATGTCTGCTT |
| CAGTGCTTAACTTGCCCCAACTTTGCAAGCAGGATGTGTTTCTGCCTTGCGTTCTTATGA |
| GCTATTGCCCTCTGAGCCAATGGCTTGTGAATTGCTTGGTTCTTGCAAAATGCTTTGCGC |
| GCTGTTATTCAGGTTTCTACCTTCGTGGTTTTACTTGAGTGACGCTGCTCATGCTTGCAA |
| CCGCTGGGATGCAGGTGCATGCCTCTAGCATGAAGTCAGACAA |
| >GS_C42b |
| AATCAATGGCCTCCTGAACGTGCGGTGCACTCTTGGGATTTCCTGAGAGTATGTCTGCTT |
| CAGTGCTTAACTTGCCCCAACTTTGCAAGCAGGATGTGTTTCTGCCTTGCGTTCTTATGC |
| GCTATTGCCCTCTGAGCCAATGGCTTGTGAATTGCTTGGTTCTTGCAAAATGCTTTGCGC |
| GCTGTTATTCAGGTTTCTACCTTCGTGGTTTTACTTGAGTGACGCTGCTCATGCTTGCAA |
| CCGCTGGGATGCAGGTGCATGCCTCTAGCATGAAGTCAGACAA |
| >GS_C43 |
| AATCAATGGCCTCCTGAACGTGCGTTGCACTCTTGGGATTTCCTGAGAGTATGTCTGCTT |
| CAGTGCTTAACTTGCCCCAACTTTGCAAGCAGGATGTGTTTCTGCCTTGCGTTCTTATGA |
| GTTATTGTCCTCTGAGCCAATGGCTTGTGAATTGCTTGGTTCTTGCAAAATGCTTTGCGC |
| GCTGTTATTCAGGTTTCTACCTTCGTGGTTTTACTTGAGTGACGCTGCTCATGCTTGCAA |
| CCGCTGGGATGCAGGTGCATGCCTCTAGCATGAAGTCAGACAA |
| >GS_C44 |
| AACCAATGGCCTCCTGAACGTGCGTTGCACTCTTGGGATTTCCTGAGAGTATGTCTGCTT |
| CAGTGCTTAACTTGCCCCAACTTTGCAAGCAGGATGTGTTTCTGCCTTGCGTTCTTATGA |
| GCTATTGCCCTCTGAGCCAATGGCTTGTGAATTGCTTGGTTCTTGCAAAATGCTTTGTGC |
| GCTGTTATTCAGGTTTCTACCTTCGTGGTTTTACTTGAGTGACGCTGCTCATGCTTGCAA |
| CCGCTGGGATGCAGGTGCATGCCTCTAGCATGAAGTCAGACAA |
| >GS_C44a |
| AACCAATGGCCTCCTGAACGTGCGTTGCACTCTTGGGATTTCCTGAGAGTATGTCTGCTT |
| CAGTGCTTAACTTGCCCCAACTTTGCAAGCAGGATGTGTTTCTGCCTTGCGTTCTTATGA |
| GCTATTGCCCTCTGAGCCAATGGCTTGTGAATTGCTTGGTTCTTGCAAAATGCTTTGTGC |
| GCTGTTATTCAGGTTTCTACCTTCGTGGTTTTACTTGAGTGATGCTGCTCATGCTTGCAA |
| CCGCTGGGATGCAGGTGCATGCCTCTAGCATGAAGTCAGACAA |
| >GS_C45.3 |
| AACCAATGGCCTCCTGAACGTGCGTTGCACTCTTGGGATTTCCTGAGAGTATGTCTGCTT |
| CAGTGCTTAACTTGCCCCAACTTTGCAAGCAGGATGTGCTTCTGCCTTGCGTTCTTATGA |
| GCTATTGCCCTCTGAGCCAATGGCTTGTGAATTGCTTGGTTCTTGCAAAATGCTTTGCGC |
| GCTGTTATTCAGGTTTCTACCTTCGTGGTTTTACTTGAGTGACGCTGCTCATGCTTGCAA |
| CCGCTGGGATGCAGGTGCATGCCTCTAGCATGAAGTCAGACAA |
| >GS_C45a |
| AACCAATGGCCTCCTGAACGTGCGTTGCACTCTTGGGATTTCCTGAGAGTATGTCTGCTT |
| CAGTGCTTAACTTGCTCCAACTTTGCAAGCAGGATGTGTTTCTGCCTTGCGTTCTTATGA |
| GCTATTGCCCTCTGAGCCAATGGCTTGTGAATTGCTTGGTTCTTGCAAAATACTTTGTGC |
| GCTGTTATTCAGGTTTCTACCTTCGTGGTTTTACTTGAGTGACGCTGCTCATGCTTGCAA |
| CCGCTGGGATGCAGGTGCATGCCTCTAGCATGAAGTCAGACAA |
| >GS_C46 |
| AACCAATGGCCTCCTGAACGTGCGTTGCACTCTTGGGATTTCCTGAGAGTATGTCTGCTT |
| CAGTGCTTAACTTGCCCCAACTTTGCAAGCAGGATGTGTTTCTGCCTTGCGTTCTTATGA |
| GCTATTGCCCTCTGAGCCAATGGCTTGTGAATTGCTTGGTTCTTGCAAAATGCTATGCGC |
| GCTGTTATTCAGGTTTCTACCTTCGTGGTTTTACTTGAGTGACGCTGCTCATGCTTGCAA |
| CCGCTGGGATGCAGGTGCATGCCTCTAGCATGAAGTCAGACAA |
| >GS_C46a |
| AACCAATGGCCTCCTGAACGTGCGTTGCACTCTTGGGATTTCCTGAGAGTATGTCTGCTT |
| CAGTGCTTAACTTGCCCCAACTTTGCAAGCAGGACGTGTTTCTGCCTTGCGTTCTTATGA |
| GCTATTGCCCTCTGAGCCAATGGCTTGTGAATTGCTTGGTTCTTGCAAAATGCTATGCGC |
| GCTGTTATTCAGGTTTCTACCTTCGTGGTTTTACTTGAGTGACGCTGCTCATGCTTGCAA |
| CCGCTGGGATGCAGGTGCATGCCTCTAGCATGAAGTCAGACAA |
| >GS_C47 |
| AACCAATGGCCTCCTGAACGTGCGTTGCACTCTTGGGATTTCCTGAGAGTATGTCTGCTT |
| CAGTGCTTAACTTGCCCCAACTTTGCAAGCAGGATGTGTTTCTGCCTTGCGTTCTTATGA |
| GCTATTGCCCTCTGCGCCAATGGCTTGTGAATTGCTTGGTTCTTGCAAAATGCTTTGTGC |
| GCTGTTATTCAGGTTTCTACCTTCGTGGTTTTACTTGAGTGACGCTGCTCATGCTTGCAA |
| CCGCTGGGATGCAGGTGCATGCCTCTAGCATGAAGTCAGACAA |
| >GS_C48 |
| AACCAATGGCCTCCTGAACGTGCGTTGCACTCTTGGGATTTCCTGAGAGTATGTCTGCTT |
| CAGTGCTTAACTTGCCCCAACTTTGCAAGCAGGATGTGTTTCTGCCTTGCGTTCTTATGA |
| GCTATTGCCCTCTGAGCCAATGGCTTGTTAATTGCTTGGTTCTTGCAAAATGCTTTGCGC |
| GTGCTGTTATTCAAGTTTTTACCTTCGTGGTTTTACTTGAGTGACGCTGCTCATGCTTGC |
| AACCGCTGGGATGCAGGTGCATGCCTCTAGCATGAAGTCAGACAA |
| >GS_C49 |
| AACCAATGGCCTCCTGAACGTGCGTTGCACTCTTGGGATTTCCTGAGAGTATGTCTGCTT |
| CAGTGCTTAACTTGCCCCAACTTTGCAAGCAGGATGTGTTTCTGCCTTGCGTTCTTATGA |
| GCTATTGCCCTCTGAGCCAATGGCTTGTTAATTGCTTGGTTCTTGCAAAATGCTGAGCCA |
| ATGGCTTGTTAATTGCTTGGTTCTTGCAAAATGCTTTGCGCGCTGTTATTCAAGTTTCTA |
| CCTTCGTGGTTTTACTTGAGTGACGCTGCTCATGCTTGCAACCGCTGGGATGCAGGTGCA |
| TGCCTCTAGCATGAAGTCAGACAA |
| >GS_C50 |
| AACCAATGGCCTCCTGAATGTGCGTTGCACTCTTGGGATTTCCTGAGAGTATGTCTGCTT |
| CAGTGCTTAACTTGCCCCAACTTTGCAAGCAGGATGTGTTTCTGCCTTGCGTTCTTATGA |
| GCTATTGCCCTCTGAGCCAATGGCTTGTTAATTGCTTGGTTCTTGCAAAATGCTTTGCGC |
| GCTGTTATTCAAGTTTCTACCTTCGTGGTTTTACTTGAGTGACACTGCTCATGCTTGCAA |
| CCGCTGGGATGCAGGTGCATGCCTCTAGCATGAAGTCAGACAA |
| >GS_C51 |
| AAGCAATGGCCTCCTGAACGTGTGTTGCACTCTTGGGATTTCCTGAGAGTATGTCTGCTT |
| CAGTGCTTAACTTGCCCCAACTTTGCAAACAGGATGTGTTTCTGCCTTGCGTTCTTATGA |
| GCTATTGCCCTCTGAGCCAATGGCTTGTTAATTGCTTGGTTCTTGCAAAATGCTTTGCGC |
| AATGTTATTCAAGTTTCTACCTTCGTGGTTTTACTTGAGTGACGCTGCTCATGCTTGCAA |
| CCGCTGGGATGCAGGTGCATGCCTCTAGCATGAAGTCAGACAA |
| >GS_C52 |
| AGCCAATGGCCTCCTGAACGTGCGTTGCACTCTTGGGATTTCCTGAGAGTATGTCTGCTT |
| CAGTGCTTAACTTGCCCCAACTTTGCAAGCAGGATGTGTTTCTGCCTTGCGTTCTTACGA |
| GCTATTGCCCTCTGAGCCAATGGCTTGTTAATTGCTTGGTTCTTGCAAAATGCTTTGCGC |
| GCTGTTATTCAAGTTTCTACCTTCGTGGTTTACTTGAGTGACGCTACTCATGCTTGCAAC |
| CGCTGGGATGCAGGTGCATGCCTCTAGCATGAAGTCAGACAA |
| >GS_C53 |
| AACCAATGGCCTCCTGAACGTGCGTTGCACTCTTAGTATTTCCTGAGAGTATGTCTGCTT |
| CAGTGCTTACCTTGCCCCAACTTTGCAAGCAGGATGTGTTTCTGCCTTGCGTTCTTATGA |
| GCTATTGCCCTCTGAGCCAATGGCTTGTTAATTGCTTGGTTGTTGCAAAATGCTTTGCGC |
| GCTGTTACTCAAGTTTCTACCTTCGTGGGTTTACTGGAGTGACGCTGCTCATGCTTGCAA |
| CCGCTGGGATGCAGGTGCATGCCTCTAGCATGAAGTCAGACAA |
| >GS_C54a |
| AATCAATGGCCTCCTGAACGTGCGTTGCACTCTTGGGATTTCCTGAGAGTATGTCTGCTT |
| CAGTGCTTAACTTGCCCCAACTTTGCAAGCAGGATGTGTTTCTGCCTTGCGTTCTTATGA |
| GCTATTGCCCTCTGAGCCAATGGCTTGTTACTTGCTTGGTTCTTGCAAAATGCTTTGCGC |
| GCTGTTATTCAAGTTTCTACCTTCGTGGTTTTACTTGAGTGACACTGCTCATGCTTGCAA |
| CCGCTGGGATGCAGGTGCATGCCTCTAGCATGAAGTCAGACAA |
| >GS_C15=C55 |
| AACCAATGGCCTCCTGAACGTGCGTTGCACCCTTGGGATTTCCTGAGAGTATGTCTGCTT |
| CAGTGCTTAACTTGCCCCAACTTTGCAAGCAGGATGTGTTTCTGCCTTGCGTTCTTATGA |
| GCTATTGCCTTCTGCGCCAATGGCTTGTTAATTGCTTGGTTCTTGCAAAATGCTTTGCGC |
| GCTGTTATTCAAGTTTCTTTCTACCTTCGCGGTTTTACTTGAGTGACGCTGCTCATGCTT |
| GCAACCGCTGGGATGCAGGTGCATGCCTCTAGCATGAAGTCAGACAA |
| >GS_C56 |
| AACCAATGGCCTCCTGAACGTACGTTGCACCCTTGGGATTTCCTGAGAGTATGTCTGCTT |
| CAGTGCTTAACTTGCCCCAACTTTGCAAGCAGGATGTGTTTCTGCCTTGCGTTCTTATGA |
| GCTATTGCCTTCTGCGCCAATGGCTTGTTAATTGCTTGGTTCTTGCAAAATGCTTTGCGC |
| GCTGCTATTCACGTTTCTACCTTCGCGGTTTTACTTGAGTGACGCTGCTCATGCTTGCAA |
| CCGCTGGGATGCAGGTGCATGCCTCTAGCATGAAGTCAGACAA |
| >GS_C56a |
| AACCAATGGCCTCCTGAACGTGCGTTGCACCCTTGGGATTTCCTGAGAGTATGTCTGCTT |
| CAGTGCTTAACTTGCCCCAACTTTGCAAGCAGGATGTGTTTCTGCCTTGCGTTCTTATGA |
| GCTATTGCCTTCTGCGCCAATGGCTTGTTAATTGCTTGGTTCTTGCAAAATGCTTTGCGC |
| GCTGATATTCAAGTTTCTACCTTCGCGGTTCTACTTGAGTGACGCTGCTCATGCTTGCAA |
| CCGCTGGGATGCAGGTGCATGCCTCTAGCATGAAGTCAGACAA |
| >GS_C57a |
| AACCAATGGCCTCCTGAACGTGCGTTGCACTCTTGGGATTTCCTGAGAGTATGTCTGCTT |
| CAGTGCTTAACTTGCCCCAACTTTGCAAGCAGGATGTTTTTCTGCCTTGCGTTCTTATGA |
| GCTGTTGTCCTCTGTGCCAATGGCTTGTTAATTGCTTGGTGCTTGCAAAATGCTTTGCGC |
| GCTGTTATTCAAGTTTCTACCTTCGTGGTTTTACTTGAGTGACGCTGCTCATGCTTGCAA |
| CCCGCTGGGATGCAGGTGCATGCCTCTAGCATGAAGTCAGACAA |
| >GS_C57 |
| AACCAATGGCCTCCTGAACGTGCGTTGCACTCTTGGGATTTCCTGAGAGTATGTCTGCTT |
| CAGTGCTTAACTTGCCCCAACTTTGCAAGCAGGATGTTTTTCTGCCTTGCGTTCTTATGA |
| GCTATTGTCCTCTGTGCCAATGGCTTGTTAATTGCTTGGTGCTTGCAAAATGCTTTGCGC |
| GCTGTTATTCAAGTTTCTACCTTCGTGGTTTTACTTGAGTGACGCTGCTCATGCTTGCAA |
| CCCGCTGGGATGCAGGTGCATGCCTCTAGCATGAAGTCAGACAA |
| >GS_C58 |
| AACCAATGGCCTCCTGAACGTGCGTTGCACTCTTGGGATTTCCTGAGAGTATGTCTGCTT |
| CAGTGCTTAACTTGCCCCAACTTTGCAAGCATTTCTGCCTTGCGTTCTTATGAGCTATTG |
| CCCTCTGAGCCCAATGGCTTGTTAATTGCTTGGTTCTTGCAAAATGCTTTGCGCGCTGTT |
| ATTCAAGTTTCTACCTTCGTGGTTTTACTTGAGTGACGCTGCTCATGCTTGCAACCGCTG |
| GGATGCAGGTGCATGCCTCTAGCATGAAGTCAGACAA |
| >GS_C59 |
| AACCAATGGCCTCCTGAACGTGCGTTGCACTCTTGGGATTTCCTGAGAGTATGTCTGCTT |
| CAGTGCTTAACTTGCCCCAACTTTGCAAGCAGGATGTGTTTCTGCCTTTGCGCTCTTATG |
| AGCTATTGCCCTCTGAGCCAATGGCTTGTTAATTGCTTGGTTCTTGCAAACTGCTTTGCG |
| CGTTGTTATTCAGGTTTCTACCTTCGTGGTTTTACTTGAGTGACGGTGCTCATTGCTTGC |
| AACCGCTGGGATGCAGGTGCATGCCTCTAGCATGAAGTCAGACAA |
| >GS_C60 |
| AACCAATGGCCTCCTGAACGTGCGTTGCACCCTTGGGATTTCCTGAGAGTATGTCTGCTT |
| CAGTGCTTAACTTGCCCCAACTTTGCAAGCAGGATGTGTTTCTGCCTTGCGTTCTTATGA |
| GCTACTGCCTTCTGCGCCAATGGCTTGTTAATTGCTTGGTTCTTGCAAAATGCTTTGCGC |
| GCTGTTATTCAAGTTTCTACCTTCGCGGTTTTACTTGAGTGACGCTGCTCATGCTTGCAA |
| CCGCTGGGATGCAGGTGCATGCCTCTAGCATGAAGTCAGACAA |
| >GS_C61 |
| AACCAATGGCCTCCTGAACGTGCGTTGCACTCTTGGGATTTCCTGAGTGTATGTCTGCTT |
| CAATGCTTAACTTGCCCCAACTTTGCAAGCATTTTTCTGCCTTGCGTTTTTATGAGCTAT |
| TGCCCTCTGAGCCAATGGCTTGTTAATTGCTTGGTTCTTGCAAAATGCTTTGTGCGCTGT |
| TATTCAAGTTTCTACCGTCGTGGTTTTACTTGAGTGATGCTGCTCATGCTTGCAACCGCT |
| GGATGCAGGTGCATGCCTCTAGCATGAAGTCAGACAA |
| >GS_C62 |
| AACCAATCGCCTCCTGAACGTGCGTTGCACTCTTGGGATTTCCTGAGAGTATGTCTGCTT |
| CAGTGCTTAACTTGCCCCAACTTTGCAAGCAGGATGTGTTTCTGCCTTGCGTTCTTACGA |
| GCTATTGCCCTCTGAGCCAATGGCTTGTTAATTGCTTGGTTCTTGCAAAATGCTTTGCGC |
| GCTGTTATTCAAGTTTCTACCTTCGTGGTTTTACTTGAGTGACGCTGCTCATGCTTGCAA |
| CCGCTGGGATGCAGGTGCATGCCTCTAGCATGAAGTCAGACAA |
| >GS_C63a |
| AACCAATGGCCTCCTGAACGTGCGTTGCACTCTTGGGATTTCCTGAGAGTATGTCTGCTT |
| CAGTGCTTAACTTGCCCCAACTTTGCAAGCATTTCTGCCTTGCGTTCTTATGAGCTATTG |
| CCCTCTCTGAGCCAATGGCTTGTTAATTGCTTGGTTCTTGCGAAATGCTTTGCGCGCTGT |
| TATTCAAGGTTCTACCTTCGTGGTTTTACTTGAGTGACGCTGCTCATGCTTGCAACCGCT |
| GGGATGCAGGTGCATGCCTCTAGCATGAAGTCAGACAA |
| >GS_C64 |
| AACCAATGGCCTCCTGAACGTGCGTTGCACTCTTGGGATTTCCTGAGAGTATGTCTGCTT |
| CAGTGCTTAACTTGCCCCAACTTTGCAAGCAGGATGTGTTTCTGCCTTGTGTTCTTATGA |
| GTATTGCCCTCTGAGCCAATGGCTTGTTAATTGCTTGGTTCTTGCAAAATGCTTTGTGCG |
| CTGTTATTCAAGTTTCTACTTTCGTGGTTTTACTTGAGTGACGCTGCTCATGCTTGCAAC |
| CGCTGGGATGCAGGTGCATGCCTCTAGCATGAAGTCAGACAA |
| >GS_C65 |
| AACCAATGGCCTCCTGAACGTGCGTTGCACTCTTGGGATTTCCTGAGAGTATGTCTGCTT |
| CAGTGCTTAACTTGCCCCAACTTTGCAAGCAGGATGTGTTTCTGCCTTGCGTTCTTATGA |
| GCTATTGCCCTCTGAGCCAATGGCTTGTTAATTGCTTGGTTCTTGCAAAATGCTTTGCGC |
| GCTGTTATTCAAGTTTCTACCTTCGTCGTTTTACTTGAGTGACGCTGCTCATGCTTGCAA |
| CCGCTGGGATGCAGGTGCATGCCTCTAGCATGAAGTCAGACAA |
| >GS_C65a |
| AATCAATGGCCTCCTGAACGTGCGTTGCACTCTTGGGATTTCCTGAGAGTATGTCTGCTT |
| CAGTGCTTAACTTGCCCCAACTTTGCAAGCAGGATGTGTTTCTGCCTTGCGTTCTTATGA |
| GCTATTGCCCTCTGAGCCAATGGCTTGTTAATTGCTTGGTTCTTGCAAAATGCTTTGCGC |
| GCTGTTATTCAAGTTTCTACCTTCGTCGTTTTACTTGAGTGACGCTGCTCATGCTTGCAA |
| CCGCTGGGATGCAGGTGCATGCCTCTAGCATGAAGTCAGACAA |
| >GS_C66 |
| AACCAATGGCCTCCTGAACGTGCGTTGCACTCTTGGGATTTCCTGAGAGTATGTCTGCTT |
| CAGTGCTTAACTTGCCCCAACTTTGCAAGCAGGATGTTTTTCTGCCTTGCGTTCTTATGA |
| GCTATTGTCCTCTGCGCCAATGGCTTGTTAATTGCTTGGTGCTTGCAAAATGCTTTGCGC |
| GCTGTTATTCAAGTTTCTACCTTCGTGGTTTTACTTGAGTGACGCTGCTCATGCTTGCAA |
| CCCGCTGGGATGCAGGTGCATGCCTCTAGCATGAAGTCAGACAA |
| >GS_C66a |
| AACCAATGGCCTCCTGAACGTCTGCTTCAGTGCTTAACTTGCCCCAACTTTGCAAGCAGG |
| ATGTTTTTCTGCCTTGCGTTCTTATGAGCTATTGTCCTCTGCGCCAATGGCTTGTTAATT |
| GCTTGGTGCTTGCAAAATGCTTTGCGCGCTGTTATTCAAGTTTCTACCTTCGTGGTTTTA |
| CTTGAGTGACGCTGCTCATGCTTGCAACCCGCTGGGATGCAGGTGCATGCCTCTAGCATG |
| AAGTCAGACAA |
| >GS_C66b |
| AACCAATGGCCTCCTGAACGTGCGTTGCACTCTTGGGATTTCCTGAGAGTATGTCTGCTT |
| CAGTGCTTAGCTTGCCCCAACTTTGCAAGCAGGATGTTTTTCTGCCTTGCGTTCTTATGA |
| GCTATTGTCCTCTGCGCCAATGGCTTGTTAATTGCTTGGTGCTTGCAAAATGCTTTGCGC |
| GCTGTTATTCAAGTTTCTACCTTCGTGGTTTTACTTGAGTGACGCTGCTCATGCTTGCAA |
| CCCGCTGGGATGCAGGTGCATGCCTCTAGCATGAAGTCAGACAA |
| >GS_C67 |
| AACCAATGGCCTCCTGAACGTGCGTTGCACTCTTGGGATTTCCTGAGAGTATGTCTGCTT |
| CAGTGCTTAACTTGCCCCAACTTTGCAAGCAGGATGTGTTTCTGCCTTGCGTTCTTATGA |
| GCTATTGCCCTCCGAGCCAATGGCTTGTGAATTGCTTGGTTCTTGCAAAATGCTTTGCGC |
| GCTGTTATTCAGGTTTCTACCTTCGTGGTTTTACTTGAGTGACGCTGCTCATGCTTGCAA |
| CCGCTGGGATGCAGGTGCATGCCTCTAGCATGAAGTCAGACAA |
| >GS_C69 |
| AACCAATGGCCTCCTGAACGTGCGTTGCACTCTTGGGATTTCCTGAGAGAATGTCTGCTT |
| CAGTGCTTAACTTGCCCCAACTTTGCAAGCAGGATGTGTTTCTGCCTTGCGTTCTTATGA |
| GCTATTGCCCTCTGAGCCAATGGCTTGTTAATTCCTTGGTTCTTGCAAAATGCTTTGCGC |
| GCTGTTACTCAGATTTCTACCTGAGTGACGCTGCTCATGCTTGCAAACCGCTGGGATGCA |
| GGTGCATGCCTCTAGCATGAAGTCAGACAA |
| >GS_C69a |
| AATCAATGGCCTCCTGAACGTGCGTTGCACTCTTGGGATTTCCTGAGAGAATGTCTGCTT |
| CAGTGCTTAACTTGCCCCAACTTTGCAAGCAGGATGTGTTTCTGCCTTGCGTTCTTATGA |
| GCTATTGCCCTCTGAGCCAATGGCTTGTTAATTCCTTGGTTCTTGCAAAATGCTTTGCGC |
| GCTGTTACTCAGATTTCTACCTGAGTGACGCTGCTCATGCTTGCAAACCGCTGGGATGCA |
| GGTGCATGCCTCTAGCATGAAGTCAGACAA |
| >GS_C70 |
| AACCAATGGCCTCCTGAACGTGCGTTGCACTCTTGGGATTTCCTGAGAGTATGTCTGCTT |
| CAGTGCTTAACTTGCCCCAACTTTGCAAGCAGGATGTGTTTCTGCCTTGCGTTCTTACGA |
| GCTATTGCCCTCTGAGCCAATGGCTTGTTAATTGCTTGGTTCTTGCAAAATGCTTTGCGC |
| GCTGTTATTCAAGTTTCTACCTTCGTGGTTTTACTTGAGTGACGCTGCTCATGCTTGCAA |
| CCGCTGGGATGCAGGTGCATGCCTCTAGCATGAAGTCAGACAA |
| >GS_C71 |
| AACCAATGGCCTCCTGAACGTGCGTTGCACTCTTGGGATTTCCTGAGAGTATGTCTGCTT |
| CAGTGCTTAACTTGCCCCAACTTTGCAAGCAGGATGTGTTTCTGCCTTGCGTTCTTATGA |
| GCTATTGCCCTCTGAGCCAATGGCTTGTTAATTGCTTGGTTCTTGCAAAATGCTTTGCGC |
| GCTGTTATTCAAGTTTCTACCTTCGTCGTTTTACTTGAGTGACGCTGCTCGTGCTTGCAA |
| CCGCTGGGATGCAGGTGCATGCCTCTAGCATGAAGTCAGACAA |
| >GS_C71a |
| AGCCAATGGCCTCCTGAACGTGCGTTGCACTCTTGGGATTTCCTGAGAGTATGTCTGCTT |
| CAGTGCTTAACTTGCCCCAACTTTGCAAGCAGGATGTGTTTCTGCCTTGCGTTCTTATGA |
| GCTATTGCCCTCTGAGCCAATGGCTTGTTAATTGCTTGGTTCTTGCAAAATGCTTTGCGC |
| GCTGTTATTCAAGTTTCTACCTTCGTCGTTTTACTTGAGTGACGCTGCTCGTGCTTGCAA |
| CCGCTGGGATGCAGGTGCATGCCTCTAGCATGAAGTCAGACAA |
| >GS_C72 |
| AAGCAATGGCCTCCTGAACGTGCGTTGCACTCTTGGGATTTCCTGAGAGTATGTCTGCTT |
| CAGTGCTTAACTTGCCCCAACTTTGCAAGCAGGATGTGTTTCTGCCTTGCGTTCTTATGA |
| GCTATTGCCCTCCAATGGCTTGTTAATTGCTTGGTTCTTGCAAAATGCTTTGCGCGCTGT |
| TATTCAGGTTTCTACCTTCGTGGTTTTACTTGAGTGACGCTGCTCATGCTTGCAACCGCT |
| GGGATGCAGGTGCATGCCTCTAGCATGAAGTCAGACAA |
| >GS_C73 |
| AACCAGTGGCCTCCTGAACGTGCGTTGCACTCTTGGGATTTCCTGAGAGTATGTCTGCTT |
| CAGCGCTTAACTTGCCCCAACTTTGCAAGCATTTCTGCCTTGCGTTCTTATGAGCTATTG |
| CCCTCTGAGCCAATGGCTTGTTAATTGCTTGGTTCTTGCAAAATGCCTTGCGCGCACTGT |
| TATTCAAGCTTCTACCTTCGTGGTTTTACTTGAGTGATGACGCTGCTCATGCTTGCAACC |
| GCTGGGATGCAGGTGCATGCCTCTAGCATGAAGTCAGACAA |
| >GS_C74 |
| AACCAATGGCCTCCTGAACGTGCGTTGCACTCTTGGGATTTCCTGAGAGTATGTCTGCTT |
| TAGTGCTTAACTTGCCCCAACTTTGCAAGCAGGATGTTTTTCTGCCTTGCGTTCTTATGA |
| GCTATTGTCCTCTGCGCCAATGGCTTGTTAATTGCTTGGTGCTTGCAAAATGCTTTGCGC |
| GCTGTTATTCAAGTTTCTACCTTCGTGGTTTTACTTGAGTGATGCTGCTCATGCTTGCAA |
| CCCGCTGGGATGCAGGTGCATGCCTCTAGCATGAAGTCAGACAA |
| >GS_C75 |
| AACCAATGGCCTCCTGAACGTGCGTTGCACTCTTGGGATTTCCTGAGAGTATGTCTGCTT |
| CAGTGCTTAACTTGCCCCAACTTTGCAAGCAGGATGTGTTTCTGCCTTGCGGTTTTATGA |
| GCTATTGCCCTCTGAGCCAATGGCTTGTTAATTGCTTGGTTCTTGCAAAATGCTTTGCGC |
| GCTGTTATTCAAGTTTCTACCTTCGTGGTTTTACTTGAGTGACGCTGCTCATGCTTGCAA |
| CCGCTGGGATGCAGGTGCATGCCTCTAGCATGAAGTCAGACAA |
| >GS_C76 |
| AATCAATGGCCTCCTGAACGTGCATTGCACTCTTGGGATTTCCTGAGAGTATGTCTGCTT |
| CAGTGCTTAACTTGCCCCAACTTTGCAAGCAGGATGTGTTTCTGCCTTGCGTTCTTATGA |
| GCTATTGCCCTCTGAGCTAATGGCTTGTTAATTGCTTGGTCTTGCAAAATGCTTTGCGCG |
| CTTTATTCAAGTTTCTACCTTCGTGGTTTTACTTGAGTGACGCTGCTCATGCTTGCAACC |
| GCTGGGATGCAGGTGCATGCCTCTAGCATGAAGTCAGACAA |
| >GS_C77 |
| AAGCAATGGCCTCCTGAACGTGCGTTGCACTCTTGGGATTTCCTGAGAGTATGTCTGCTT |
| CAGTGCTTAACTTGCCCCAACTTTGCAAGCAGGATGTGTTTCTGCCTTGCGTTCTTATGA |
| GCTATTGCCCTCTGAGCTAATGGCTTGTTAATTGCTTGGTCTTGCAAAATGCTTTGCGCG |
| CGCTGTCATTCAAGTTTCTACCTTCGTGGTTTTACTTGAGTGACGCTGCTCATGCTTGCA |
| ACCGCTGGGATGCAGGTGCATGCCTCTAGCATGAAGTCAGACAA |
| >GS_C78 |
| AATCAATGGCCTCCTGAACGCGCGTTGCAGTCTTGGGATTTCCTGAGAGTATGTCTGCTT |
| CAGTGCTTAACTTGCCCCAACTTTGCAAGCAGGATGTGTTTCTGTCTTGCGTTCGTATGA |
| GCTATTGCCCTCTGAGCCAGTGGCTTGTTAATTGCTTGGTTCTTGCAACATGCTTTGCGC |
| GCTGTTATTCAGGTTTCTACCTTCGCGGTTTTACTTGAGTGACACTGCTCATGCTTGCAA |
| CCGCTGGGATGGAGGTGCATGCCTCTAGCATGAAGTCAGACAA |
| >GS_C78a |
| AACCAATGGCCTCCTGAACGTGCGTTGCACTCTTGGGATTTCCTGAGAGTATGTCTGCTT |
| CAGTGCTTAACTTGCCCCAACTTTGCAAGCAGGATGTGTTTCTGCCTTGCGTTCTTATGA |
| GCTATTGCCCTCTGAGCCAATGGCTTGTTAATTGCTTGGTTCTTGCAAAATGCTTTGCGC |
| GCTGTTATTCAGGTTTCTACCTTCGTGGTTTTACTTGAGTGACGCTGCTCATGCTTGCNA |
| CCGCTGGGATGCAGGTGCATGCCTCTAGCATGAAGTCAGACAA |
| >GS_C79 |
| AACCAATGGCCTCCTGAACGTGCGTGGCACTCTTGGGATTTCCTGAGAGTATGTCTGCTT |
| CAGTGCTTAACTTGCCCCAACTTTGCAAGCAGGATGTGTTTCTGCCTTGCGTTCTTATGA |
| GCTATTGGCCTCTGAGCCAATGGCTTGTTAATTACTTGGTTCTTGCAAAATGCTTTGCGC |
| GCTGTTATTCAAGTTTCTACCTTCGTGGTTTTACTTGAGTGACACTGCTCATGCTTGCGA |
| CCGCTGGGATGCAGGTGCATGCCTCTAGCATGAAGTCGGACAA |
| >GS_C1a=C80 |
| AACCAATGGCCTCCTGAACGTGCGTTGCACTCTTGGGATTTCCTGAGAGTATGTCTGCTT |
| CAGTGCTTAACTTGCCCCAACTTTGCAAGCAGGATGTGTTTCTGCCTTGCGTTCTTATGA |
| GCTATTGCCCTCTGAGCCAATGGCTTGTTAATTGCTTGGTTCTTGCAAAATGCTTTGCGC |
| GCTGTTATTCAGGTTTCTACCTTCGCGGTTTTACTTGAGTGACGCTGCTCATGCTTGCAA |
| CCGCTTGCAACCGCTGGGATGCAGGTGCATGCCTCTAGCATGAAGTCAGACAA |
| >GS_C81 |
| AACCAATGGCCTCCTGAACGTGCGTTGCACTCTTGGGATTTCTTTCCTGAGAGTATGTCT |
| GCTTCAGTGCTTAACTTGCCCCAAATTTGCAAGCAGGATGTGTTTCTGCCTTGTGTTCTT |
| ATGAGCTATTGCCCTCTGAGCCAATGGCTTGTTAATTGCTTGGTTCTTGCAAAATGCTTT |
| GCGCGCTGTTATTCAAGTTTCTACCGCTGCTCATGCTTGCAACCGCTGGGATGCAGGTGC |
| ATGCTTCTAGCATGAAGTCAGACAA |
| >GS_C82=C82b |
| AACCAATGGCCTCCTGAACGTGCGTTGCACTCTTGGGATTTCCTGAGAGTATGTCTGCTT |
| CAGTGCTTAACTTGCCTCAATTTTGCAAGCAGGATGTGTTTCTGCCTTGCGTTCTTATGA |
| GCCATTGCCCTCTGAGCCAATGGCTTGTGAATTGCTTGGTTCTTGCAAAATGCTTTGCGC |
| GCTGTTATTCAGGTTTCTACCTTCGTGGTTTTACTTGAGTGACGCTGCTCATGCTTGCAA |
| CCGCTGGGATGCAGGTGCATGCCTCTAGCATGAAGTCAGACAA |
| >GS_C3=C83=Cspc |
| AACCAATGGCCTCCTGAACGTGCGTTGCACTCTTGGGATTTCCTGAGAGTATGTCTGCTT |
| CAGTGCTTAACTTGCCCCAACTTTGCAAGCAGGATGTGTTTCTGCCTTGCGTTCTTATGA |
| GCTATTGCCCTCTGAGCCAATGGCTTGTTAATTGCTTGGTTCTTGCAAAATGCTTTGCGC |
| GCGCTGTTATTCAAGTTTCTACCTTCGTGGTTTTACTTGAGTGACGCTGCTCATGCTTGC |
| AACCGCTGGGATGCAGGTGCATGCCTCTAGCATGAAGTCAGACAA |
| >LJ_C84 |
| TTGTCTGACTTCATGCTAGAGGCATGCACCTGCATCCCAGCGGTTGCAAGCATGAGCAGCGTCACTCAACTAAAACCACGAAGGTAGAAACTTGAATAACAGCGCGCAGCATTTTGCAAGAACCAAGCAATTAACAAGCCATTGCCTCAGAGGGCAATAGTTCATAAGAAAACGCAAGGCAGAAACACATCCTGCTTGCAAAGTTGGGGCAAGTTAAGCACTGAAGCAGACATACTCTCAGGAAATCCCAAGAGTGCAACGCACATTCAGGAGGCCACTGGTT |
| >LJ_C84a |
| TTGTCTGACTTCATGCTAGAGGCATGCACCTGCATCCCAGCGGTTGCAAGCATGAGCAGCGTCACTCAACTAAAACCACGAAGGTAGAAACTTGAATAACAGCGCGCAGCATTTTGCAAGAACCAAGCAATTAACAAGCCATTGCCTCAGAGGGCAATAGTTCATAAGAAAATGCAAGGCAGAAACACATCCTGCTTGCAAAGTTGGGGCAAGTTAAGCACTGAAGCAGACATACTCTCAGGAAATCCCAAGAGTGCAACGCACATTCAGGAGGCCACTGGTT |
| >LJ_C86 |
| TTGTCTGACTTCATGCTAGAGGCATGCACCTGCATCCCAGCGGTTGCAAGCATGAGCAGCGTCACTCAAGTAAAACCACGAAGGTAGAAACCTGAATAACAGCGGTCAAAGCATTTTGCAAGAACCAAGCAATTCACAAGCCATTGGCTCAGAGGGCAATAGCTCATAAGAACGCAAGGCAGAAACACATCCTGCTTGCAAAGTTGGGGCAAGTTAAGCACTGAAGCAGACATACTCTCAGGAAATCCCAAGAGTGCAACGCACGTTCAGGAGGCCATTGGTT |
| >LJ_C87 |
| TTGTCTGACTTCATGCTAGAGGCATGCACCTGCATCCCAGCGGTTGCAAGCATGAGCAGCGTCACTCAAGTAAAACCACGAAGGTAGAAACTTGAATAACAGCGCGCAAAGCATTTTGCAAGAACCAAGCAATTAACAAGCCATTGGCTCAGAGGGCAATAAGCTCATAAGAACGCAAGGCAGAAACACATCCTGCTTGCAAAGTTGGGGCAAATTAAGCACTGAAGCAGACATACTCTCAGGAAATCCCAAGAGTGCAACGCACGTTCAGGAGGCCATTGGTT |
| >GS_C88a |
| AATCAATGGCCTCCTGAACGTGCGTTGCACTCTTGGGATTTCCTGAGAGTATGCCTGCTT |
| CAGCGCTTAACTTGCCCCAACTTTGCAAGCATTCTCCTTGCGTTCCTATGAGCTATTGCC |
| CTCTGAGCCAATGGCTTGTTAATTGCTTGGTTCTTGCAAAATGCTTTGCGTGCTGTTATT |
| CAAGTTTCTACCTTCGTGGTTTTACTTGAGTGACGCTGCTCATGCTTGCAACCGCTGGGA |
| TGCAGGTGCATGCCTCTAGCATGAAGTCAGACAA |
| >GS_C89 |
| AACCAATGGCCTCCTGAACGTGCGTTGCACTCTTGGGATTTCCTGAGAGTATGTCTGCTT |
| CAGTGCTTAACTTGCCCCAACTTTGCAAGCAGGATGTGTTTCTGCCTTGCGTTCTTATGA |
| GCTATTGCCCTCTGAGCCAATGGCTTGTTAATTGCTTGGTTCTTGCAAAATGCTTTGCGC |
| GCTGTTATTCAGGTTTCTACCTTCGTGGTTTTACTTGAGTGATGCTGCTCATGCTTGCAA |
| CCGCTGGGATGCAGGGTGCATGCCTCTAGCATGAAGTCAGACAA |
| >GS_C90 |
| GCTTCAGTGCTTAACTTGCCCCAACTTTGCAAGCAGGATGTGTTTCTGCCTTGCGTTCTT |
| ATGAGCTATTGCCCTCTGCGCCAATGGCTTGTTAATTGCTTGGTTCTTGCAAAATGCTTT |
| GCGCGCTGTTATTCAAGTTTCTACCTTCAAGTGGTTTTACTTGAGTGACGCTGCTCATGC |
| TTGCAACCGCTGGGATGCAGGTGCATGCCTCTAGCATGAAGTCAGACAA |
| >GS_C91 |
| AACCAATGGCCTCCTGAACGTGCGTTGCACTCTTGGGATTTCCTGAGAGTATGTCTGCTT |
| CAGTGCTTAACTTGCCCCAACTTTGCAAGCAGGATGTGTTTCTGCCTTGCGTTCTTATGA |
| GCTATTGCCCTCTGCGCCAATGGCTTGTTAATTGCTTGGTTCTTGCAAAATGCTTTGCGC |
| GCTGTTATTCAAGTTTCTACCTTCGCGGTTTTACTTGAGTGACGCTGCTCATGCTTGCAA |
| CCGCTGGGATGCAGGTGCATGCCTCTAGCATGAAGTCAGACAA |
| >GS_C91a |
| AACCAATGGCCTCCTGAACGTGCGTTGCACTCTTGGGATTTCCTGAGAGTATGTCTGCTT |
| CAGTGCTTAACTTGCCCCAACTTTGCAAGCAGGATGTGTTTCTGCCTTGCGTTCTTATGA |
| GCTATTGCCCTCTGCGCCAATGGCTTGTTAATTGCTTGGTTCTTGCAAAATGCTTTGCGC |
| GCTGTTATTCAAGTTTCTACCTTCGCGGCTTTACTTGAGTGACGCTGCTCATGCTTGCAA |
| CCGCTGGGATGCAGGTGCATGCCTCTAGCATGAAGTCAGACAA |
| >GS_C91b |
| AACCAATGGCCTCCTGAACGTGCGTTGCACTCTTGGGATTTCCTGAGAGTATGTCTGCTT |
| CAGTGCTTAACTTGCCCCAACTTTGCAAGCAGGATGTGTTTCTGCCTTGCGTTCTTATGA |
| GCTATTGCCCTCTGCGCCAATGGCTTGTTAATTGCTTGGTTCTTGCAAAATGCTTTGCGC |
| GCTGTTATTCAAGTTTCTACCTTCGCGATTTTACTTGAGTGACGCTGCTCATGCTTGCAA |
| CCGCTGGGATGCAGGTGCATGCCTCTAGCATGAAGTCAGACAA |
| >GS_C91c |
| AACCAATGGCCTCCTGAACGTGCGTTGCACTCTTGGGATTTCCTGAGAGTATGTCTGCTT |
| CAGTGCTTAACTTGCCCCAACTTTGCAAGCAGGATGTGTTTCTGCCTTGCGTTCTTATGA |
| GCTATTGCCCTCTGCGCCAATGGCTTGTTAATTGCTTGGTTCTTGCAAAATGCTTTGCGC |
| GCTGTTATTCGAGTTTCTACCTTCGCGGTTTTACTTGAGTGACGCTGCTCATGCTTGCAA |
| CCGCTGGGATGCAGGTGCATGCCTCTAGCATGAAGTCAGACAA |
| >GS_C91d |
| AACCAATGGCCTCCTGAACGTGCGTTGCACTCTTGGGATTTCCTGAGAGTATGTCTGCTT |
| CAGTGCTTAACTTGCCCCAACTTTGCAAGCAGGATGTGTTTCTGCCTTGCGTTCTTATGA |
| GCTATTGCCCTCTGCGCCAATGGCTTGTTAATTGCTTGGTTCTTGCAAAATGCTTTGCGC |
| GCTGTTATTCTAGTTTCTACCTTCGCGGTTTTACTTGAGTGACGCTGCTCATGCTTGCAA |
| CCGCTGGGATGCAGGTGCATGCCTCTAGCATGAAGTCAGACAA |
| >GS_C92 |
| AACCAATGGCCTCCTGAACGTGCGTTGCACTCTTGGGATTTCCTGAGAGTATGTCTGCTT |
| CAGTGCTTAACTTGCCCCAACTTTGCAAGCAGGATGTGTTTCTGCCTTGCGTTCTTATGA |
| GCTATTGCCCTCTGCGCCAATGGCTTGTTAATTGCTCGGTTCTTGCAAAATGCTTTGCGC |
| GCTGTTATTCAAGTTTCTACCTTCGTGGTTTTGCTTGAGTGACGCTGCTCATGCTTGCAA |
| CCGCTGGGATGCAGGTGCATGCCTCTAGCATGAAGTCAGACAA |
| >GS_C92a |
| AACCAATGGCCTCCTGAACGTGCGTTGCACTCTTGGGATTTCCTGAGAGTATGTCTGCTT |
| CAGTGCTTAACTTGCCCCAACTTTGCAAGCAGGATGTGTTTCTGCCTTGCGTTCTTATGA |
| GCTATTGCCCTCTGCGCCAATGGCTTGTTAATTGCTCGGTTCTTGCAAAATGCTTTGCGC |
| GCTGTTATTCAAGTTTCTACCTTCGTGGTTTTGCTTGAGTGACGCTGCTCATGCTTGCAA |
| CTGCTGGGATGCAGGTGCATGCCTCTAGCATGAAGTCAGACAA |
| >GS_C93_(type_1) |
| AACCAATGGCCTCCTGAACGTGCGTTGCACCCTTGGGATTTCCTGAGAGTATGTCTGCTT |
| CAGTGCTTAACTTGCCCCAACTTTGCAAGCAGGATGTGTTTCTGCCTTGCGTTCTTATGA |
| GCTATTGCCCTCTGAGCCAATGGCTTGTTAATTGCTTGGTTCTTGCAAAATGCTTTGCGC |
| GCTGTTATTCAAGTTTCTACCTTCGTGGTTTTACTTGAGTGACGCTGCTCATGCTTGCAA |
| CCGCTGGGATGCAGGTGCATGCCTCTAGCATGAAGTCAGACAA |
| >GS_C93_(type_2) |
| AACCAATGGCCTCCTGAACGTGCGTTGCACTCTTGGGATTTCCTGAGAGTATGTCTGCTT |
| CAGTGCTTAACTTGCCCCAACTTTGCAAGCAGGATGTGTTTCTGCCTTGCGTTCTTATGA |
| GCTATTGCCTTTTGCGCCAATGGCTTGTTAATTGCTTGGTTCTTGCAAAATGCTTTGCGC |
| GCTGTTATTCAAGTTTCTACCTTCGCGGTTTTACTTGAGTGACGCTGCTCATGCTTGCAA |
| CCGCTGGGATGCAGGTGCATGCCTCTAGCATGAAGTCAGACCA |
| >GS_C93a |
| AACCAATGGCCTCCTGAACGTGCGTTGCACTCTTGGGATTTCCTGAGAGTATGTCTGCTT |
| CAGTGCTTAACTTGCCCCAACTTTGCAAGCAGGATGTGTTTCTGCCTTGCGTTCTTATGA |
| GCTATTGCCTTTTGCGCCAATGGCTTGTTAATTGCTTGGTTCTTGCAAAATGCTTTGCGC |
| GCTGTTATTCAAGTTTCTACCTTCGCGGTTTTACTTGAGTGACGCTGCTCATGCTTGCAA |
| CCGCTGGGATGCAGGTGCATGCCTCTAGCATGAAGTCAGACAA |
| >GS_C94 |
| AACCAATGGCCTCCTGAACGTGCGTTGCACTCTTGGGATTTCCTGAGAGTATGTCTGCTT |
| CAGTGCTTAACTTGCCCCAACTTTGCAAGCAGGATGTGTTTCTGCCTTGCGTTCTTATGA |
| GCCATTGCCCTCTGAGCCAATGGCGTGTTAATTGCTTGGTTCTTGCAAAATGCTTTGCGC |
| GCTGTTATTCAAGTTTCTACCTTCGTGGTTTTACTTGAGTGACGCTGCTCATGCTTGCAA |
| CCGCTGGGATGCAGGTGCATGCCTCTAGCATGAAGTCAGACAA |
| >GS_C94a |
| AACCAATGGCCTCCTGAACGTGCGTTGCACTCTTGGGATTTCCTGAGAGTATGTCTGCTT |
| CAGTGCTTAACTTGCTCCAACTTTGCAAGCAGGATGTGTTTCTGCCTTGCGTTCTTATGA |
| GCCATTGCCCTCTGAGCCAATGGCGTGTTAATTGCTTGGTTCTTGCAAAATGCTTTGCGC |
| GCTGTTATTCAAGTTTCTACCTTCGTGGTTTTACTTGAGTGACGCTGCTCATGCTTGCAA |
| CCGCTGGGATGCAGGTGCATGCCTCTAGCATGAAGTCAGACAA |
| >GS_C96 |
| AACCAATGGCCTCCTGAACGTGCGTTGCACTCTTGGGATTTCCTGAGAGTATGTCTGCTT |
| CAGTGCTTAACTTGCCCCGACTTTGCAAGCACTCTTCTGCCTTGCGTTCTTATGAGCTAT |
| TGCCCTCTCTGAGCCAATGGCTTGTTAATTGCTTGGTTCTTGCAAAATGCTTTGCGCGCT |
| GTTATTCAAGTTTCTACGTTCGTGGTTTTACTTGAGTGATGCTGCTCATGCTTGCAACCG |
| CTGGGATGCAGGTGCATGCCTCTAGCATGAAGTCAGACAA |
| >GS_C101 |
| AACCAATGGCCTCCTGAACGTGCGTTGCACTCTTGGGATTTCCTGAGAGTATGTCTGCTT |
| CAGTGCTTAACTTGCCCCAACTTTGCAAGCAGGATGTGTTTCTGCCTTGCGTTCTTATGA |
| GCTATTGCCCTCTGAGCCAATGGCTTGTTAATTGCTTGGTTCTTGCAAAATGCTTTGCGC |
| GCTGTTATTCAAGTTTCTACCTTCGTGGTTTTACTTGAGTGATGCTGCTCATGCTTGCAA |
| CCGCTGGGATGCAGGTGCATGCCTCTAGCATGAACTCAGACAA |
| >GS_C105 |
| AACCAATGGCCTCCTGAACGTGCGTTGCACTCTTGGGATTTCCTGAGAGTATGTCTGCTT |
| CAGTGCTTCACTTGCCCCAACTTTGCAAGCAGGATGTGTTTCTGCCTTGCGTTCTTATGA |
| GCTATTGCCCTCCAATGGCTTGTTAATTGCTTGGTTCTTGCAAAATGCTTTGCGCGCTGT |
| TAATTCAGGTTTCTACCTTCGTGGTTTTACTTGAGTGACGCGGCTCATGCTTGCAACCGC |
| TGGGATGCAGGTGCATGCCTCTAGCATGAAGTCAGACAA |
| >GS_C105a |
| AACCAATCGCCTCCTGAACGTGCGTTGCACTCTTGGGATTTCCTGAGAGTATGTCTGCTT |
| CAGTGCTTCACTTGCCCCAACTTTGCAAGCAGGATGTGTTTCTGCCTTGCGTTCTTATGA |
| GCTATTGCCCTCCAATGGCTTGTTAATTGCTTGGTTCTTGCAAAATGCTCCTCGCTGTTA |
| ATTCAGGTTTCTACCTTCGTGGTTTTACTTGAGTGACGCGGCTCATGCTTGCAACCGCTG |
| GGATGCAGGTGCATGCCTCTAGCATGAAGTCAGACAA |
| >GS_C106 |
| AACCAATGGCCTCCTGAACGTGCGTTGCACTCTTGGGATTTCCTGAGAGTATGTCTGCTT |
| CAGTGCTTAACTTGCCCCAACTTTTGCAAGCAGGATGTGTTTCTGCCTTGCGTTCTTATG |
| AGCTATTGCCCTCTGAGCCAATGGCTTGTTAATTGCTTGGTTCTTGCAAAATGCTTTTGC |
| GCGCTGTTATTCAGGTTTCTACCTTCGTGGTTTTACTCGAGTGATGCTGCTCGTGCTTGC |
| AACCACTGGGATGCAGGTGCATGCCTCTAGCATGAAGTCAGACAA |
| >GS_C106a |
| AACCAATGGCCTCCTGAACGTGCGTTGCACTCTTGGGATTTCCTGAGAGTATGTCTGCTT |
| CAGTGCTTAACTTGCCCCAACTTTGCAAGCAGGATGTGTTTCTGCCTTGCGTTCTTATGA |
| GCTATTGTCCTCTGAGCCAATGGCTTGTTAATTGCTTGGTTCTTGCAAAATGCTTTTGCG |
| CGCTGTTATTCAGGTTTCTACCTTCGTGGTTTTACTTGAGTGATGCTGCTCGTGCTTGCA |
| ACCACTGGGATGCAGGTGCATGCCTCTAGCATGAAGTCAGACAA |
| >GS_C107 |
| AAGCAATGGCCTCCTGAACGTGCGTTGCACTCTTGGGATTTCCTGAGAGTATGTCTGCTT |
| CAGTGTTTAACTTGCCCCAACTTTGCAAGCAGGATGTGTTTCTGCCTTGCGTTCTTATGA |
| GCTATTGCCCTCTGAGCCAATGGCTTGTTAATTGCTTGGTTCTTGCAACATGCTTTGCGC |
| GCGCTGTTATTCAAGTTTCTACCTTCGTGGTTTTACTTGAGTGACGCTGCTCATGCTTGC |
| AACCGCTGGGATGCAGGTGCATGCCTCTAGCATGAAGTCAGACAA |
| >GS_C107a |
| AACCAATGGCCTCCTGAACGTGCGTTGCACTCTTGGGATTTCCTGAGAGTATGTCTGCTT |
| CAGTGTTTAACTTGCCCCAACTTTGCAAGCAGGATGTGTTTGTGCCTTGCGTTCTTATGA |
| GCTATTGCCCTGTGAGCCAATGGCTTGTTAATTGCTTGGTTCTTGCAAAATGCTTTGCGC |
| GCTGTTATTCAAGTTTCTACCTTCGTGGTTTTACTTGAGTGACGCTGCTCATGCTTGCAA |
| CCGCTGGGATGCAGGTGCATGCCTCTAGCATGAAGTCAGACAA |
| >GS_C107b |
| AACCAATGGCCTCCTGAACGTGCGTTGCACTCTTGGGATTTCCTGAGAGTATGTCTGCTT |
| CAGTGTTTAACTTGCCCCAACTTTGCAAGCAGGATGTGTTTGTGCCTTGCGTTCTTATGA |
| GCTATTGCCCTCTGAGCCAATGGCTTGTTAATTGCTTGGTTCTTGCAACATGCTTTGCGC |
| GCGCGCTGTTATTCAAGTTTCTACCTTCGTGGTTTTACTTGAGTGACGCTGCTCATGCTT |
| GCAACCGCTGGGATGCAGGTGCATGCCTCTAGCATGAAGTCAGACAA |
| >GS_C110 |
| AACCAATGGCCTCCTGAACGTGCGTTGCACTCTTGGGATTTCCTGAGAGTATGCCTGCTT |
| CAGCGCTTAACTTGCCCCAACTTTGCAAGCATTCTCCTTGCGTTCCTATGAGCTATTGCC |
| CTCTGAGCCAATGGCTTGTTAATTGCTTGGTTCTTGCAAAATGCTTTGCGTGCTGTTATT |
| CAAGTTTCTACCTTCGTGGTTTTACTTGAGTGACGCTGCTCATGCTTGCAACCGCTGGGA |
| TGCAGGTGCATGCCTCTAGCATGAAGTCAGACAA |
| >GS_C112 |
| AACCAATGGCCTCCTGAACGTGCGTTGCACTCTTGGGATTTCCTGAGAGTAGGTCTGCTT |
| CAGTGCTTAACTTGCCCCAACTTTGCAAGCAGGATGTGTTTCTGCCTTGCGTTCTTATGA |
| GCTATTGCCCTCTGAGCCAATGGCTTGTGAATTGCTTGGTTCTTGCAAAATGCTTTGCGC |
| GCTGTTATTCAGGTTTCTACCTTCGTGGTTTTACTTGAGTGACGCTGCTCGTGCTTGCAA |
| CCGCTGGGATGCAGGTGCATGCCTCTAGCATGAAGTCAGACAA |
| >GS_C113=C114 |
| AACCAATGGCCTCCTGAACGTGCGTTGCACTCTTGGGATTTCCTGAGAGTATGTCTGATT |
| CAGTGCTTAACTTACCCCAACTTTGCAAGCAGGATGTGTTTCTGCCTTGCGTTCTTATGA |
| GCTATTGCCCTCTGAGCCAATGGCTTGTTACTTGCTTGGTTCTTGCAAAATGCTTTGCGC |
| GCTGTTACTCAGGTTTCTACCTTCGTGGTTTTACTTGAGTGACGCTGCTCATGCTTGCAA |
| CCGCTGGGATGCAGGTGCATGCCTCTAGCATGAAGTCAGACAA |
| >GS_C115 |
| AACCAATGGCCTCCTGAACGTGCGTTGCACTCTTGGGATTTCCTGAGAGTATGTCTGCTT |
| CAGTGCTTAACTTGCCCCAACTTTGCAAGCAGGATGTGTTTCTGCCTTGCGTTCTTATGA |
| GCTATTGCCCTCTGAGCCAATGGCTTGTTAATTGCTTGGTTCTTGCAAAATGCTTTGCGC |
| GCTGTTATTCAAGTTTCTACCTTCGTGGTTTTACTTGACTGACGCTGCTCATGCTTGCAA |
| CCGCTGGGATGCAGGTGCATGCCTCTAGCATGAAGTCAGACAA |
| >GS_C115a |
| AACCAATGGCCTCCTGAACGTGCGTTGCACTCTTGGGATTTCCTGAGAGTATGTCTGCTT |
| CAGTGCTTAACTTGCCCCAACTTTGCAAGCAGGATGTGTTTCTGCCTTGCGTTCTTATGA |
| GCTATTGCCCTCTGAGCCAATGGCTTGTTAATTGCTTGGTTCTTGCAAAATGCTTTGCGC |
| GCTGTTATTCAAGTTTCTACCTTTGTGGTTTTACTTGACTGACGCTGCTCATGCTTGCAA |
| CCGCTGGGATGCAGGTGCATGCCTCTAGCATGAAGTCAGACAA |
| >GS_C116 |
| AACCAATGGCCTCCTGAACGTGCGTTGCACCCTTGGGATTTCCTGAGAGTATGTCTGCTT |
| CAGTGCTTAACTTGCCCCAACTTTGCAAGCAGGATGTGTTTCTGCCTTGCGTTCTTATGA |
| GCTATTGCCTTCTGCGCCAATGGCTTGTTAATTGCTTGGTTCTTGCAAAATGCTTTGCGC |
| GCTGTTATTCAAGTTTCTACCTTCCCGGTTTTACTTGAGTGACGCTGCTCATGCTTGCAA |
| CCGCTGGGATGCAGGTGCATGCCTCTAGCATGAAGTCAGACAA |
| >GS_C119 |
| AAGCAATGGCCTCCTGAACGTGCGTTGCACTCTTGGGATTTCCTGAGAGTATGTCTGCTT |
| CAGTGCTTAACTTGCCCCAACTTTGCAAGCAGGATGTGTTTCTGCCTTGCGTTCTTATGA |
| GCTATTGCCCTCTGAGCCAATGGCTTGTTAATTGCTTGGTTCTTGCAAAATGCTTTGCGC |
| GCTGTTATTCAAGTTTCTACCTTCGCGATTTTACTTGAGTGACGCTGCTCATGCTTGCAA |
| CCGCTGGGATGCAGGTGCATGCCTCTAGCATGAAGTCAGACAA |
| >GS_C120 |
| AACCAATGGCCTCCTGAACGTGCGTTGCACTCTTGGGATTTCCTGAGAGTATGTCTGCTT |
| CAGTGCTTAACTTGCCCCAACTTTGCAAGCAGGATGTGTTTCTGCCTTGCGTGCTTATGA |
| GCTATTGCCCTCTGAGCCAATGGTTTGTGAATTGCTTGGTTCTTGCAAAATGCTTTGCGC |
| GCTGTTATTCAGGTTTCTACCTTCGTGGTTTTACTTGAGTGACGCTGCTCATGCTTGCAA |
| CCGCTGGGATGCAGGTGCATGCCTCTAGCATGAAGTCAGACAA |
| >GS_C120a |
| AACCAATGGCGTCCTGAACGTGCGTTGCACTCTTGGGATTTCCTGAGAGTATGTCTGCTT |
| CAGTGCTTAACTTGCCCCAACTTTGCAAGCAGGATGTGTTTCTGCCTTGCGTGCTTATGA |
| GCTATTGCCCTCTGAGCCAATGGTTTGTGAATTGCTTGGTTCTTGCAAAATGCTTTGCGC |
| GCTGTTATTCAGGTTTCTACCTTCGTGGTTTTACTTGAGTGACGCTGCTCATGCTTGCAA |
| CCGCTGGGATGCAGGTGCATGCCTCTAGCATGAAGTCAGACAA |
| >GS_C123 |
| AACCAATGGTCTCCTCAACGTGCGTTGCACTCTTGGGATTTCCTGAGAGTATGTCTGCTT |
| CAGTGCTTAACTTGCCCCAACTTTGCAAGCAGATGTGTTTCTGCCTTGCGTTCTTATGAG |
| CTATTGCCCTCTGAGCCAATGGCTTGTTAATTGCTTGGTTCTTGCAAAATGCTTTGCGCG |
| CTGTTATTCAGGTTTCTGCCTTCGTGGTTTTACTTGAGTGACGCTGCTCATGCTTGCAAT |
| TGCTGGGATGCAGGTGCATGCCTCTAGCATGAAGTCAGACAA |
| >GS_C124 |
| AACCAATGGCCTCCTGAACGTGCGTTGCACTCTTGGGCTTTCCTGAGAGTATGTCTGCTT |
| CAGTGCTTAACTTGTCCCAACTTTGCAAGCAGGACGTGTTTCTACCTTGCATTCTTATGA |
| GCTATTGCCCTCTGAGCTAATGGCTTGTTAAATTGCTTGGTTCTTGCAAAATGCTTTGCG |
| CGCTGTTATTCAAGTTTCTACCTTCGTGGTTTTATTTGAGTGACGCTGCTCATGCTTGCA |
| ACCGCTGGGATGCAGGTGCATGCCTCTAGCATGAAGTCAGACAA |
| >GS_C125 |
| ACGTGCGTTGCACTCTCGGGATTTCCTGAGAGCATGTCTGCTTCAGTGCTTAGCTTGCCC |
| CAACTTTGCAAGCAGGATGTGTTTCTGCCTTGCGTTCTTATGAGTTATTGCCCTCTGAGG |
| CAATGGCTTGTTAATTGTTTGGTTCTTGCAAAATGCTTTGCGCGCTGTTATTCAGGTTTC |
| TACCTTCGTGGTTTTACTTGAGTGACGCTGCTCATGCTTGCAACCGCTGGGATGTAGGTG |
| CATGCCTCTAGCATGAAGTCAGACAA |
| >GS_C126 |
| ACGTGCGTTGCACTCTTGGGATTTCCTGAGAGCATGTCTGCTTCAGTGCTTAGCTTGCCC |
| CAACTTTGCAAGCAGGATGTGTTTCTGCCTTGCGTTCTTATGAGTTATTGCCCTCTGAGG |
| CAATGGCTTGTTAATTGTTTGGTTCTTGCAAAATGCTTTGCGCGCTGTTATTCAGGTTTC |
| TACCTTCGTGGTTTTACTTGAGTGACGCTGCTCATGCTTGCAACCGCTGGGATGTAGGTG |
| CATGCCTCTAGCATGAAGTCAGACAA |
| >GS_C128 |
| AATCAATGGCCCCCTGAACGGGTGTTGCACTCTTGGGATTTCCTGAGAGTATGTCTGCTT |
| CAGTGCTTAACTTGCCCCAACTTTGCAAGCAGGATGTGTTTCTGCCTTGCGTACTTATGA |
| ACTATTGCCCTCTGAGTCAATGGCTTGTTAATTGCTTGGTTCTTGCAAAATGCTTTGTGC |
| GCTGTTATTCACGTTTCTACCTTCGTGGTTTTACTTGAGTGACGCTGCTCATGCTTGCAA |
| CCGCTGGGATGCTGGGATGCAGGTGCATGCCTCTAGCATGAAGTCAGACAA |
| >LJ_C160 |
| GTCTGACTTCATGCTAGAGGCATGCACCTGCATCCCAGCGGTTGCAAGCATGAGCAGCGTCACTCAAGTAAAACCACGAAGGTAGAAACTTGAATAACAGCGCGCAAAGCATTTTGCAAGAACCAAGCAATTAACAAGCCATTGGCTCAGAGGGCAATAGCTCATAAGAACGCAAGGCAGAAATGCTTGCAAAGTTGGGGCAAGTTAAGCACTGAAGCAGACATACTCTCAGGAAATCCCAAGAGTGCAACGCACGTTCAGGAGGCCATTGTTT |
| >LJ_C161 |
| GTCTGACTTCATGCTAGAGGCATGCACCTGCATCCCAGCGGTTGCAAGCATGAGCAGCGTCACTCAAGTAAAACCACGAAGGTAGAAACCTGAATAACAGCGCGCAAAGCATTTTGCAAGAACCAAGCAATTAACAAGCCATTGGAGGGCAATAGCTCATAAGAACGCAAGGCAGAAACACATCCTGCTTGCAAAGTTGGGGCAAGTTAAGCACTGAAGCAGACATACTCTCAGGAAATCCCAAGAGTGCAACGCACGTTCAGGAGGCCATTGGTTCACGGAGTTCTGCAATTCA |
| >LJ_C163 |
| GAGCAGCGTCACTCAAGTAAAACCAAGAAGGTAGAAACTTGAATAACAGCGCGCAAAGCATTTTGCAAGAACCAAGCAATTAACAAGCCATTGGCTCAGAGGGCAATAGCTCATAAGAACGCAAGGCAGAAACACATCTTGCAAAGTTGGGGCAAGTTAAGCACTGAAGCAGACATACTCTCAGGAAATCCCAAGAGTGCAACGCACGTTCAGGAGGCCATTGGTT |
| >LJ_C163a |
| GTCTGACTTCATGCTAGAGGCATGCACCTGCATCCCAGCGGTTGCAAGCATGAGCAGCGTCACTCAAGTAAAACCAAGAAGGTAGAAACTTGAATAACAGCGCGCAAAGCATTTTGCAAGAACCAAGCAATTAACAAGCCATTGGCTCAGAGGGCAATAGCTCATAAGAACACAAGGCAGAAACACATCTTGCAAAGTTGGGGCAAGTTAAGCACTGAAGCAGACATACTCTCAGGAAATCCCAAGAGTGCAACGCACGTTCAGGAGGCCATTGGTT |
| >LJ_C163b |
| GTCTGACTTCATGCTAGAGGCATGCACCTGCATCCCAGCGGTTGCAAGCATGAGCAGCGTCACTCAAGTAAAACCAAGAAGGTAGAAACCTGAATAACAGCGCGCAAAGCATTTTGCAAGAACCAAGCAATTAACAAGCCATTGGCTCAGAGGGCAATAGCTCATAAAAACGCAAGGCAGAAACACATCTTGCAAAGTTGGGGCAAGTTAAGCACTGAAGCAGACATACTCTCAGGAAATCCCAAGAGTGCAACGCACGTTCAGGAGGCCATTGGTTCACGGAGTTCTGCAATTCAC |
| >GS_D1 |
| AACCAATGGCCCCCTGAACGCGCATTGCACTCTTGGGACTTCCTGAGAGTATGTTTGCTT |
| CAGTGCTTATTTTACCTCCTTGCAAGGTTCTGTCGCAACCTTGTGCCCTGGCCAGCCATG |
| GGTTAACTTGCCCATGGCTTGCTGAGTAGTGATCTTTTAGAGCAAGCTCTGGCACGCTGT |
| TGTTTGAGGCAGCCTATATTGAGGCTATTTCAAATGACGTTGCTACAAGCTTGATGTGTC |
| CTTCTGCGCCGTTGCGCATCCCATAGCATGA |
| >GS_D1a |
| AACCAATGGCCCCCTGAACGCGCATTGCACTCTTGGGACTTCCTGAGAGTATGTTTGCTT |
| CAGTGCTTATTTTACCTCCTTGCAAGGTTCTGTCGCAACCTTGTGCCCTGGCCAGCCACG |
| GGTTAACTTGCCCATGGCTTGCTGAGTAGTGATCTTTTAGAGCAAGCTCTGGCACGCTGT |
| TGTTTGAGGCAGCCTATATTGAGGCTATTTCAAATGACGTTGCTACAAGCTTGATGTGTC |
| CTTCTGCGCCGTTGCGCATCCCATAGCATGA |
| >GS_D1.1 |
| AACCAATGGCCTCCTGAACGTGCATTGCACTCTTGGGATTTCCTGAGAGTATGTTTGCTT |
| CAGTGCTTAGTTTGGTCAACTTTGTTTGGATCCTGCTCCTGGGAACTGAGCGCCTCTGTG |
| AATCATTGGAGCAAACAGGACTTTGTCTCTGTTTTTTTGCCAATGGTTTGCTTGTCTCTA |
| CAATCCTTTGTGTAGCGCAAGCATATGTGCGTTGTTGTTTGATACATTTATCTCTTTGTA |
| GCATAAATGCTTTCTGCACGAAGCACATTGATATGCTTGCTACTGCTACTGTGCTGCGGA |
| GAGTTGGTTTTTGCATGTGTACCAGGTGTATAGTATGCTTATACTCTCTTGCACAACCCA |
| TAGCATGA |
| >GS_D1.2 |
| AACCAATGGCCTCCTGAACGCGCATTGCACTCTTGGGATTTCCTGAGAGTATGTTTGCTT |
| CAGTGCTTAGTTTGGTCAACTTTGTTTGGATCCTGCTTCTGGGAACCAAGCACCTCTGTG |
| AATCATTGGAACAAACAGGACTTCGTCTCTGTTTTGCCAATGGTTTGCTTGTCTCTTCAA |
| TCCTTTGTGTAGCACAAGCATATGTGCGTTGTTGTTTGATACATTTATATCTCTTTGTAG |
| TATAGATGCTATCTGCACGAAGCACATCTGATATGCTTGCTACTGCTATTGTGCTGCTGA |
| GAATTCTTATATGCATATGTACCATGTGTATATGATGTCTATATGATTTCTCTCCCTTGC |
| ACAACCCATAGCATGA |
| >GS_D1.6 |
| AACCAATGGCCCCCTGAACGCGCATTGCACTCTTGGGACTTCCTGAGAGTATGTTTGCTT |
| CAGTGCTTATTTTACCTCCTTGCAAGGTTCTGTCGCAACCTTGTGCCCTGGCCAGCCATG |
| GGTTAACTTGCCCATGGCTTGCTGAGTAGTGATCTTTTAGAGCAAGCTCTGGCACGCTGT |
| TGTTTGAGGCAGCCTATATTGAGGCTATTTCAAATGACGTTGCTACAAGCTTGATGTGTC |
| CTTCTGCGCCGTTGCGCAACCCATAGCATGA |
| >GS_D2 |
| AACCAATGGCCCCCTGAACGCGCATTGCACTCTTGGGACTTCCTGAGAGTATGTTTGCTT |
| CAGTGCTTATTTTACCTCCTTGCAAGGACCTTGTGCCCTGGCCAGCCACGGGTTAACTTG |
| CCCATGGCTTGCTGAGTAGTGATCTTTTAGAGCAAGCTCTGGCACGCTGTTGTTTGAGGC |
| AGCCTATATTGAGGCTATTTCAAATGACGTTGCTACAAGCTTGATGTGTCCTTCTGCGCC |
| GTTGCGCATCCCATAGCATGA |
| >GS_D2.2 |
| AACCAATGGCCCCGTGAACGCGCATTGCACTCTTGGGACTTCCTGAGAGTATGTTTGCTT |
| CAGTGCTTATTTTACCTCCTTGCAAGGTTCTGTCGCAACCTTGTGCCCTGGCCAGCCACG |
| GGTTAACTTGCCCATGGCTTGCTGAGTAGTGATCTTTTAGAGCAAGCTCTGGCACGCTGT |
| TGTTTGAGGCAGCCTATATTGAGGCTATTTCAAATGACGTTGCTACAAGCTTGATGTGTC |
| CTTCTGCGCCGTTGCGCATCCCATAGCATGA |
| >GS_D3 |
| AACCAATGGCCCCCTGAACGCGCATTGCACTCTTGGGACTTCCTGAGAGTATGTTTGCTT |
| CAGTGCTTATTTTACCTCTTTGCAAGGTTCTGTCGCAACCTTGTGCCCTGGCCAGCCATG |
| GGTTAACTTGCCCATGGCTTGCTGAGTAGTGATCTTTTAGAGCAAGCTCTGGCACGCTGT |
| TGTTTGAGGCAGCCTATATTGAGGCTATTTCAAATGACGTTGCTACAAGCTTGATGTGTC |
| CTTCTGCGCCGTTGCGCATCCCATAGCATGA |
| >GS_D4 |
| AACCAATGGCCCCCTGAACGCGCATTGCACTCTTGGGACTTCCTGAGAGTATGTTTGCTT |
| CAGTGCTTATTTTACCTCCTTGCAAGGTTCTGTGCGCAACCTTGTGCCCTGGCCAGCCAC |
| GGGTTAACTTGCCCATGGCTTGCTGAGTAGTGATCTTTTAGAGCAAGCTCTGGCACGCTG |
| TTGTTTGAGGCAGCCTATATTGAGGCTATTTCAAATGACGTTGCTACAAGCTTGATGTGT |
| CCTTCTGCGCCGTTGCGCATCCCATAGCATGA |
| >GS_D5 |
| AACCAATGGCCCCCTGAACGCGCATTGCACTCTTGGGACTTCCTGAGAGTATGTTTGCTT |
| CAGTGCTTGTTTTACCTCCTTGCAAGGTTCTGTCGCAACCTTGTGCCCTGGCCAGCCACG |
| GGTTAACTTGCCCATGGCTTGCTGAGTAGTGATCTTTTAGAGCAAGCTCTGGCACGCTGT |
| TGTTTGAGGCAGCCTATATTGAGGCTATTTCAAATGACGTTGCTACAAGCTTGATGTGTC |
| CTTCTGCGCCGTTGCGCATCCCATAGCATGA |
| >GS_D6 |
| AACCAATGGCCCCCTGAACGCGCATTGCACTCTTGGGACTTCCTGAGAGTATGTTTGCTT |
| CAGTGCTTATTTTACCTCCTTGCAAGGTTCTGTCGCAACCTTGTGCCCTGGCCAGCCACG |
| CGTTAACTTGCCCATGGCTTGCTGAGTAGTGATCTTTTAGAGCAAGCTCTGGCACGCTGT |
| TGTTTGAGGCAGCCTATATTGAGGCTATTTCAAATGACGTTGCTACAAGCTTGATGTGTC |
| CTTCTGCGCCGTTGCGCATCCCATAGCATGA |
| >GS_D7 |
| AACCAATGGCCCCCTGAACGTGCATTGCACTCTTGGGACTTCCTGAGAGTATGTTTGCTT |
| CAGTGCTTGTTTTACCTCCTTGCAAGGTTCTGTCGCAACCTTGTGCCCTGGCCAGCCACG |
| GGTTAACTTGCCCATGGCTTGCTGAGTAGTGATCTTTTAGAGCAAGCTCTGGCACGCTGT |
| TGTTTGAGGCAGCCTATATTGAGGCTATTTCAAATGACGTTGCTACAAGCTTGATGTGTC |
| CTTCTGCGCCGTTGCGCATCCCATAGCATGA |
| >GS_D8 |
| AACCAATGGCCCCCTGAACGCGCATCGCACTCTTGGGACTTCCTGAGAGTATGTTTGCTT |
| CAGTGCTTATTTTACCTCCTTGCAAGGTTCTGTCAACCTTGTGCCCTGGCCAGCCACGGG |
| TTAACTTGCCCATGGCTTGCTGAGTAGTGATCTTTTAGAGCAAGCTCTCTGGCACGCTGT |
| TGTTTGAGGCAGCCTGTATTGAGGCTATTTCAAATGACGTTGCTACAAGCTTGATGTGTC |
| CTTCTGCGCCGTTGCGCATCCCATAGCATGA |
| >GS_D9 |
| AACCAATGGCCCCCTGAATGCGCATTGCACTCTTGGGACTTCCTGAGAGTATGTTTGCTT |
| CAGTGCTTATTTTACCTCCTTGCAAGGTTCTGTCGCAACCTTGTGCCCTGGCCAGCCACG |
| GGTTAACTTGCCCATGGCTTGCTGAGTAGTGATCTTTTAGAGCAAGCTCTGGCACGCTGT |
| TGTTTGAGGCAGCCTATATTGAGGCTATTTCAAATGACGTTGCTACAAGCTTGATGTGTC |
| CTTCTGCGCCGTTGCGCATCCCATAGCATGA |
| >GS_D10 |
| AACCAATGGCCCCCTGAATGCGCATTGCACTCTTGGGACTTCCTGAGAGTATGTTTGCTT |
| CAGTGCTTATTTTACCTCCTTGCAAGGTTCTGTCGCAACCTTGTGCCCTGGCCAGCCATG |
| GGTTAACTTGCCCATGGCTTGCTGAGTAGTGATCTTTTAGAGCAAGCTCTGGCACGCTGT |
| TGTTTGAGGCAGCCTATATTGAGGCTATTTCAAATGACGTTGCTACAAGCTTGATGTGTC |
| CTTCTGCGCCGTTGCGCATCCCATAGCATGA |
| >GS_D11 |
| AACCAATGGCCCCCTGAACGCGCATTGCACTCTTGGGACTTCCTGAGAGTATGTTTGCTT |
| CAGTGCTTATTTTACCTCCTTGCAAGGTTCTGTCGCAACCTTGTGCCCTGGCCAGCCATG |
| GGTTAACTTGCCCATGGCTTGCTGAGTAGTGATCTTTTAGAGCAAGCTCTGGCACGCTGT |
| TGTTTGAGGCAGCCTATATTGAGGCTATTTCAAATGACAAGCTACAAGCTTGATGTGTCC |
| TTCTGCGCCGTTGCGCATCCCATAGCATGA |
| >LJ_D17 |
| GTTTGACTTCATGCTATGGGATGCGCAACGGCGCAGAAGGACACATCAAGCTTGTAGCAACGTCATTTGAAATAGCCTCAATATAGGCTGCCTCAAACAACAGCGTGCCAGAGCTTGCTCTAAAAGATCACTACTCAGCAAGCCATGGGCAAGTTAACCCATGGCTGGCCAGGGCACAAGGTTGCGACAGAACCTTGCAAGGAGGTAAAATAAGCACTGAAGCAAACATACTCTCAGGAAGTCCCAAGAGTGCAATGCACGTTCAGGGGGCCATTGGTT |
| >GS_E1 |
| AACCAATAGCACCCTGAACTCGCATTGCACTCTTGGGACACGCCTGAGAGTATGTCTGCT |
| TCAGTGCTTTTCATATCTTCGCAGTGCGGGCTTCCTGGAGAAGCCTTGAGCCTCTTTGTG |
| CGCTGCTGCATCAGAATTTGCAGCGGCGCGCTGAACACAAACCGGGAGGTAAGCTGGACT |
| GATTTGTCGCGCATCACTGGGCACGTGTGTCCGTTTTGGCCCAATCATGCCAGCCTGCCA |
| AGCAATTGGTGCTCAAATACCAATCTTAGCATGAAGTCGAGACAA |
| >GS_F1 |
| AACCAATGGCCTCCTGAACGTACGTTGCACTCTTGGGATTTCCTGAGAGTATGTCTGCTT |
| CAGTGCTTAGCTTGCCCAATCTTGCGGATAGATTTTGTTTCTGTCTTGCGCCCCTGTGAG |
| CCATTGAAACTCTAGTCAATGGCTTATTGAATGAGTTGGTCTTGCAAAAGCTTTGCGCGA |
| TGCTATTCAAGATTCCACCTTGAAATGGTATTTCTTGAGTGACGCTGCTTATGCTTGCAA |
| CTGCTGGGATGCTAGCGCATGCCTCTAGCATGAAGTCAGACAAGTGAA |
| >GS_F2 |
| AGTCAATGGCCTCCTGAACGTACGTTGCACTCTTGGGGTTTCCTGAGAGTATGTCTGCTT |
| CAGTGCTTAGCTTGCCCAACCATGCAAGCAGTTTTGTCGTCTGCCTTGCGCTCCTACGAG |
| CCATTGTGACTACCAATGGCTTGTTGAATGTTCTTACTTGCATATGAAAGTTTTGCGCGC |
| TATTGTCCAAGTTTTACCTTCTCCGGTATTGCTTGAGTGACGCTGCTAACGCTTGCAACT |
| GCCGGGCTGCTGGTGCATGCCTCTAGCATGAAGTCAGGCAAGTGAA |
| >GS_F2a |
| AGTCAATGGCCTCCTGAACGTACGTTGCACTCTTGGGGTTTCCTGAGAGTATGTCTGCTT |
| CAGTGCTTAGCTTGCCCAACCATGCAAGCAGTTTTGTCGTCTGCCTTGCGCTCCTACGAG |
| CCATTGTGACTACCAATGGCTTGTTGAATRATCTTACTTGCATATGAAAGTTTTGCGCGC |
| TATTGTCCAAGTTTTACCTTCTCCGGTATTGCTTGAGTGACGCTGCTAACGCTTGCAACT |
| GCCGGGCTGCTGGTGCATGCCTCTAGCATGAAGTCAGGCAAGTGAA |
| >GS_F2b |
| AGTCAATGGCCTCCTGAACGTACGTTGCACTCTTGGGGTTTCCTGAGAGTATGTCTGCTT |
| CAGTGCTTAGCTTGCCCAACCACGCAAGCAGTTTTGTCGTCTGCCTTGCGCTCCTACGAG |
| CCATTGTGACTACCAATGGCTTGTTGAATGTTTTTACTTGCATATGAAAGTTTTGCGCGC |
| TATTGTTCAAGTTTTACCTTCGCCGGTATTGCTTGAGTGACGCTGCTAACGCTTGCAACT |
| GCTGGGCTGCTGGTGCATGCCTCTAGCATGAAGTCAGGCAAGTGAA |
| >GS_F3.1 |
| AATCAATGGCCTCCTGAACGTACGTTGCACTCTCGGGGTTTCCTGAGAGTATGTCTGCTT |
| CAGTGCTTAGCTTGCCCGACCTTGCACAAGCGGCTTTGTGTCTGCTTTGCGCTCTTATGA |
| GCCATTGGTTTGTGAAGCCTATGGCTCGTTAAATCGTTGTGTTTGCGAACGAAAAGCTTT |
| GCGCGCTGTTATTCGAGTAAAGTGCCCTTCGGGGTTCTGGGCTTGAGTGACGCTGCTCTT |
| GCTTGCAACTGCCAGGCTGCTGTGGTGCACGCCTCTAGCATGAAGTCAGGCAAGTGAA |
| >GS_F3.1a |
| AATCAATGGCCTCCTGAACGTACGTTGCACTCTCGGGGTTTCCTGAGAGTATGTCTGCTT |
| CAGTGCTTAGCTTGCCCGACCTTGCACAAGCGGCTTTGAGTCTGCTTTGCGCTCTTATGA |
| GCCATTGGTTTGTGAAGCCTATGGCTCGTTAAATCGTTGTGTTTGCGAACGAAAAGCTTT |
| GCGCGCTGTTATTCGAGTAAAGTGCCCTTTGGGGTTCTGGGCTTGAGTGACGCTGCTCTT |
| GCTTGCAACTGCCAGGCTGCTGTGGTGCACGCCTCTAGCATGAGGTCAGGCAAGTGAA |
| >GS_F3.2 |
| AATCAATGGCCTCCTGAACGTACGTTGCACTCTCGGGGTTTCCTGAGAGTATGTCTGCTT |
| CAGTGCTTAGCTTGCCCAACCTTGCAAAAGCGGCTTTGTGTCTGCTTTGTGCCCTTATGA |
| GCCATTGGTTTCCAACCAATGGCTTGTTAAATCATTGTGTTTGCAAATGAAAAGCTTTGC |
| GCGCTGTTATTCAAGTAAGTACCCTTCAGGGTTCTAAACTTGAGTGACGCTGCTCTTGCT |
| TGCAACTGCCAGGCTGCTGGTGCATGCCTCTAGCATGAAGTCAGGCAAGCGAA |
| >GS_F3.2a |
| AATCAATGGCCTCCTGAACGTACGTTGCACTCTCGGGGTTTCCTGAGAGTATGTCTGCTT |
| CAGTGCTTAGCTTGCCCAACCTTGCAAAAGCGGCTTTGTGTCTGCTTTGTGCTCTTATGA |
| GCCATTGGTTTCCAGCCAATGGCTTGTTGAATCATTGTGTTTGCAAATGACAAGCTTTGC |
| GCGCTGTTATTCAAGTAAGTTCCCTTCAGGGTTCTAAACTTGAGTGACGCTGCTCTTGCT |
| TGCAACTGCCAGGCTGCTGGTGCATGCCTCTAGCATGAAGTCAGGCAAGCGAA |
| >GS_F3.3 |
| AATCAATGGCCTCCTGAACGTACGTTGCACTCTTGGGTTTTCCTGAGAGTATGTCTGCTT |
| CAGTGCTTAGCTTGCCCAACTTTGCAAGAAGCATTACTTGCTTCCTGCGCTCGTATGAGC |
| CATTTGCTTTTTTGCTGATGGCTTGTTGAGTAATAGGTGTTGCAAAAAAAAGCTTTGCGC |
| GCTGTTGTTCAAGTACGTACCCTTGACAGGTCAGCTTGAGTGACGCTGCTCGTGCTTGCA |
| ACTGCCAGGGTGCTGGCGCATGCCTCTAGCATGAAGTCAGGCAAGTGAA |
| >GS_F3.4 |
| AATCAATGGCCTCCTGAACGTACGTTGCACTCTTGGGTTTTCCTGAGAGTATGTCTGCTT |
| CAGTGCTTAGCTTGCCCAACTTTGCAGGAAACATTATTTGCTTTCTGCGCTTGTATGAGC |
| CATTTGCTTTTTAGCAGATGGCTCGTTGAGTAATCAGTGTTGCAAATAAAAGCTTTGCGC |
| GCTATTGTTCAAGCACGTACCTTTGACGGGTTAGCTTGAGTGACGCTGCTTGTGCTTGCA |
| ATTGCCAGGCTGCTGCCGCATGCCTCTAGCATGAAGTCAGGCAAGTGGA |
| >GS_F3.4a |
| AATCAATGGCCTCCTGAACGTACGTTGCACTCTTGGGTTTTCCTGAGAGTATGTCTGCTT |
| CAGTGCTTAGCTTGCCCAACTTTGCAGGAAACATTATTTGCTTTCTGCGCTTGTATGAGC |
| CATTTGCTTTTTAGCAGATGGCTCGCTGAGTAATCAGTGTTGCAAATAAAAGCTTTGCGC |
| GCTATTGTTCAAGCATGTACCTTTGACGGGTTAGCTTGAGTGACGCTGCTTGTGCTTGCA |
| ATTGCCAGGCTGCTGCCGCATGCCTCTAGCATGAAGTCAGGCAAGTGGA |
| >GS_F3.4b |
| AATCAATGGCCTCCTGAACGTACGTTGCACTCTTGGGTTTTCCTGAGAGTATGTCTGCTT |
| CAGTGCTTAGCTTGCCCAACTTTGCAGGAAACATTATTTGCTTTCTGCGCTTGTATGAGC |
| CATTTGCTTTTTAGCAGATGGCTCGTTGAGTCATCAGTGTTGCAAATAAAAGCTTTGCGC |
| GCTATTGTTCAAGCACGTACCTTTGACGGGTTAGCTTGAGTGACGCTGCTTGTGCTTGCA |
| ATTGCCAGGCTGCTGCCGCATGCCTCTAGCATGAAGTCAGGCAAGTGGA |
| >GS_F4.1 |
| AACCAATGGCCTCCTGAACGTACGTTGCACTCTTGGGATTTCCTGAGAGTATGTCTGCTT |
| CAGTGCTTAGCTTTCTCAACTTTGCAAAGCAATTCCCTTATTGCCTTGCGTTCCTATGAG |
| CCATTGACAATCATGGTCCATGGCTTGTTGACCAACCGGTTCCTGCAAAGCTTTGCGCGC |
| TGTTGTTCAAGCTTTTCTGCTTTGAGTGACGCTGCTGACGCTTGCAACCGCCGGGGTGCC |
| CATGCACATCTCTAGCATGAAGTCAGACAAGCAAA |
| >GS_F4.1a |
| AACCAATGGCCTCCTGAACGTACGTTGCACTCTTGGGATTTCCTGAGAGTATGTCTGCTT |
| CAGTGCTTAGCTGTCTCAACTTTGCAAAGCAATTCCCTGATTGCCTTGCGTTCCTATGAG |
| CCATCGACAATCATGGTCCATGGCTTGTTGACCAACCGGTTCCTGCAAAGCTTTGCGCGC |
| TGTTGTTCAAGCTTTTCTGCTTTGAGTGACGCTGCTGACGCTTGCAACCGCCGGGGTGCC |
| CATGCACATCTCTAGCATGAAGTCAGACAAGTAAA |
| >GS_F4.2 |
| AACCAATGGCCTCCTGAACGTACGTTGCACTCTCGGGATTTCCTGAGAGTATGTCTGCTT |
| CAGTGCTTAGCTTTCTCAACCTTGCAAAGCAATTCTTTCTTTGCCTTGCGTTCCTATGAG |
| CCATTGACAATCATCGTCAATGGCTTGTTGACCAACCGGTTCCTGCAAAGCTTTGCGCGC |
| TGTTGTTCAAGCTTCTCTGCTTTGAGTGACGCTGCTCATGCTTGCAACCGCCGGGGTGCC |
| GATGCACAACTCTAGCATGAAGTCAGACAAGTGAA |
| >GS_F4.1b |
| AACCAATGGCCTCCTGAACGTACGTTGCACTCTTGGGATTTCCTGAGAGTATGTCTGCTT |
| CAGTGCTTAGCTTTCTCAACCTTGCAAAGCAATTCCCTTATTGCTTTGCGTTCCTATGAG |
| CCATTGACAATCATGGTCCATGGCTTGTTGACCAACCGGTTCCTGCAAAGCTTTGCGCGC |
| TGTTGTTCAAGCTTTTCTGCTTTGAGTGACGCTGCTGACGCTTGCAACCGCCGGGGTGCC |
| CATGCACATCTCTAGCATGAAGTCAGACAANNNNN |
| >GS_F4.2b |
| AACCAATGGCCTCCTGAACGTACGTTGCACTCTCGGGATTTCCTGAGAGTATGTCTGCTT |
| CAGTGCTTAGCTTTCTCAACCTTGCAAAGCAATTCTTTTTTGCTTTGCGTTCCTATGAGC |
| CATTGACAATCATGGCCAATGGCTTGTTGACCAACCGGTTCCTGCAAAGCTTTGCGCGCT |
| GTTGTTCAAGCTTCTCTGCTTTGAGTGACGCTGCTCATGCTTGCAACCGCCGGGGTGCCG |
| ATGCACAACTCTAGCATGAAGTCAGACAAGTGAA |
| >GS_F4.2a=F4.2c |
| AACCAATGGCCTCCTGAACGTACGTTGCACTCTCGGGATTTCCTGAGAGTATGTCTGCTT |
| CAGTGCTTAGCTTTCTAAACCTTGCAAAGCAATTCTTTTTTTGCTTTGCGTTCCTATGAG |
| CCATTGACAATCATGGCCAATGGCTTGTTGACCAACCGGTTCCTGCAAAGCTTTGCGCGC |
| TGTTGTTCAAGCTTCTCTGCTTTGAGTGACGCTGCTCATGCTTGCAACCGCCGGGGTGCC |
| GATGCACAACTCTAGCATGAAGTCAGACAAGTGAA |
| >GS_F4.3 |
| AACCAATGGCCTCCTGAACGTACGTTGCACTCTCGGGATTTCCTGAGAGTATGTCTGCTT |
| CAGTGCTTAGCTTTCTCAACCTTGCAAAGCAATGTTTCTTTTTGCCTTGCGTTCCTATGA |
| GCCATTGACAATCATGGTCAATGGCTTGTTGACCAACCGGTTCCTGCGAAGCTTTGCGCG |
| CTGTTGTTCAAGCTTTTCTGCTTTGAGTGACGCTGCTCATGCTTGCAACCGCCGGGGTGC |
| CGACGCACATCTCTAGCATGAAGTCAGACAAGTGAA |
| >GS_F4.3a |
| AACCAATGGCCTCCTGAACGTACGTTGCACTCTCGGGATTTCCTGAGAGTATGTCTGCTT |
| CAGTGCTTAGCTTTCTCAACCTTGCAAAGCAATGTTTCTTTTTGCTTTGCGTTCCTATGA |
| GCCATTGACAATCATGGTCAATGGCTTGTTGACCAACCGGTTCCTGCGAAGCTTTGCGCG |
| CTGTTGTTCAAGCTTTTCTGCTTTGAGTGACGCTGCTCATGCTTGCAACCGCCGGGGTGC |
| CGACGCACATCTCTAGCATGAAGTCAGACAAGTGAA |
| >GS_F4.3b |
| AACCAATGGCCTCCTGAACGTACGTTGCACTCTCGGGATTTCCTGAGAGTATGTCTGCTT |
| CAGTGCTTAGCTTTCTCAACCTTGCAAAGCAATGTTTCTTTTTGCCTTGCGTTCCTATGA |
| GCCATCGACAATCATGGTCAATGGCTTGTTGACCAACCGGTTCCTGCGAAGCTTTGCGCG |
| CTGTTGTTCAAGCTTTTCTGCTTTGAGTGACGCTGCTCATGCTTGCAACCGCCGGGGTGC |
| CGACGCACATCTCTAGCATGAAGTCAGACAAGTGAA |
| >GS_F4.3c |
| AACCAATGGCCTCCTGAACGTACGTTGCACTCTCGGGATTTCCTGAGAGTATGTCTGCTT |
| CAGTGCTTAGCTTTCTCAACCTTGCAAAGCAATGTTTCTTTTTGCCTTGCGTTCCTATGA |
| GCCATTGACGACCATGGTCAATGGCTTGTTGACCAACCGGTTCCTGCGAAGCTTTGCGCG |
| CTGTTGTTCAAGCTTTTCTGCTTTGAGTGACGCTGCTCATGCTTGCAACCGCCGGGGTGC |
| CGACGCACATCTCTAGCATGAAGTCAGACAAGTGAA |
| >GS_F4.4 |
| AACCAATGGCCTCCTGAACGTACGTTGCACTCTCGGGATTTCCTGAGAGTATGTCTGCTT |
| CAGTGCTTAGCTTTCTCCACCTTGCAAGCAAATTGTGTTTTTGCCTTGCGTCCCTATGAG |
| CCATTGACGGTCACGTCCAATGGCTTGTTGACCAGTCGGTTCCTGCAAAGCTTTGCGCGC |
| TGTTGTTCAAGCTTTTTCTGCTTTGAGTGACGCTGCTCATGCTTGCAACCGCCGGGGTGC |
| TGATGCACATCTCTAGCATGAAGTCAGACAAGTGAA |
| >GS_F4.4a |
| AACCAATGGCCTCCTGAACGTACGTTGCACTCTCGGGATTTCCTGAGAGTATGTCTGCTT |
| CAGTGCTTAGCTTTCTCCACCTTGCAAGCAAATTTTGTTTTGCCTTGCGTTCCTATGAGC |
| CATTGACAATCACGGCCAATGGCTTGTTGACCAGCCGGTTCCTGCAAAGCTTTGCGCGCT |
| GTTGTTCAAGCTTTTTCTGCTTTGAGTGACGCTGCTCATGCTTGCAACCGCCGGGGTGCT |
| GATGCACATCTCTAGCATGAAGTCAGACAAGTGAA |
| >GS_F4.4b |
| AACCAATGGCCTCCTGAACGTACGTTGCACTCTCGGGATTTCCTGAGAGTATGTCTGCTT |
| CAGTGCTTAGCTTTCTCCACCTTGCAAGCAAATTGTGTTTTGCCTTGCGTTCCTATGAGC |
| CATTGACGATCACGTCCAATGGCTTGTTGACCAGCCGGTTCCTGCAAAGCTTTGCGCGCT |
| GTTGTTCAAGCTTTCCGCTTTGAGTGACGCTGCTCATGCTTGCAACCGCCGGGGTGCTGA |
| TGCACATCTCTAGCATGAAGTCAGACAAGTGAA |
| >GS_F4.5 |
| AACCAATGGCCTCCTGAACGTACGTTGCACTCTCGGGATTTCCTGAGAGTATGTCTGCTT |
| CAGTGCTTAGCTTTCTCAACCTTGCAAGCAAATTTTGATTTGCCTTGCGTTCCTATGAGC |
| CATTGGCAAGCATGGCCCAATGGCTCGTTGACCAGCCGGTTCCTGCAAAGCTTTGCGCGC |
| TGTTGTTCAAGCTTTCTTTCAGCTTTGAGTGACGCTGCTCACGCTTGCAACCGCAGGGGT |
| GCTGACGCACATCTCTAGCATGAAGTCAGACAAGTGAA |
| >GS_F4.6 |
| AACCAATGGCCTCCTGAACGTACGTTGCACTCTCGGGATTTCCTGAGAGTATGTCTGCTT |
| CAGTGCTTAGCTTTCTCAACCTTGCAAAGCAATTCTTCTTTTGCCTTGCGCTCCTATGAG |
| CCATTGACAATCATGGCCAATGGCTTGTTGACCAACCGGTTCCTGCAAAGCTTTGCGCGC |
| TGTTGTTCAAGCTTTTCTGCTTTGAGTGACGCTGCTCACGCTTGCAACCGCCGGGGTGCC |
| CATGCACATCTCTAGCATGAAGTCAGACAAGTACA |
| >GS_F4.7 |
| AACCAATGGCCTCCTGAACGTACGTTGCACTCTCGGGATTTCCTGAGAGTATGTCTGCTT |
| CAGTGCTTAGCTTTCTCAACCTTGCAAAGCATGCGTGTTTTTTTGCCTTGCGTTCCTACG |
| AGCCATTGACAACCACGGTCAGTGGCTTGTTGACCAACCGGTTCCTGCAAAGCTTTGCGC |
| TCTGTTGTTCAAGCTTTTCTGCTTTGAGTGACGCTGCTCACGCTTGCAACCGCCGGAGCG |
| CCGATGCACATCTCTAGCATGAAGTCAGACAAGTGAA |
| >GS_F4.8 |
| AACCAATGGCCTCCTGAACGTACGTTGCACTCTCGGGATTTCCTGAGAGTATGTCTGCTT |
| CAGTGCTTAGCTTTCTCAACCTTGCAAAGCAAAGTCTTTTCTTTGCTGTTGCGCTCCTAT |
| GGGCCATTGACAATCATGGTCAATGGCTTGTTGACTAACCGGTTCCTGCAAAGCTTTGCG |
| CGCTGTTGTTCAAGCTTCTCTGCTCTGAGTGACGCTGCTCACGCTTGCAACCGCCGGGGT |
| GCTGAAGCACAACTCTAGCATGAAGTCAGACAAGTGAA |
| >GS_F4.8a |
| AACCAATGGCCTCCTGAACGTACGTTGCACTCTCGGGATTTCCTGAGAGTATGTCTGCTT |
| CAGTGCTTAGCTTTCTCAACCTTGCAAAGCAAAGTCGTTTCTTTGCTGTTGCGCTCCTAT |
| GGGCCATTGACAATCATGGTCAATGGCTTGTTGACTAACCGGTTCCTGCAAAGCTTTGCG |
| CGCTGTTGTTCAAGCTTCTCTGCTCTGAGTGACGCTGCTCACGCTTGCAACCGCCGGGGT |
| GCTGAAGCACAACTCTAGCATGAAGTCAGACAAGTGAA |
| >GS_F4.8b |
| AACCAATGGCCTCCTGAACGTACGTTGCACTCTCGGGATTTCCTGAGAGTATGTCTGCTT |
| CAGTGCTTAGCTTTCTCAACCTTGCAAAGCAAAGTCGTTTCTTTGCTGTTGCGCTCCTAT |
| GGGCCATTGCCAATCATGGTCGATGGCTTGTTGACTAACCGGTTCCTGCAAAGCTTTGCG |
| CGCTGTTGTTCAAGCTTCTCTGCTCTGAGTGACGCTGCTCACGCTTGCAACCGCCGGGGT |
| GCTGAAGCACAACTCTAGCATGAAGTCAGACAAGTGAA |
| >GS_F4.8c |
| AACCAATGGCCTCCTGAACGTACGTTGCACTCTCGGGATTTCCTGAGAGTATGTCTGCTT |
| CAGTGCTTAGCTTTCTCAACCTTGCAAAGCAAAGTCTTTTCTTTGCTGTTGCGCTCCTGT |
| GGGCCATTGGACAATCATGGTCAATGGCTTGTTGACTAACCGGTTCCTGCAAAGCTTTGC |
| GCGCTGTTGTTCAAGCTTCTCTGCTCTGAGTGACGCTGCTCACGCTTGCAACCGCCGGGG |
| TGCTGAAGCACAACTCTAGCATGAAGTCAGACAAGTGAA |
| >GS_F5.1 |
| AACCAATGGCCTCCTGAACGTACGTTGCACTCTTGGGATTTCCTGAGAGTATGTCTGCTT |
| CAGTGCTTAGCTTGCCCAATCTTGCGGATAGACTTTGTTTCTGTCTTGCGCCCCTGTGAG |
| CCATTGAGCGTCTAGTCAATGGCTTATTGAATGATTCGGTCTTGCAAAAGCTTTGCGCGC |
| TTCTATTCAAGATTCCACCTTGGAGTGGTATTGCTTGAGTGACGCTGCTCATGCTTGCAA |
| CTGCTGGGATGCTAACGCATGCCTCTAGCATGAAGTCAGACAAGCGAA |
| >GS_F5.1a |
| AACCAATGGCCTCCTGAACGTACGTTGCACTCTTGGGATTTCCTGAGAGTATGTCTGCTT |
| CAGTGCTTAGCTTGCCCAATCTTGCGGATAGACTTTGTTTCTGTCTTGCGCCCCTGTGAG |
| CCATTGAGCGTTTAGTCAATGGCTTATTGAATGATTCGGTCTTGCAAAAGCTTTGCGCGC |
| TTCTATTCAAGATTCCACCTTGGAGTGGTATTGCTTGAGTGACGCTGCTCATGCTTGCAA |
| CTGCTGGGATGCTAACGCATGCCTCTAGCATGAAGTCAGACAAGCGAA |
| >GS_F5.1b |
| AACCAATGGCCTCCTGAACGTACGTTGCACTCTTGGGATTTCCTGAGAGTATGTCTGCTT |
| CAGTGCTTAGCTTGCCCAATCTTGCGGATAGACTTTGTTTCTGTCTTGCGCCCCTGTGAG |
| CCATTGAGCGTTTAGTCAATGGCTTATTCAATGATTCGGTCTTGCAAAAGCTTTGCGCGC |
| TTCTATTCAAGATTCCACCTTGGAGTGGTATTGCTTGAGTGACGCTGCTCATGCTTGCAA |
| CTGCTGGGATGCTAACGCATGCCTCTAGCATGAAGTCAGACAAGCGAA |
| >GS_F5.1c |
| AACCAATGGCCTCCTGAACGTACGTTGCACTCTTGGGATTTCCTGAGAGTATGTCTGCTT |
| CAGTGCTTAGCTTGCCCAATCTTGCGGATAGATTTTATTTCTGTCTTGCGCCCCTGTGAG |
| CCATTGAATGTCTAGTCAATGGCTTATTGAATGATTTGGTCTTGCAAAAGCTTTGCGCGC |
| TTCTATTCAAGATTCCACCTTGGAGTGGTATTGCTTGAGTGACGCTGCTCATGCTTGCAA |
| CTGCCGGGATGCTAACGCATGCCTCTAGCATGAAGTCAGACAAGCGAA |
| >GS_F5.2=F5.2a |
| AACCAATGGCCTCCTGAACGTACGTTGCACTCTTGGGATTTCCTGAGAGTATGTCTGCTT |
| CAGTGCTTAGCTTGCCCAATCTTGCGGACAGATTTTGTTTCTGCCCTGCGCCCCTGTGAG |
| CCATTGAATGTCTACCCAATGGCTTATTGAATGATTTGGTCTTGCAAAAGCTTTGCGCGC |
| TGCTATTCAAGATTCCACCTTAAAGTGGTATTGCTTGAGTGACGCTGCTTATGCTTGCAG |
| CTGCTGGGATGCTAGCGCATGCCTCTAGCATGAAGTCAGACAAGCGAA |
| >GS_F5.2b |
| AACCAATGGCCTCCTGAACGTACGTTGCACTCTTGGGATTTCCTGAGAGTATGTCTGCTT |
| CAGTGCTTAGCTTGCCCAATCTTGCGGACAGATTTTGTTTCTGTCCTGCGCCCCTGTGAG |
| CCATTGAATGTCTACCCAATGGCTTATTGAATGATTTGGTCTTGCAAAAGCTTTGCGCGC |
| TGCTATTCAAGATTCCACCTTAAAGTGGTATTGCTTGAGTGACGCTGCTTATGCTTGCAG |
| CTGCTGGGATGCTAGCGCATGCCTCTAGCATGAAGTCAGACAAGCGAA |
| >GS_F5.2c |
| AACCAATGGCCTCCTGAACGTACGTTGCACTCTTGGGATTTCCTGAGAGTATGTCTGCTT |
| CAGTGCTTAGCTTGCCCAATCTTGCGGACAGATTTTGTTTCTGTCCTGCGCCCCTGTGAG |
| CCATTGAATGCCTACTCAATGGCTTATTGAATGATTTGGTCTTGCAAAAGCTTTGCGCGC |
| TGCTATTCAAGATTCCACCTTAAAGTGGTATTGCTTGAGTGACGCTGCTTATGCTTGCAG |
| CTGCTGGGATGCTAGCGCATGCCTCTAGCATGAAGTCAGACAAGCGAA |
| >GS_F5.2d |
| AACCAATGGCCTCCTGAACGTACGTTGCACTCTTGGGATTTCCTGAGAGTATGTCTGCTT |
| CAGTGCTTAGCTTGCCCAATCTTGCGGACAGATTTTGTTTCTGTCCTGCGCCCCTGTGAG |
| CCATTGAATGCCTGCTCAATGGCTTATTGAATGATTTGGTCTTGCAAAAGCTTTGCGCGC |
| TGCTATTCAAGATTCCACCTTAAAGTGGTATTGCTTGAGTGACGCTGCTTATGCTTGCAG |
| CTGCTGGGATGCTAGCGCATGCCTCTAGCATGAAGTCAGACAAGCGAA |
| >GS_F5.2e |
| AACCAATGGCCTCCTGAACGTACGTTGCACTCTTGGGATTTCCTGAGAGTATGTCTGCTT |
| CAGTGCTTAGCTTGCCCAATCTTGCGGACAGATTTTGTTTCTGTCTTGCGCCCCTGTGAG |
| CCATTGAATGTCTACTCAATGGCTTATTGAATGATTTGGTCTTGCAAAAGCTTTGCGCGC |
| TGCTATTCAAGATTCCACCTTAAAGTGGTATTGCTTGAGTGACGCTGCTTATGCTTGCAG |
| CTGCTGGGATGCTAGCGCATGCCTCTAGCATGAAGTCAGACAAGCGAA |
| >GS_F5.2f |
| AACCAATGGCCTCCTGAACGTACGTTGCACTCTTGGGATTTCCTGAGAGTATGTCTGCTT |
| CAGTGCTTAGCTTGCCCAATCTTGCGGACAGATTTTGTTTCTGTCTTGCGCCCCTGTGAG |
| CCATTGAGCGTCTATTCAGTGGCTTATTGAATGATTTGGTCTTGCAAAAGCTTTGCGCGC |
| TGCTATTCAAGATTCCACCTTAAAGTGGTATTGCTTGAGTGACGCTGCTTATGCTTGCAA |
| CTGCTGGGATGCTAGCGCATGCCTCTAGCATGAAGTCAGACAAGCGAA |
| >GS_G1 |
| AACCAATGGCCTCCTGAACGCGCATTGCACTCTTGGGCTTTCCTGAGAGTATGTTTGCTT |
| CAGTGCTTCTTTTGCTCAACCCTTGCAAGGTCTGGCAGCGCAATGCCCCCTTGAGCCTAG |
| GCATGTCGTTGGCGCATCTGCCAATGACAAGCGACCTCCATGGCTTGTGCAAGCATGCAC |
| GTGCTTATTGTTGTTTTCAGAGCAAACTTCACCACCATGGGTGTGGGCAACGTGGCTGAT |
| GCTTGAGCACGCGCCGGTGCGTTGCTTGCACCTTCTCCATAGCATGAA |
| >GS_G2 |
| AACCAATGGCCTCCTGAACGCGCATTGCACTCTTGGGCTTTCCTGAGAGTATGTTTGCTT |
| CAGTGCTTCTTTTGCTCAACCTTGCAAGGTCTGGCAATGCAATGCCCCCTTGAGCCTCGG |
| CTTGTTGTTGCACATCTGCCAGTGACAAGCGACTTCCATGGCCTGTGCAAGCATGCACGT |
| GCTTTTTTGTCGTTTTCAGAGCAAACTTCTTCGCCATGGGCGGGGGCAATGTGGCTGATG |
| CTTGAGCACGCGCCGGTGCGTTGCTTGCACCTCCTCCATAGCATGAA |
| >GS_G2a |
| AACCAATGGCCTCCTGAACGCGCATTGCACTCTTGGGCTTTCCTGAGAGTATGTTTGCTT |
| CAGTGCTTCTTTTGCTCAACCTTGCAAGGTCTGGCAATGTAATGCCCCCTTGAGCCTCGG |
| CTTGTTGTTGCACATCTGCCAGTGACAAGCGACTTCCATGGCCTGTGCAAGCATGCACGT |
| GCTTTTTTGTCGTTTTCAGAGCAAACTTCTTCGCCATGGGCGGGGGCAATGTGGCTGATG |
| CTTGAGCACGCGCCGGTGCGTTGCTTGCACCTCCTCCATAGCATGAA |
| >GS_G3 |
| AACCAATGGCCTCCTGAACGCGCATTGCACTCTTGGGCTTTCCTGAGAGTATGTTTGCTT |
| CAGTGCTTCTTTTGCTCCACCGTTGCAAGGTTTGGCAGCGCAATGCCTCCTTGTGCCTCG |
| GCGTGTTGTTGGCGTCTCTGCCAACGACGTGCGACCAGCGTGGCCTTTGTGCAAGCATGC |
| ACGTGCTTTGTTGTTTCACTGCAGCCATTCTCCGGAATATGCGTGGGCGACGTGGCTGAT |
| GCTTGCGGACGCGCTACTGTGCTGCTTGCACTTCTTCCATAGCATGAA |
| >GS_G4 |
| AACCAATGGCCTCCTGAACGCGCATTGCACTCTTGGGCTTTCCTGAGAGTATGTTTGCTT |
| CAGTGCTTCTTCTGTTCCTCCATGGCGAGGGCTTTGGCAAGCTATGCCCCCTCCTGCCTT |
| GGCGTGCCGTTGGTGTGTTTGCCAATGACATGCGACATGCGTGGCCTTTGTGCAGGCAAG |
| CACGCGCGTTGTTGTTTCACGCACCATTCCTCAGAATGTCGTGTGTGTGTGGGCGACGTG |
| ACTGATGCTTGAGGACGCGCTGGAGTGCTGATGCACTTCCCATAGCATGAA |
| >GS_H1 |
| AACCAATGGCCTCCTGAACGTGCGTTGCACTCTCGGGATTTCCTGAGAGTATGTCTGCTT |
| CAGTGCTTAGCTTACCCAACTTTGCAATCAGGCTGACTACCAAGCCTGCTTTTTGCGTTC |
| CTATGAGCTATTGCGCTTCCTTTGCCAATGGCTTGTTGATTGGTAGGTTCCTGCAAAATG |
| CTTTGCGCGCTGTTATTCAAGTTTCGCCTGCACGGCTTTGCTTGAGTGACGCTGCTCATG |
| CGTGCAACCGCTGGGATGCGCTCCGCGCATGCCTCTAGCATGAAGTCAGACAA |
| >GS_H1a |
| AACCAATGGCCTCCTGAACGTGCGTTGCACTCTCGGGATTTCCTGAGAGTATGTCTGCTT |
| CAGTGCTTAGCTTACCCAACTGTGCAATCAGGCTGACTACCAAGCCTGCTTTTTGCGTTC |
| CTATGAGCTATTGCGCTTCCTTTGCCAATGGCTTGTTGATTGGTAGGTTCCTGCAAAATG |
| CTTTGCGCGCTGTTATTCAAGTTTCGCCTGCACGGCTTTGCTTGAGTGACGCTGCTCATG |
| CGTGCAACCGCTGGGATGCGCTCCGCGCATGCCTCTAGCATGAAGTCAGACAA |
| >GS_H1b |
| AACCAATGGCCTCCTGAACGTGCGTTGCACTCTCGGGATTTCCTGAGAGTATGTCTGCTT |
| CAGTGCTTAGCTTACCCAACTTTGCAATCAGGCTGACTACCAAGCCTGCTTTTTGCGTTC |
| CTATGAGCTATTGCACTTCCTTTGCCAATGGCTTGTTGATTGGTAGGTTCCTGCAAAATG |
| CTTTGCGCGCTGTTATTCAAGTTTCGCCTGCACGGCTTTGCTTGAGTGACGCTGCTCATG |
| CGTGCAACCGCTGGGATGCGCTCCGCGCATGCCTCTAGCATGAAGTCAGACAA |
| >GS_H2 |
| AACCAATGGCCTCCTGAACGTGCGTTGCACTCTCGGGATTTCCTGAGAGTATGTCTGCTT |
| CAGTGCTTAGCTTACCCAACTTTGCAAGCAGATTGATTGAATGTCTGCCTTGCGTTCCTA |
| TGAGCTATTGCGCTTCCTTTGCCAATGGCTTGTTGATTGGTAGGTTCCTGCAAAATGCTT |
| TGCGCGCTGTTATTCAAGTTTCGCCTGCACGGCTTTGCTTGAGTGACGCTGCTCATGCAT |
| GCAACCGCTGGGATGCTGGCGCATGCCTCTAGCATGAAGTCAGACAA |
| >GS_H3 |
| AACCAATGGCCTCCTGAACGTGCGTTGCACTCTCGGGATTTCCTGAGAGTATGTCTGCTT |
| CAGTGCTTAGCTTACCCAACTTTGCAAGCAGATTGAAATTTCTGTCTTGCGTTCCTATGA |
| GCTATTGCGCTCCTCGTGCCAATGGCTTGTTGATTGGCTGGTTTCTGCAAAATGCTTTGC |
| GCGCTGTTATTCAAGCTTTGCCCATGTGGCTCCGCTTGAGTGACGCTGCTCATGCATACA |
| ACCGCTGGGATGCTGGCGCATGCCTCTAGCATGAAGTCAGGCAA |
| >GS_H4 |
| AACCAATGGCCTCCTGAACGTGCGTTGCACTCTCGGGATTTCCTGAGAGTATGTCTGCTT |
| CAGTGCTTAGCTTACCCAACTTTGCAAGCAGGTTGTCTGCCAAGCCTGCCTTTGCGTTCC |
| TATGAGCTATTGTGCTTCCTTTGCCAATGGCTTGTTGATTGGTAGGNTCCTGCAAAATGC |
| TTTGCGCGCTGTTATTCAAGTTTCGCCTGCACGGCTTTGCTTGAGTGACGCTGCTTATGC |
| GTGCGACCGCTGGGATGCTCGCCGAGCATGCCTCTAGCATGAAGTCAGACAA |
| >GS_H4a |
| AACCAATGGCCTCCTGAACGTGCGTTGCACTCTCGGGATTTCCTGAGAGTATGTCTGCTT |
| CAGTGCTTAGCTTACCCAACTTTGCAAGCAGGTTGTCTGCCAAGCCTGCGTTTGCGTTCC |
| TATGAGCTATTGTGCTTCCTTTGCCAATGGCTTGTTGATTGGTAGGTTCCTGCAAAATGC |
| TTTGCGCGCTGTTATTCAAGTTTCGCCTGCACGGCTTTGCTTGAGTGACGCTGCTTATGC |
| GTGCGACCGCTGGGATGCTCGCCGAGCATGCCTCTAGCATGAAGTCAGACAA |
| >GS_H5 |
| AACCAATGGCCTCCTGAACGTGCGTTGCACTCTCGGGATTTCCTGAGAGTATGTCTGCTT |
| CAGTGCTTAGCTTACCCAACTTTGCAAGCAGGCTTAAAGTCTGCGTCGCGTTCCTATGAG |
| CTATTGTGCTTCCTTTGCCAATGGCTTGTTGAGTGGTAGGTTCCTGCAAAATGCTTTGCG |
| CGCTGTTATTCAAGTTTCGCCTGCACGGCTTTGCTTGAGTGACGCTGCTCATGCATGCAA |
| CCGCTGGGGTGCGCTCTGCGCATGCCTCTAGCATGAAGTCAGACAA |
| >GS_H5a |
| AACCAATGGCCTCCTGAACGTGCGTTGCACTCTCGGGATTTCCTGAGAGTATGTCTGCTT |
| CAGTGCTTAGCTTACCCAACTTTGCAAGCAGGCTTAAAGTCTGCGTCGCGTTCCTATGAG |
| CTATTGTGCTTCTTTTGCCAATGGCTTGTTGAGTGGTAGGTTCCTGCAAAATGCTTTGCG |
| CGCTGTTATTCAAGTTTCGCCTGCACGGCTTTGCTTGAGTGACGCTGCTCATGCATGCAA |
| CCGCTGGGGTGCGCTCTGCGCATGCCTCTAGCATGAAGTCAGACAA |
| >GS_H5b |
| AACCAATGGCCTCCTGAACGTGCGTTGCACTCTCGGGATTTCCTGAGAGTATGTCTGCTT |
| CAGTGCTTAGCTTACCCAACTTTGCAAGCAGGCTTAAAGTCTGCGTCGCGTTCCTATGAG |
| CTATTGTGCTTCTTCCTTTGCCAATGGCTTGTTGAGTGGTAGGTTCCTGCAAAATGCTTT |
| GCGCGCTGTTATTCAAGTTTCGCCTGCACGGCTTTGCTTGAGTGACGCTGCTCATGCATG |
| CAACCGCTGGGGTGCGCTCCGCGCATGCCTCTAGCATGAAGTCAGACAA |
| >GS_H6 |
| AACCAATGGCCTCCTGAACGTGCGTTGCACTCTCGGGATTTCCTGAGAGTATGTCTGCTT |
| CAGTGCTTAGCTTGCAGATTTCTAGTCTTGAGCCATTGTCTTCTTTGCCAATGGCTTGTT |
| GAGTGGTAGGTTCCTGCAAAATGCTTTGCGCGCTGTTATTCAAGTTTCGCCTGCACGGCT |
| TTGCTTGAGTGACGCTGCTCATGCGTGCAACCGCTGGGATGCGCACACGCGCATGCCTCT |
| AGCATGAAACAAGCGAA |
| >GS_I1 |
| AACCAATGGCCTCCTGAACGCTCATTGCACCCTTGGGATTTCCTGAGGGCATGTCTGCTT |
| CAGTGCTTAGCTTTTACACCTTCGTGCGGGCGCGATGTTTTCGTGTCCTGCACTCCTGCA |
| AGCCATCGCTCAGATTTGCTTCTGATGGCTTGTTGAATGATTGGCTGTTTTGCAAGCTCA |
| AGCGCTTTGTGATTCATAGCAAACCTATGGGATTCGCTTGGGTCGCGCTGCTGATGCCTA |
| CAGCCTTCAGCATGTGAAACCGCATGCATCTTAGCATGAAGTCAGACAAGAGAACCCGCT |
| GAATTTAAGCATATAAGTAA |
| >GS_I2 |
| AACCAATGGCCTCCTGAACGCTCATTGCACCCTTGGGATTTCCTGAGGGCATGTCTGCTT |
| CAGTGCTTAGCTTTTACACCTTCGTGCGGGCGCGATGTTTTTGTGTCCTGCACTCCTGCA |
| AGCCATCGCTCAGATTTGTTTCTGATGGCTTGTTGAATTATTAGCTGTTTTGCAAGCTCA |
| AGCGCTTTGTGATTCATAGCAAACCTACGGGATTCGCTTGGGTCGCGCTGCTGATGCCTA |
| CAGCCTTCAGCATGTGAAGCCGCATGCATCTTAGCATGAAGTCAGACAAGAGAACCCGCT |
| GAATTTAAGCATATAAGTAA |
| >GS_I3 |
| AACCAATGGCCTCCTGAACGCTCATTGCACCCTTGGGATTTCCTGAGGGCATGTCTGCTT |
| CAGTGCTTAGCTTTTACACCTTCCTGCAGGCGCGATGTTTTTGCGTTTTGCACTCCTGCA |
| AGCCATCGCTCAGATTTGCTTCTGATGGCTTGTTGAATTATTGGCTGTTTTGCAAGCTCA |
| AGCGCTTTGTGATTCATAGCAAACCCACGGGATTCGCTTGGGTCGCGCTGCTGATGCCTA |
| CAGCCTTCAGCATGTGAAGCCGCATGCATCTTAGCATGAAGTCAGACAAGAGAACCCGCT |
| GAATTTAAGCATATAAGTAA |
| >GS_I4 |
| AACCAATGGCCTCCTGAACGCTCATTGCACCCTTGGGATTTCCTGAGGGCATGTCTGCTT |
| CAGTGCTTAGCTTTTACACCTTCCTGCAGGCGCGATGTTTTTGCGTCTTGCACTCCTGCA |
| AGCCATCGCTCAGATTTGCTTCTGATGGCTTGTTGAATTATTGGCTGTTTTGCAAGCTCA |
| AGCGCTTTGTGATTCATAGCAAACCCACGGGATTCGCTTGGGTCGCGCTGCTGATGCCTA |
| CAGCCTTCAGCATGTGAAGCCGCATGCATCTTAGCATGAAGTCAGACAAGAGAACCCGCT |
| GAATTTAAGCATATAAGTAA |
